# Supplementary material for: A RASSF1A-HIF1α loop drives Warburg effect in cancer and pulmonary hypertension
Source: Nat Commun. 2019 May 13;10:2130. doi: 10.1038/s41467-019-10044-z (PMC6513860; doi:10.1038/s41467-019-10044-z)
Supplement: Supplementary file 1 — Supplementary Information [file 41467_2019_10044_MOESM1_ESM.pdf]

Supplementary Information

A RASSF1A-HIF1α loop driving Warburg effect in cancer and Pulmonary hypertension

Dabral et al.

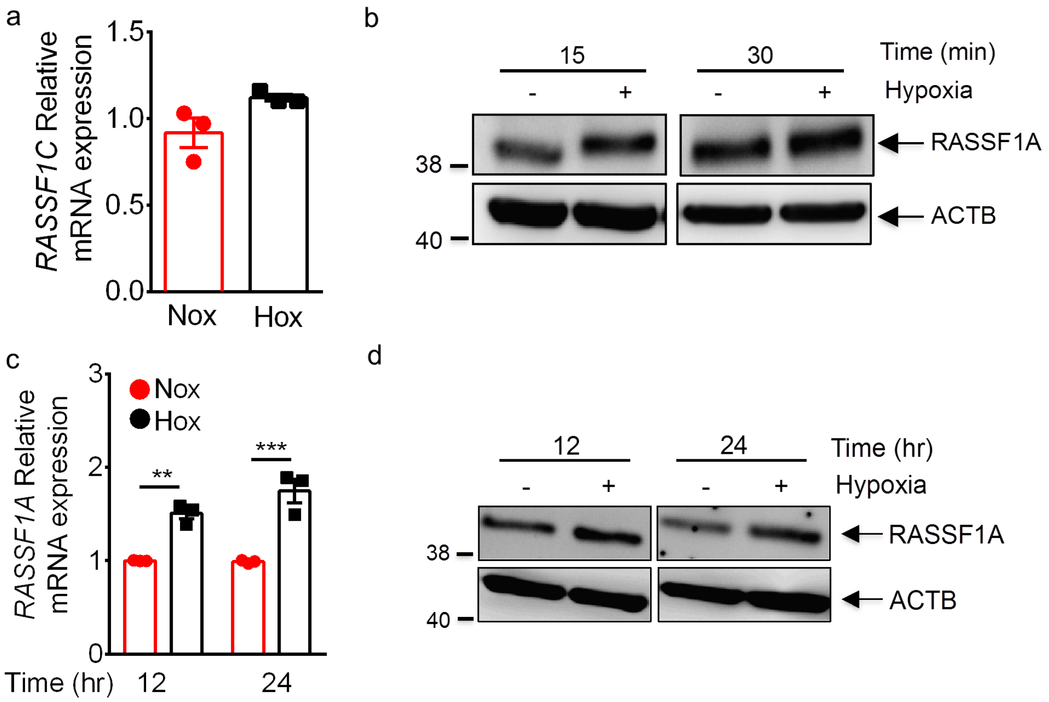

Supplementary Fig. 1: RASSF1A is upregulated in hypoxia exposed pulmonary artery adventitial fibroblasts. (a) Human PASMCs were exposed to 20% (Normoxia) or 1% O_2 (Hypoxia) for 24 hr. Cell lysates were subjected to real time PCR for RASSF1C. **(b–d)** Human PAAFs were exposed to 20% (Normoxia) or 1% O_2 (Hypoxia) for indicated intervals. Cell lysates from each time point were subjected to **(b, d)** western blotting for RASSF1A and ACTB and **(c)** real time PCR for *RASSF1A*. **P* < 0.05, ****P* < 0.001 compared to Normoxia (Nox), unpaired Student's *t*-test. n = 2 independent experiments from 2 biological replicates**.**

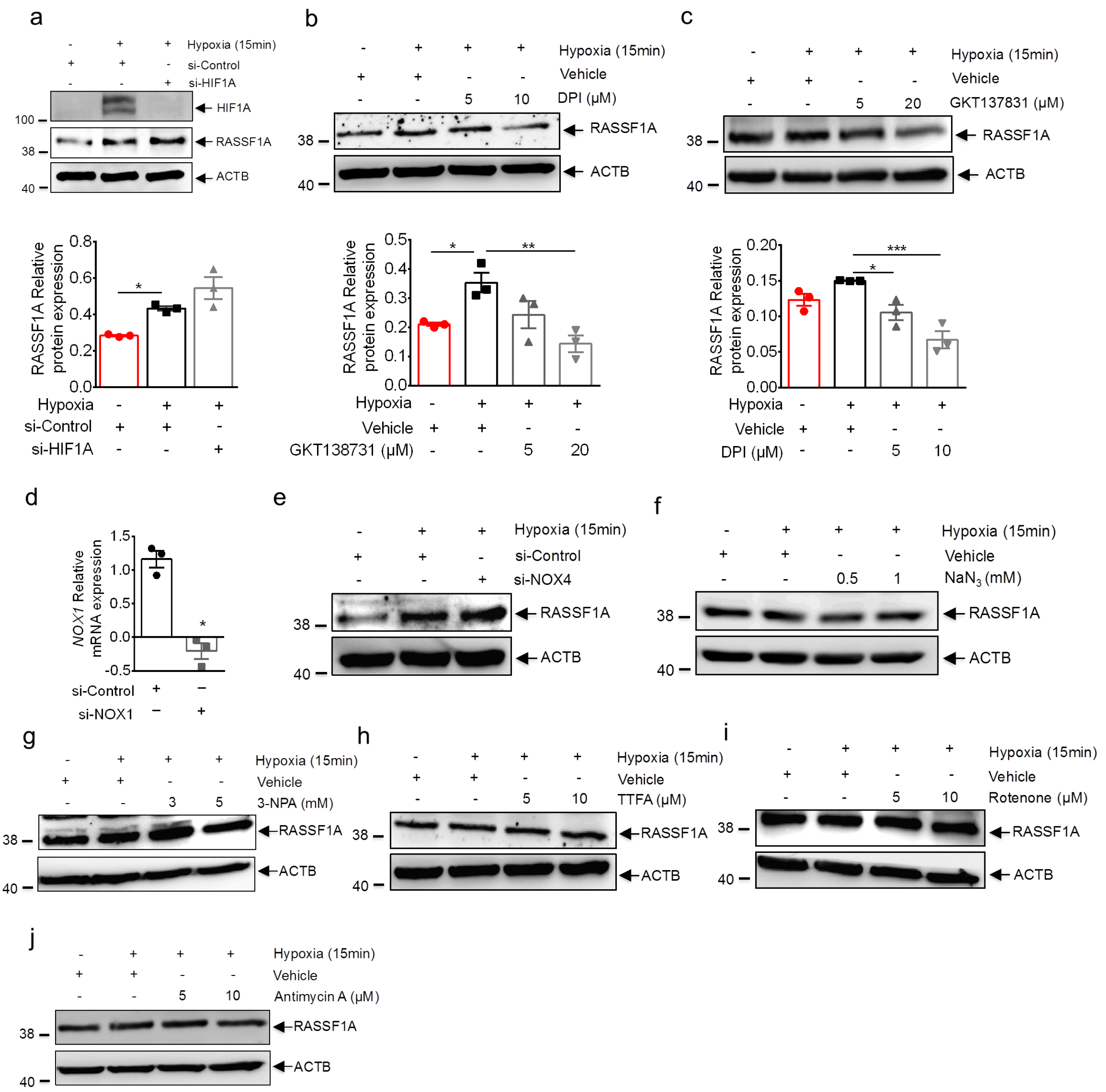

Supplementary Fig. 2: Influence of HIF1A, ROS, NOX and electron transport chain on RASSF1A protein expression. **(a)** Human PASMCs were transfected HIF1A siRNA (si-HIF1A) or control siRNA (si-Control). 24hr later, cells were exposed to hypoxia for 15min, followed by cell lysis and **(a, upper)** western blotting for HIF1A, RASSF1A and ACTB and **(a, lower)** densitometric quantification of relative RASSF1A expression. **(b, c)** Human PASMCs were pre-treated with **(b)** DPI (ROS inhibitor) or **(c)** GTK137831 (NOX inhibitor) at indicated concentrations for 1 h, followed by 15min Hypoxia exposure and cell lysates representing each dose were subjected to **(b–c, upper)** western blotting for RASSF1A and ACTB. **(b–c, lower)** Densitometric quantification of relative RASSF1A expression (RASSF1A/ACTB). *$P < 0.05$, **$P < 0.01$, ***$P < 0.001$ compared to si-Control (Hypoxia) or vehicle (Hypoxia), one-way ANOVA followed by SNK multiple comparison test. n = 3 independent experiments. **(d)** Human PASMCs were transfected with NOX1 siRNA (si-NOX1). 24hr later, RNA was isolated and real time PCRs were performed. **$P < 0.01$ compared to si-Control, unpaired Student's *t*-test. **(e)** Human PASMCs were transfected with NOX4 siRNA (si-NOX4) or control siRNA (si-Control) for 48hr, followed by 15min Hypoxia exposure. Cell lysates were subjected to western blotting for RASSF1A and ACTB. **(f–k)**

Human PASMCs were pre-treated with different inhibitors of electron chain; **(f)** sodium azide (NaN_3), **(g)** 3-sodium propionic acid (3-NPA), **(h)** thenoyltrifluoroacetone (TTFA)**,** (**i)** rotenone and **(j)** antimycin A at indicated concentrations for 1 h, followed by 15min Hypoxia exposure and cell lysates representing each dose were subjected to western blotting for RASSF1A and ACTB. Data represent mean ± s.e.m all through the figure. n = 3 independent experiments from 3 biological replicates**.**

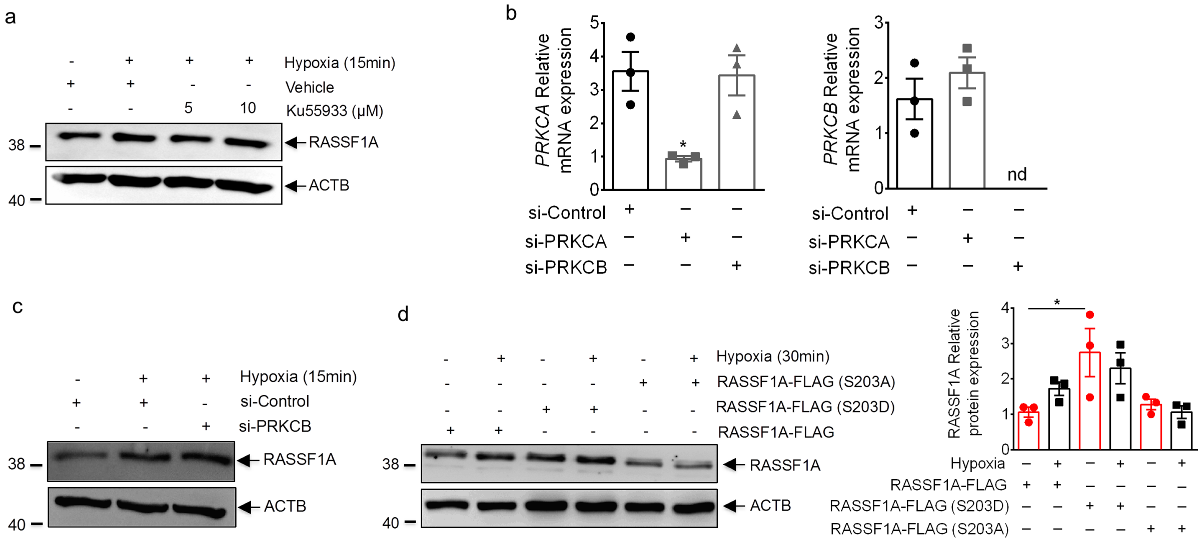

Supplementary Fig. 3: Influence of phosphorylation on RASSF1A expression. (a) Human PASMCs were pre-treated ATM kinase inhibitor (KU55933) at indicated concentrations for 1 h, followed by 15min Hypoxia exposure and cell lysates representing each dose were subjected to western blotting for RASSF1A and ACTB. **(b)** Human PASMCs were transfected with PKCα siRNA (si-PRKCA), PKCβ siRNA (si-PRKCB) or control siRNA (si-Control). 24hr later, RNA was isolated and real time PCRs were performed. ***P* < 0.01 compared to si-Control, unpaired Student's *t*-test. **(c)** Human PASMCs were transfected with si-PRKCB or si-Control for 48hr, followed by 15min Hypoxia exposure. Cell lysates were subjected to western blotting for RASSF1A and ACTB. **(d)** HEK 293 cells were transfected with plasmids indicated in top lane, and exposed to Hypoxia for 30 min. **(d, left)** Cells were lysed and subjected to western blotting for RASSF1A and ACTB, followed by **(d, right)** densitometric quantification of relative RASSF1A expression.* *P* < 0.05, ***P* < 0.01 compared to **(b)** si-Control or **(d)** RASSF1A-FLAG (Normoxia). Data represent mean ± s.e.m all through the figure. n = 3 independent experiments from 3 biological replicates for human PASMCs and n=3 independent experiments for HEK cell lines.

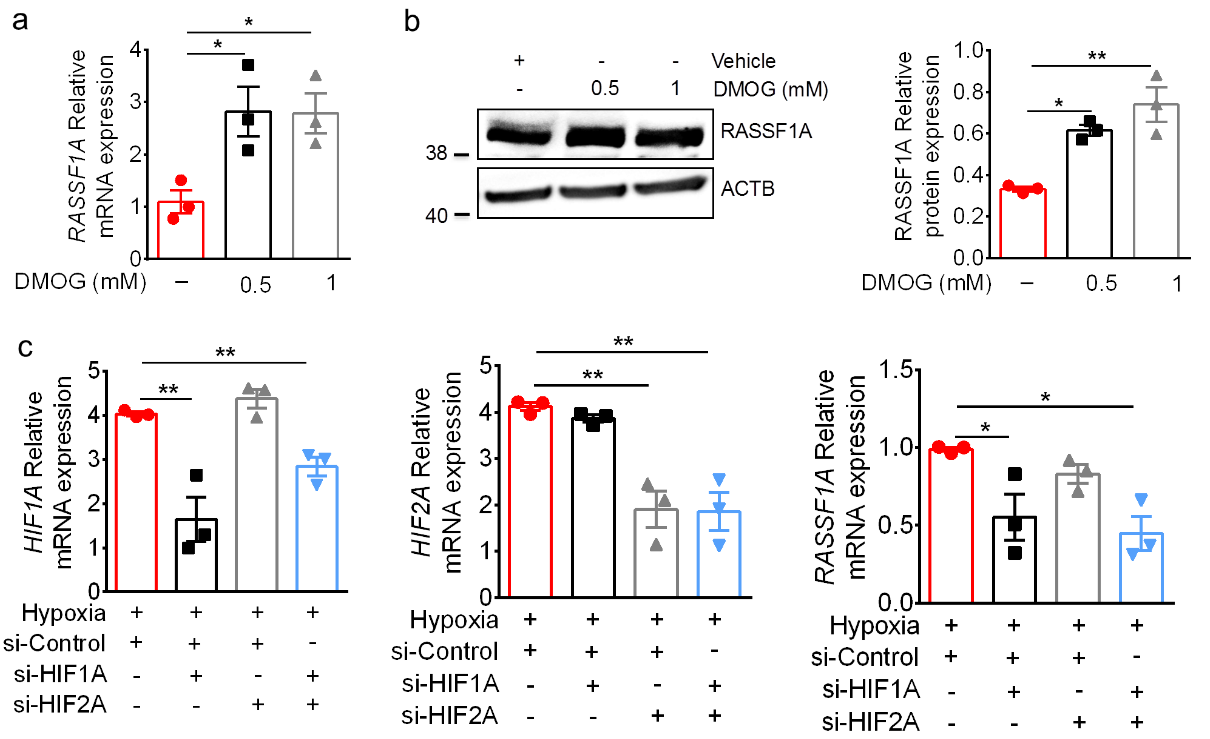

Supplementary Fig. 4: *RASSF1* is a HIF-1 target gene. (a, b) Human PASMCs were treated with indicated concentrations of prolyl hypdroxylase inhibitor, DMOG (hypoxia mimetic), followed by **(a)** real time PCR and **(b, left)** western blotting for RASSF1A and ACTB. **(b, right)** Densitometric quantification of relative RASSF1A expression. *$P < 0.05$, **$P < 0.01$ compared to control, one-way ANOVA followed by SNK multiple comparison test. **(c)** Human PASMCs were transfected with HIF-1α siRNA (si-HIF1A) or HIF-2α siRNA (si-HIF2A) or both together for 24hr, with additional 24hr hypoxia exposure. Cell lysates were subjected to real time PCRs for *HIF1A*, *HIF2A* and *RASSF1A*. **$P < 0.01$, ***$P < 0.001$ compared to si-Control (Hypoxia), one-way ANOVA followed by SNK multiple comparison test. Data represent mean ± s.e.m all through the figure. n = 3 independent experiments from 3 biological replicates**.**

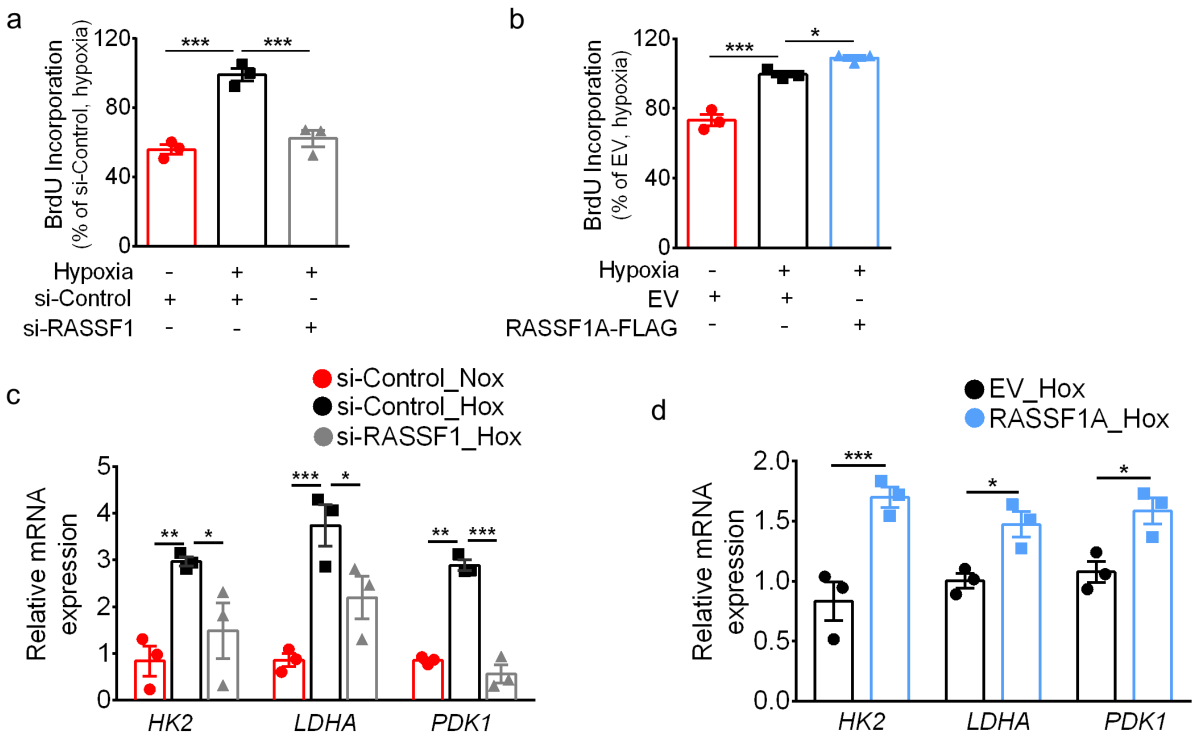

Supplementary Fig. 5: RASSF1A regulates proliferation and glycolytic metabolism of human PAAFs under hypoxia. Human PAAFs were transfected with **(a, c)** RASSF1 siRNA (si-RASSF1) and control siRNA (si-Control) or **(b, d)** RASSF1A-FLAG and empty vector (EV). **(a, b)** 24hr after transfection, cells were exposed to Normoxia or Hypoxia for 48hr and proliferation was measured by BrdU incorporation assay. **(c, d)** 24hr after transfection, cells were exposed to Normoxia or Hypoxia for 24hr. Quantitative real time RT PCRs for *HK2*, *LDHA* and *PDK1* were performed. *$P < 0.05$, **$P < 0.01$, ***$P < 0.001$ compared to **(a, c)** si-Control (Hypoxia) or **(b, d)** EV (Hypoxia), one-way ANOVA followed by SNK multiple comparison test. n = 2 independent experiments, Data represent mean ± s.e.m all through the figure. n = 3 independent experiments from 3 biological replicates.

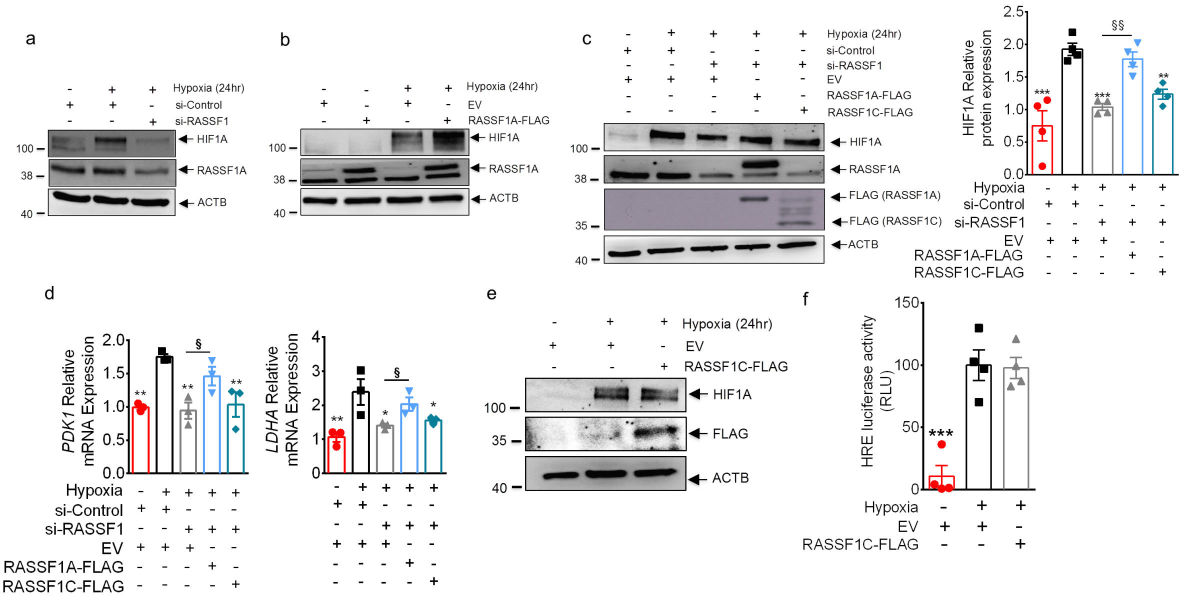

Supplementary Fig. 6: RASSF1A, not RASSF1C regulates HIF1α. (a, b) Human PAAFs were transfected with **(a)** RASSF1 siRNA (si-RASSF1) and control siRNA (si-Control) or **(b)** RASSF1A-FLAG and empty vector (EV). 24hr after transfection, cells were exposed to Normoxia or Hypoxia for 24hr. Cell lysates were subjected to western blotting for HIF1A, RASSF1A and ACTB. **(c, d)** Human PASMCs were transfected with RASSF1 siRNA (si-RASSF1) and control siRNA (si-Control). 24 hr later, cells were further transfected with EV, RASSF1A-FLAG or RASSF1C-FLAG as mentioned in the lanes above the blots. 6 hr later, cells were exposed to Hypoxia or Normoxia for further 24 hr. **(c, left)** Cell lysates were subjected to western blotting for HIF1A, RASSF1A, FLAG, and ACTB. **(c, right)** Densitometrical quantified data of HIF1A/ACTB expression ratio. **(d)** Real time PCRs for PDK1 and LDHA were performed. $^{**}P < 0.01$, $^{***}P < 0.001$ compared to si-Control (Hypoxia), $^{§§}P < 0.01$ compared to si-RASSF1+EV (Hypoxia) one-way ANOVA followed by SNK multiple comparison test. **(e)** Human PASMCs were transfected with EV or RASSF1C-FLAG and 24 hr later, cells were exposed to hypoxia for another 24 hr. Cell lysates were subjected to western blotting for HIF1A, FLAG and ACTB. **(f)** A luciferase reporter under control of multiple HIF1α binding sites (HRE) was transfected into cells with EV or RASSF1C-FLAG. 6 hr after transfection, cells were exposed to Hypoxia for 24 hr. Cells were lysed and luciferase activity was measured and normalized to co-transfected Renilla luciferase internal control. $^{***}P < 0.001$ compared to EV (Hypoxia), one-way ANOVA followed by SNK multiple comparison test. Data represent mean ± s.e.m. n = 3 independent experiments.

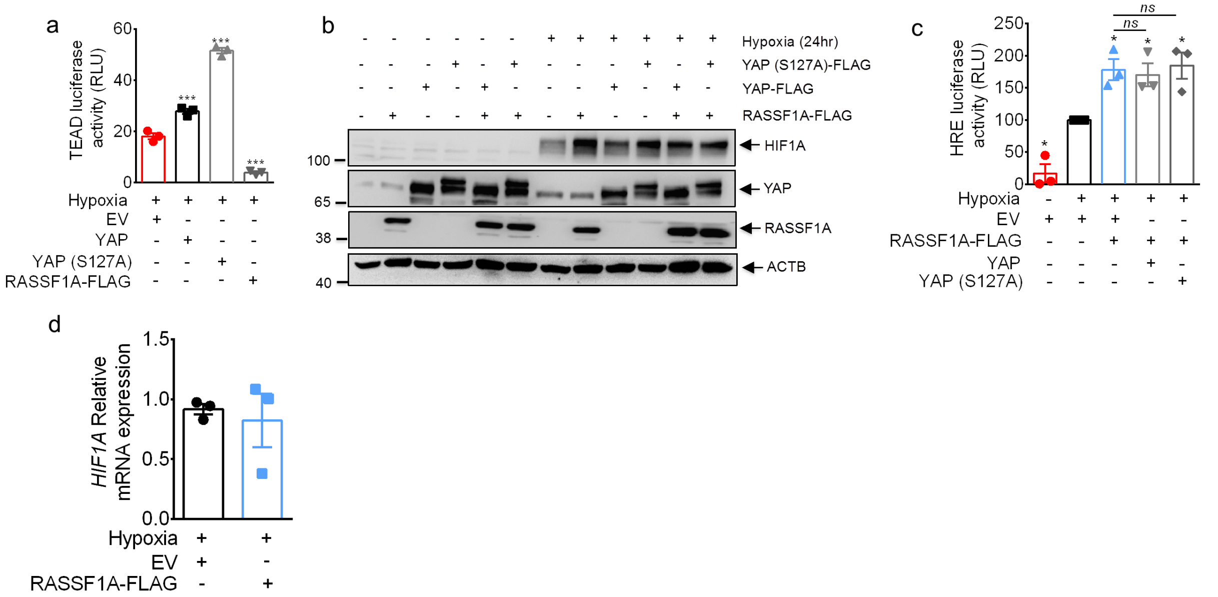

Supplementary Fig. 7: RASSF1A regulates HIF1α, independent of hippo signaling. A luciferase reporter under control of **(a)** TEAD binding sites or **(c)** of multiple HIF1 α binding sites (HRE) was transfected into HeLa cells with EV, YAP, YAP (S127A) and RASSF1A-FLAG. 6 hr after transfection, cells were exposed to Hypoxia for 24 hr. Cells were lysed and luciferase activity was measured and normalized to co-transfected Renilla luciferase internal control. ***$P < 0.001$ compared to EV (hypoxia), one-way ANOVA followed by SNK multiple comparison test. **(b)** HEK 293 cells were transfected with plasmids indicated on top of lanes and exposed to hypoxia for 24 hr, followed by western blotting for HIF1A, YAP, RASSF1A and ACTB. **(d)** Effect of RASSF1A overexpression on mRNA expression of HIF1A in HEK293 cells exposed to 24 hr Hypoxia. Data represent mean ± s.e.m. n = 3 independent experiments.

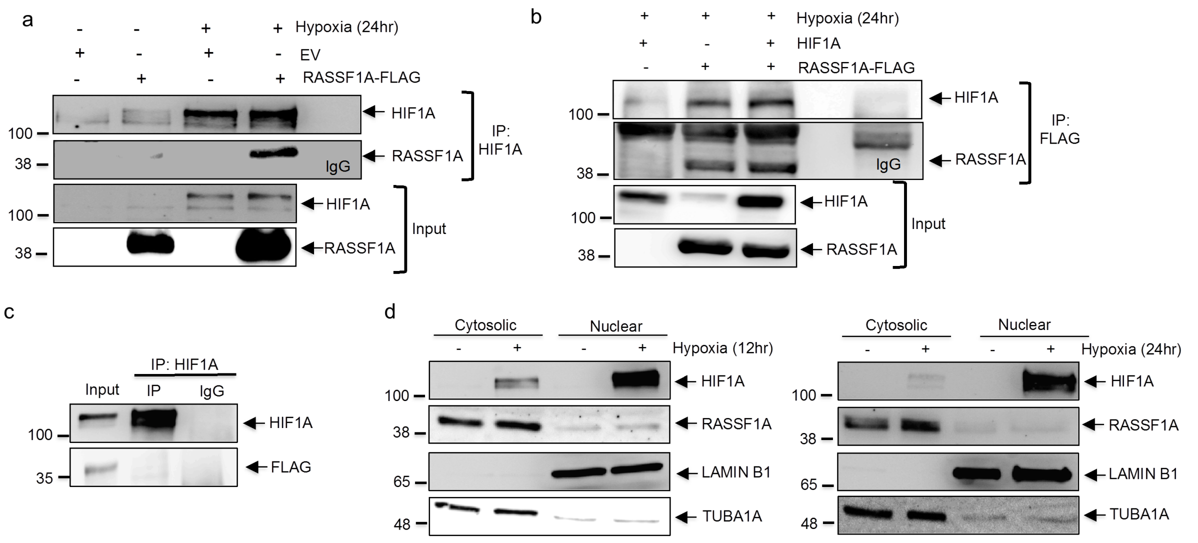

Supplementary Fig. 8: RASSF1A interacts with HIF1α. (a, b) HEK293 cells were transfected with plasmids indicated on top of lanes and exposed to Hypoxia for 24hr. **(a)** HIF1A or **(b)** FLAG was immunoprecipitated (IP) and co- immunprecipitated (co-IP) RASSF1A or HIF1A were detected by western blotting. **(c)** HEK 293 cells were transfected with RASSF1C-FLAG plasmid and exposed to hypoxia for 24 hr, followed by HIF1A IP and western blotting for HIF1A and FLAG. **(d)** Human PASMCs were exposed to Hypoxia or Normoxia for 12 hr and 24 hr, followed by sub cellular fractionation. Cytoplasmic and nuclear lysates were subjected to western blotting for HIF1A, RASSF1A, Lamin B1 and TUBA1A. Lamin B1 and alpha-tubulin (TUBA1A) were used as nuclear and cytoplasmic markers respectively. n = 2 independent experiments. n = 2 independent experiments**.**

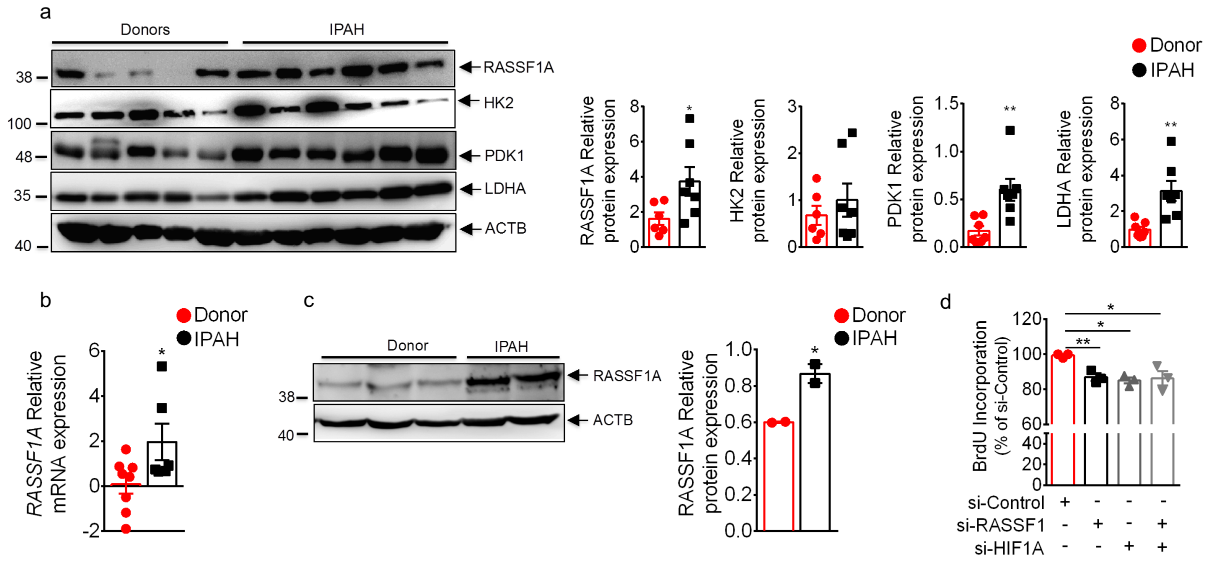

Supplementary Fig. 9: Protein expression of RASSF1A and glycolytic enzymes is upregulated in IPAH patients. (a) Protein was isolated from frozen pulmonary arteries of IPAH patients (n=6) and donors (n=5) and subjected to **(a, left)** western blotting for RASSF1A, HK2, PDK1, LDHA and ACTB, followed by **(a, right)** densitometric quantification of RASSF1A, HK2, PDK1 and LDHA to ACTB expression ratio. **(b, c)** Expression of RASSF1A in IPAH- vs donor- PAAFs (n= 2-5) as analyzed using **(b)** real time PCRs and **(c)** western blotting for RASSF1A and ACTB followed by densitometric quantification of relative RASSF1A expression. *$P < 0.05$, **$P < 0.01$ compared to donor, unpaired Student's *t*-test. **(d)** IPAH PASMCs were transfected with RASSF1 siRNA (si-RASSF1), HIF-1α siRNA (si-HIF1A) or in combination. 6 hr after transfection, cells were placed in medium with growth factors for 48 hr. Proliferation was measured by BrdU incorporation assay. **$P < 0.01$, ***$P < 0.001$ compared to si-Control, one-way ANOVA followed by SNK multiple comparison test. Data represent mean ± s.e.m. n = 3 independent experiments from 3 biological replicates for IPAH PASMCs.

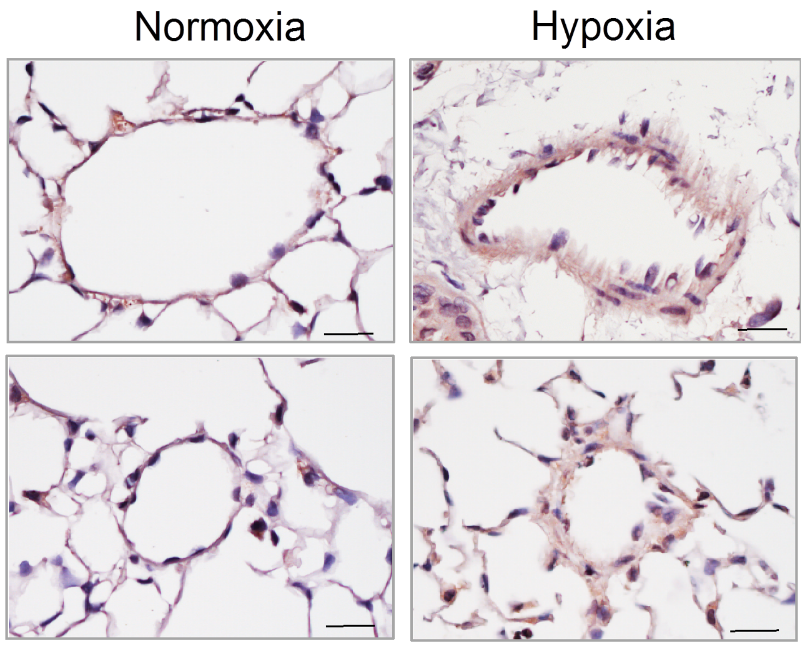

Supplementary Fig. 10: RASSF1A is upregulated in hypoxic mice lungs. Representative immunostaining microphotographs of mice lung sections from normoxic (n=3) and 3-week hypoxic mice (n=3), stained for RASSF1A (brown color). Scale bar: 20 µm.

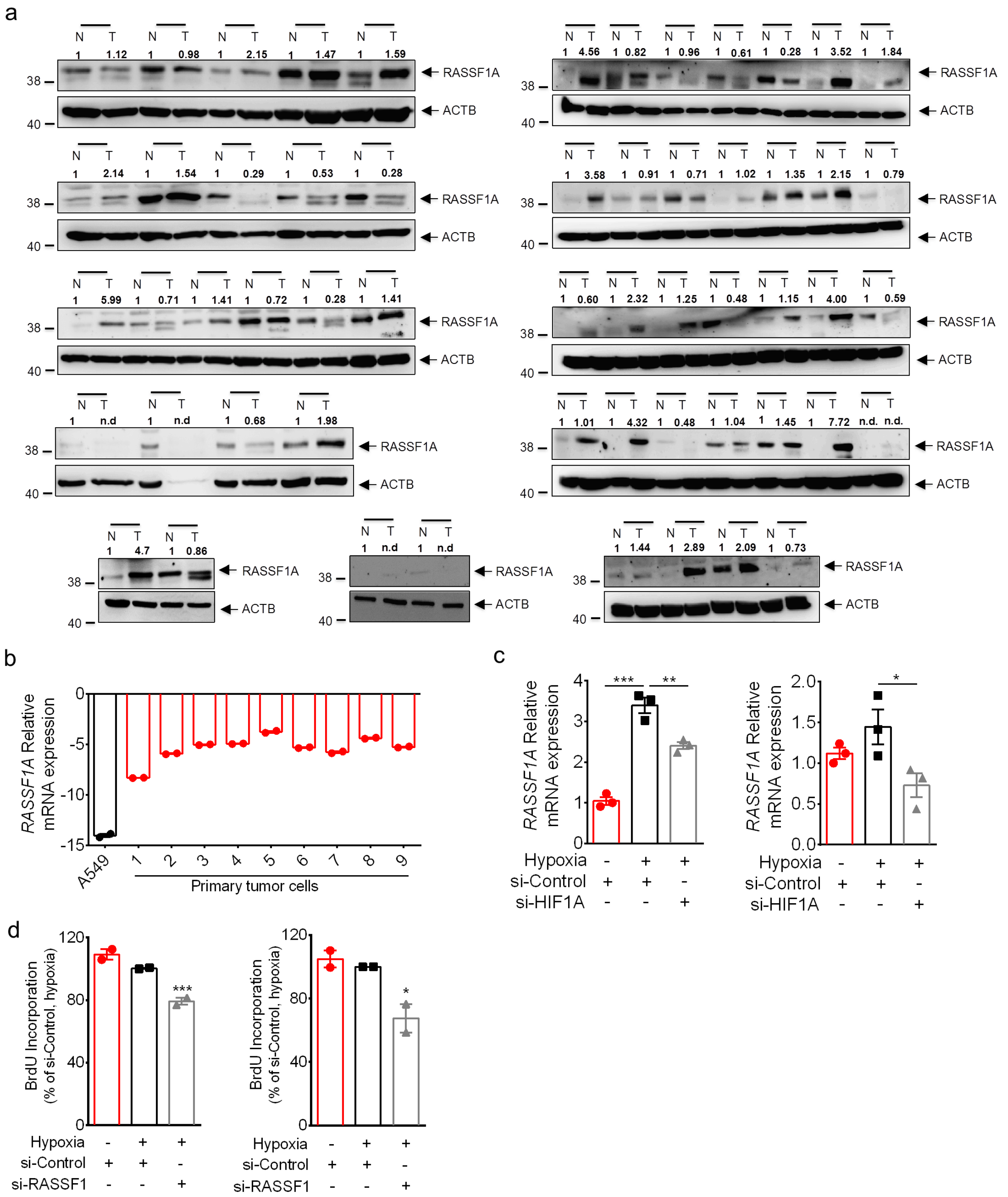

Supplementary Fig. 11: RASSF1A- HIF-1α axis in primary lung tumor cells. (a) Proteins were isolated from tumor (T) and non-tumor (N) areas of human non-small cell lung cancer lungs (n= 57) followed by western blotting for RASSF1A, followed by densitometric analysis. Non-tumor sample values were taken as 1 and expression of RASSF1A in corresponding tumor was calculated as a fold change. **(b)** RASSF1A mRNA expression in different primary tumor cells as analyzed by real time PCRs. **(c)** two different primary tumor cells were transfected with siHIF1α (si-HIF1A) or control siRNA (si-Control). 24hr after transfection, cells were exposed to Normoxia or Hypoxia for 24hr, followed by expression analysis of RASSF1A by real time PCRs. **(d)** Primary lung cancer cells were transfected with RASSF1 siRNA (si-RASSF1) or control siRNA (si-control). 24 hr after transfection, cells were placed in hypoxia for 48 hr. Proliferation was measured by BrdU incorporation assay. $^{**}P < 0.01$, $^{***}P < 0.001$ compared

to si-Control (Hypoxia), one-way ANOVA followed by SNK multiple comparison test. Data represent mean ± s.e.m all through the figure. For primary tumor cells, n=2 independent experiments from 2 biological replicates (represented as separate).

Fig 1e

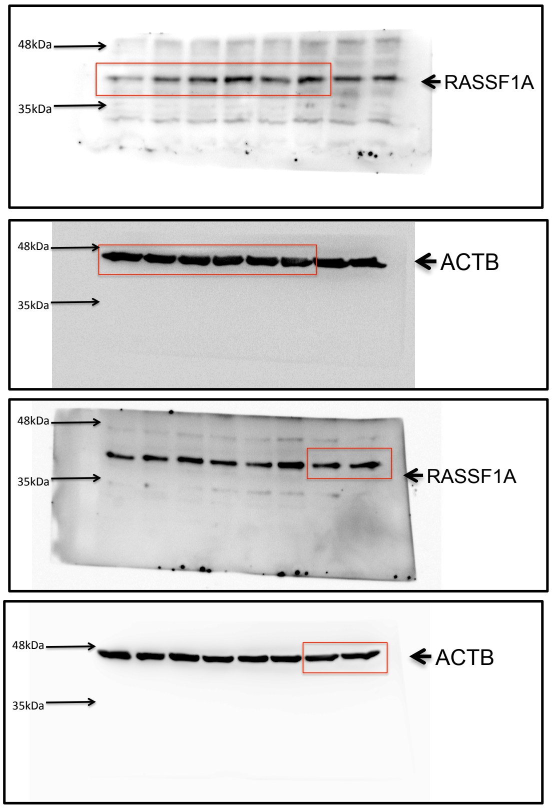

Fig 1e-g

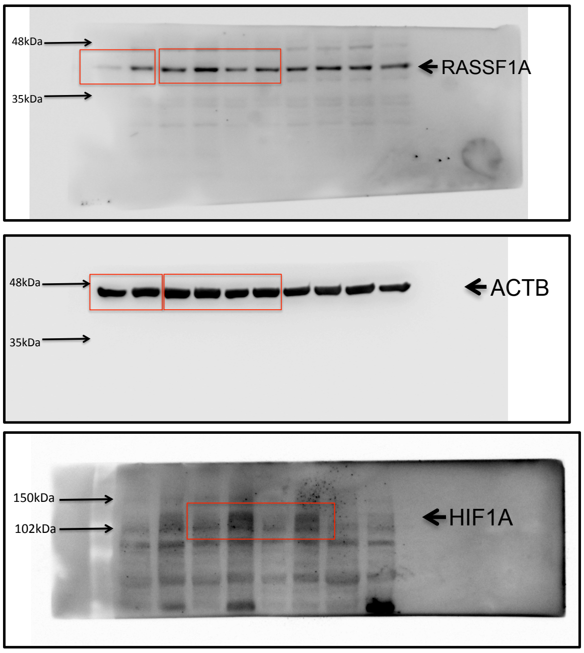

Supplementary Fig. 12: Uncropped scans of western blots for Fig. 1.

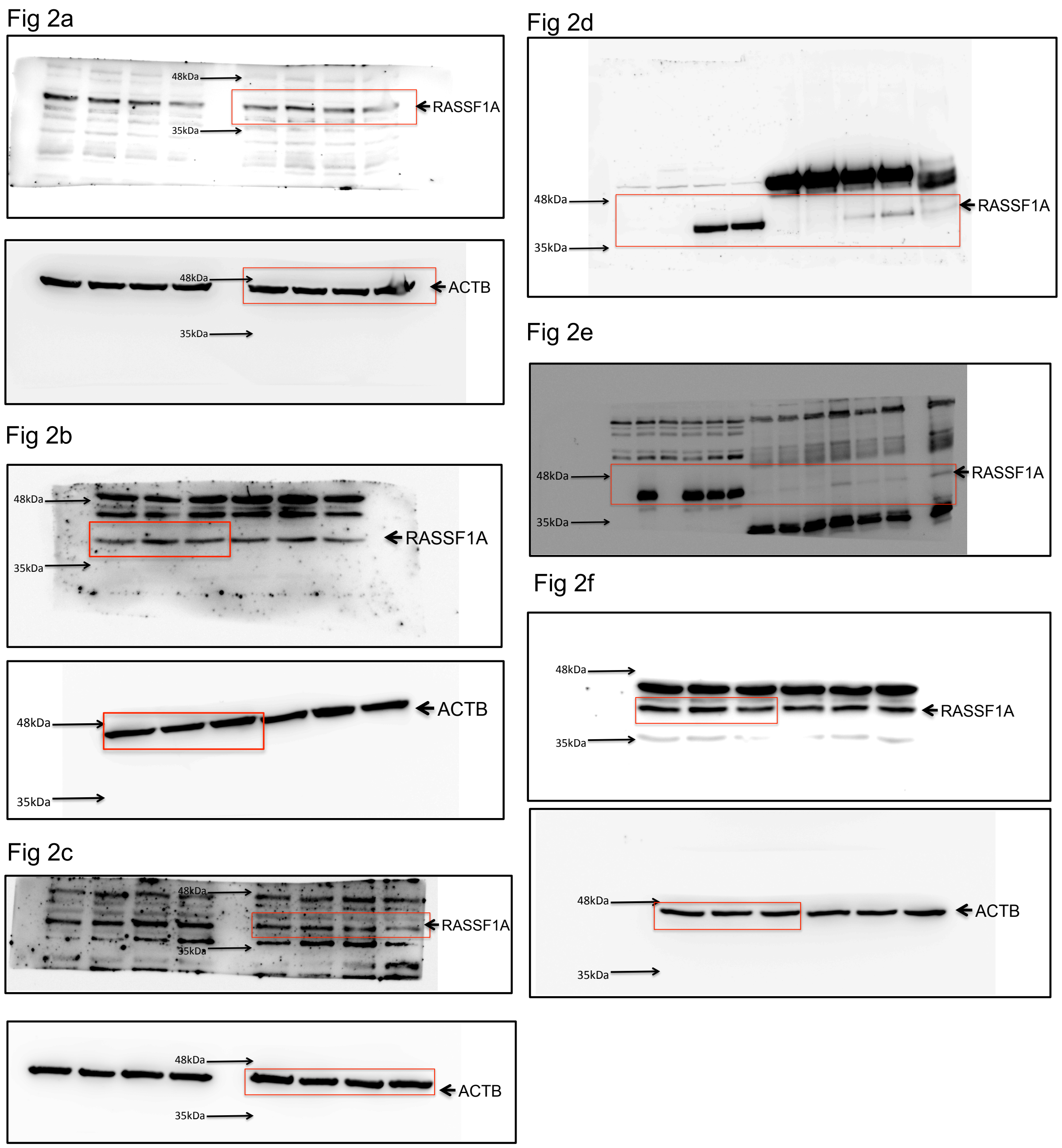

Supplementary Fig. 13: Uncropped scans of western blots for Fig. 2a – 2f.

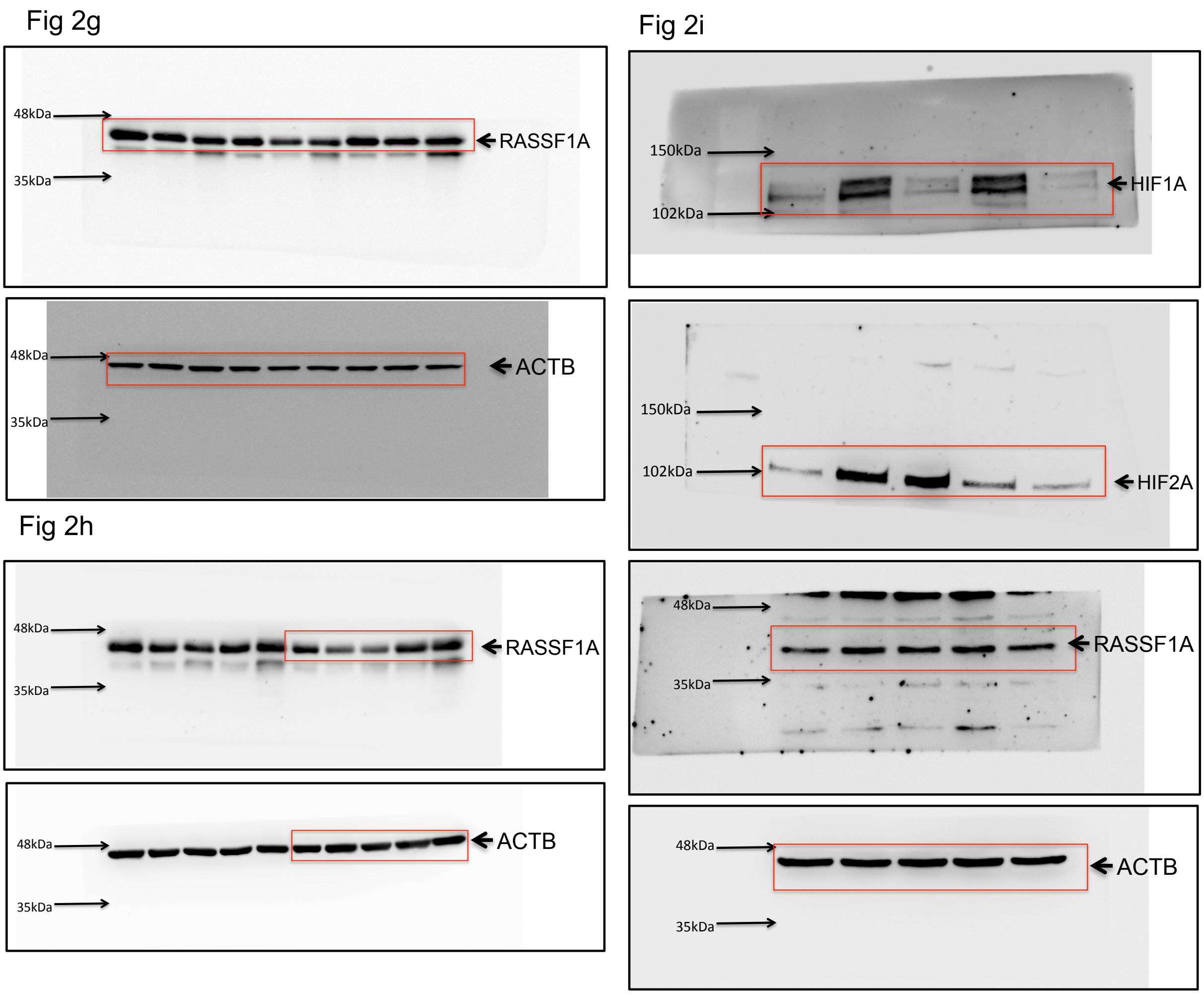

Supplementary Fig. 14: Uncropped scans of western blots for Fig. 2g – 2i.

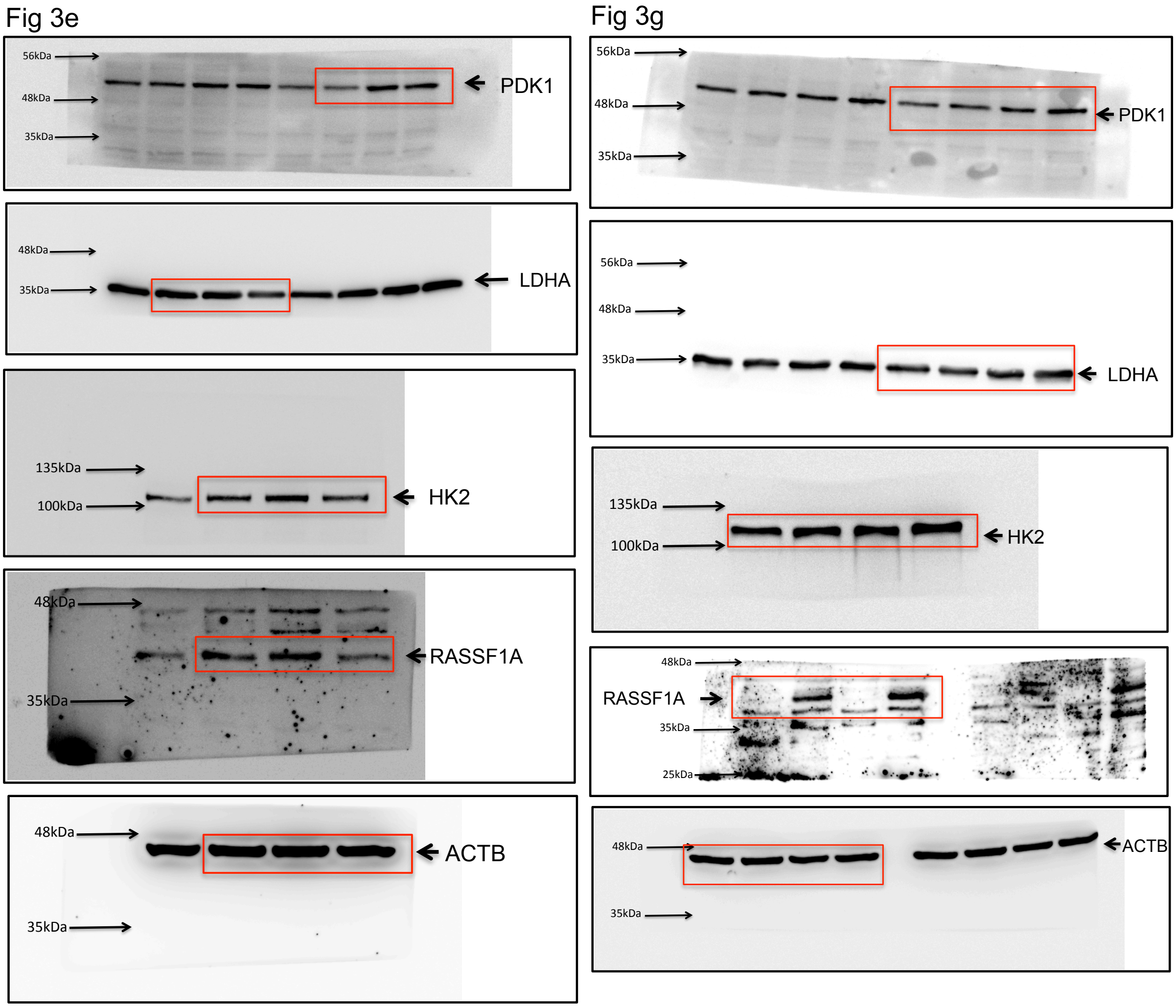

Supplementary Fig. 15: Uncropped scans of western blots for Fig. 3e and 3g.

Fig 4a

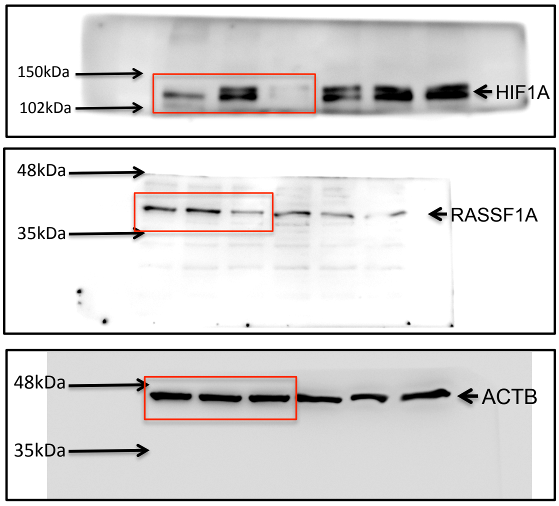

Fig 4e

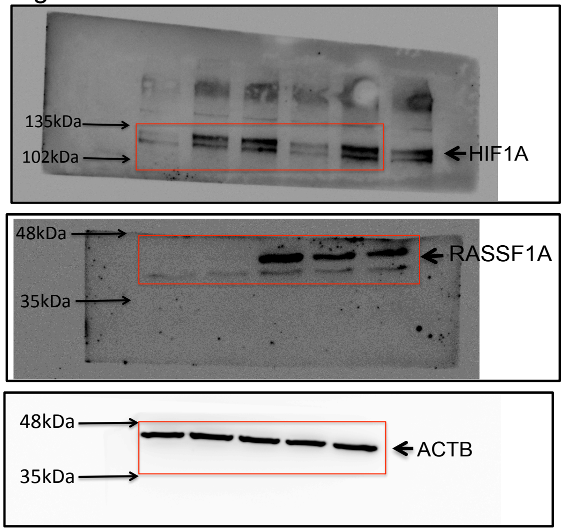

Fig 4b

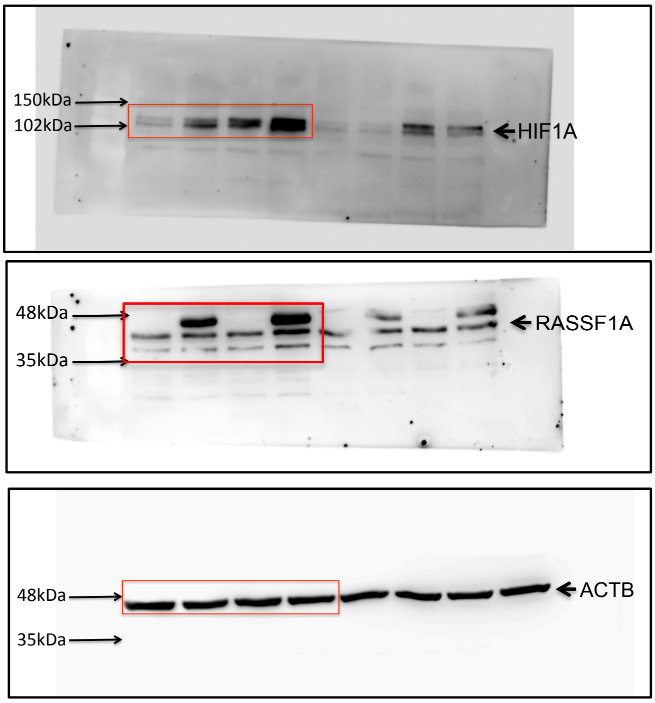

Supplementary Fig. 16: Uncropped scans of western blots for Fig. 4a, 4b and 4e.

Fig 5a

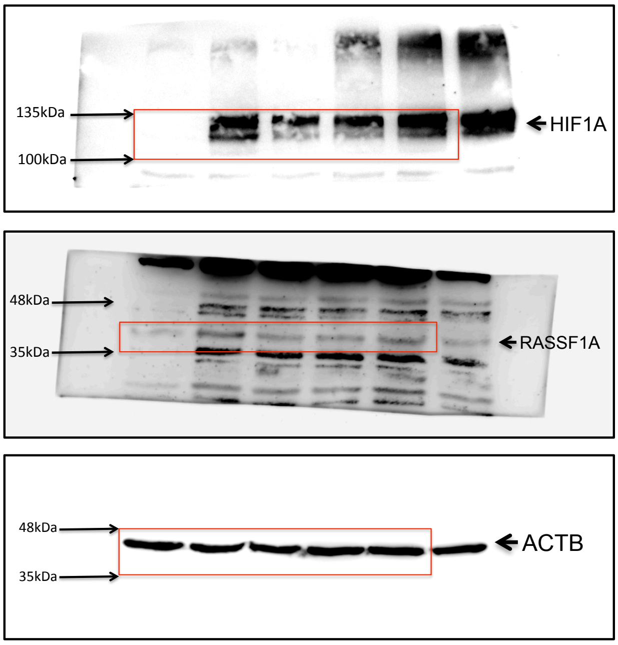
135kDa
100kDa
HIF1A
48kDa
35kDa
RASSF1A
48kDa
35kDa
ACTB

Fig 5b

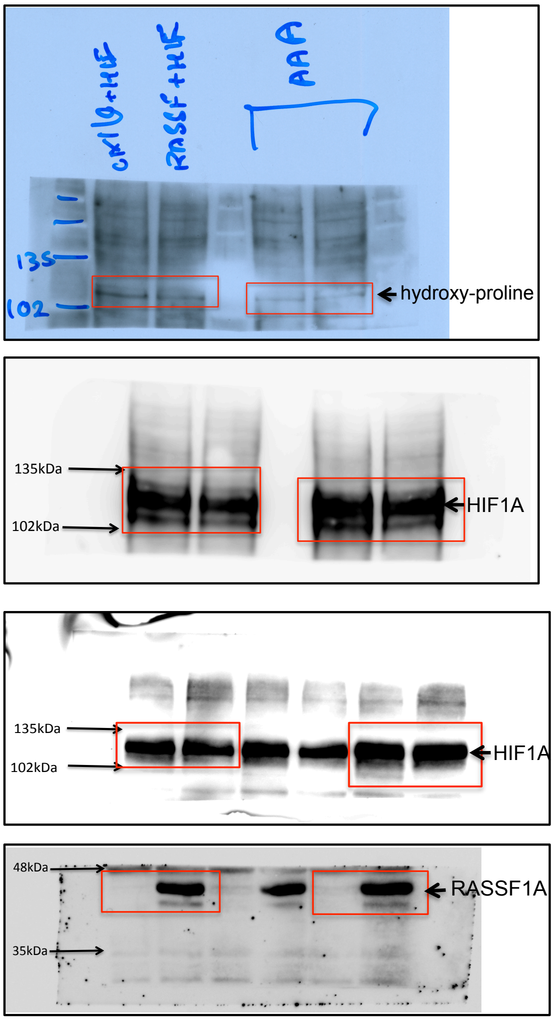
135
102
hydroxy-proline
135kDa
102kDa
HIF1A
135kDa
102kDa
HIF1A
48kDa
35kDa
RASSF1A

Fig 5c

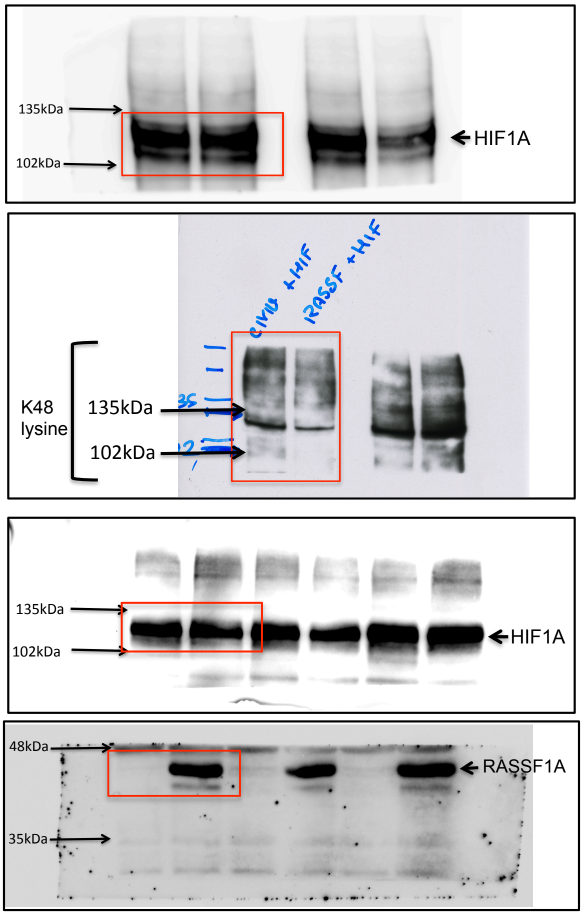

Fig 5d

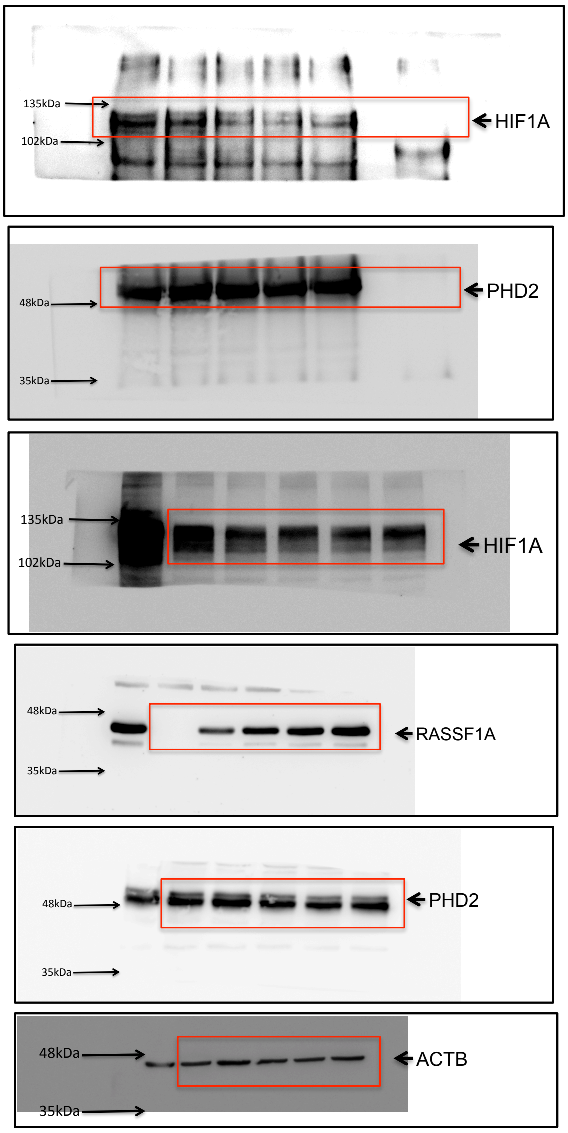

Fig 5e

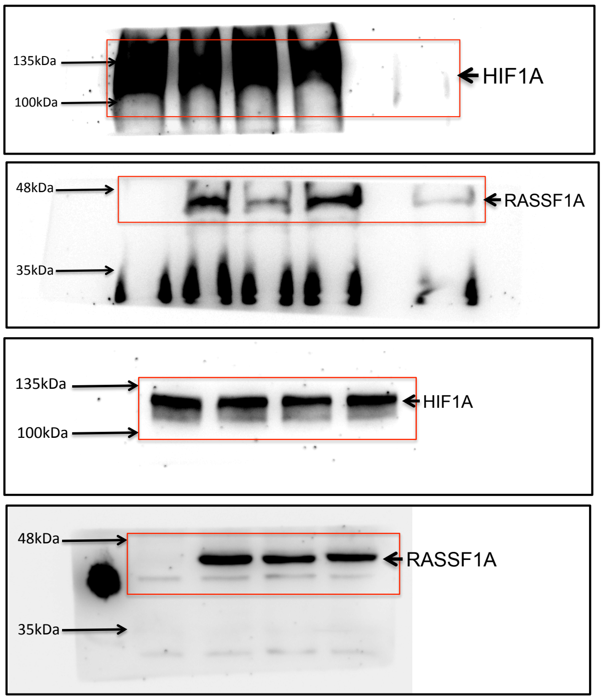

Fig 5f

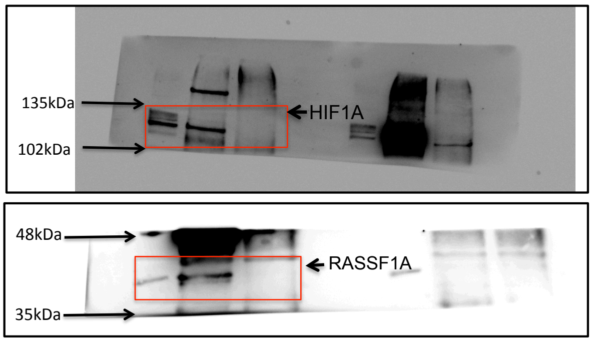

Supplementary Fig. 17: Uncropped scans of western blots for Fig. 5a – 5f.

Fig 6e

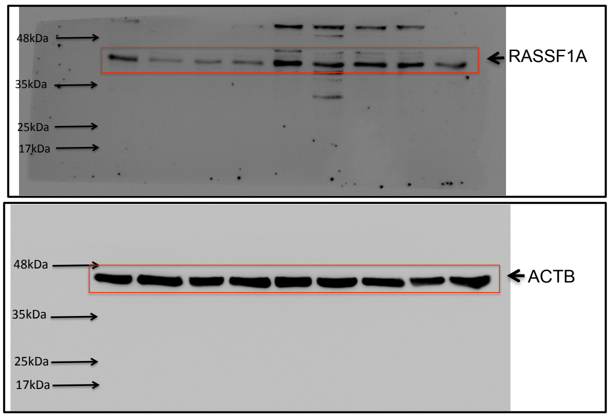

Fig 6h

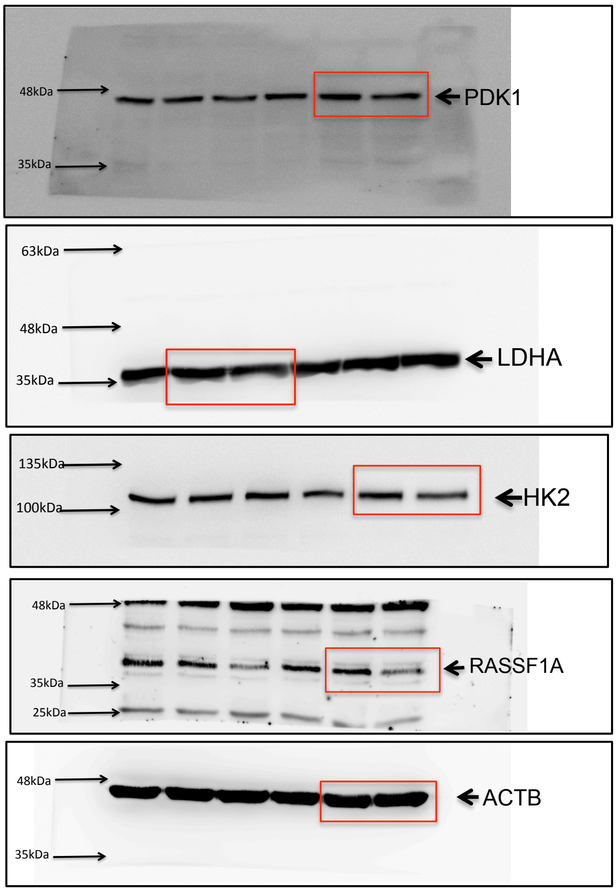

Supplementary Fig. 18: Uncropped scans of western blots for Fig. 6e and 6h.

Fig 8a

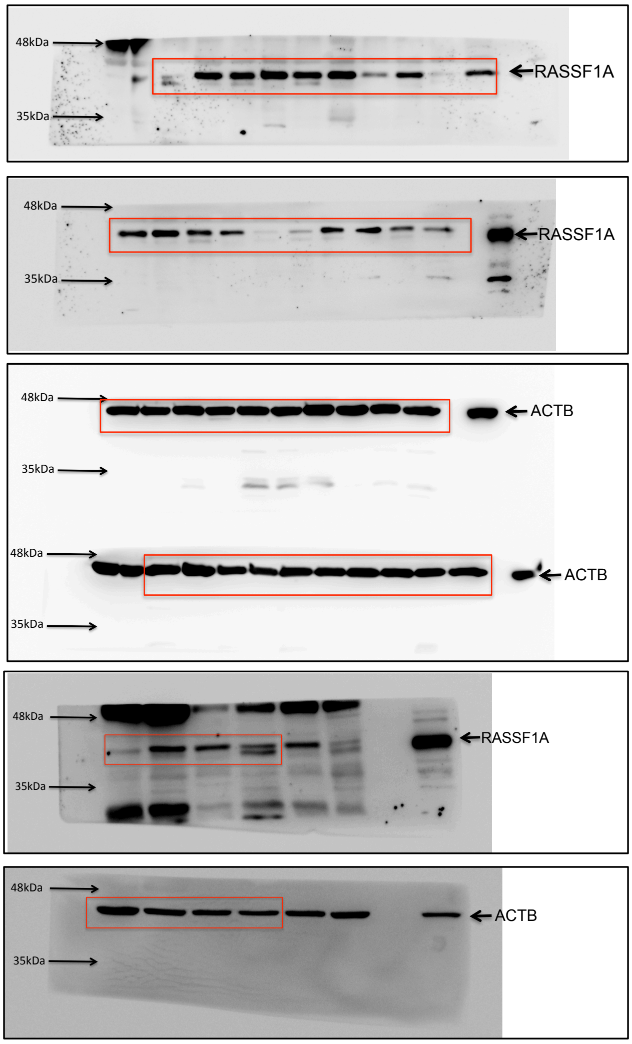

Fig 8c

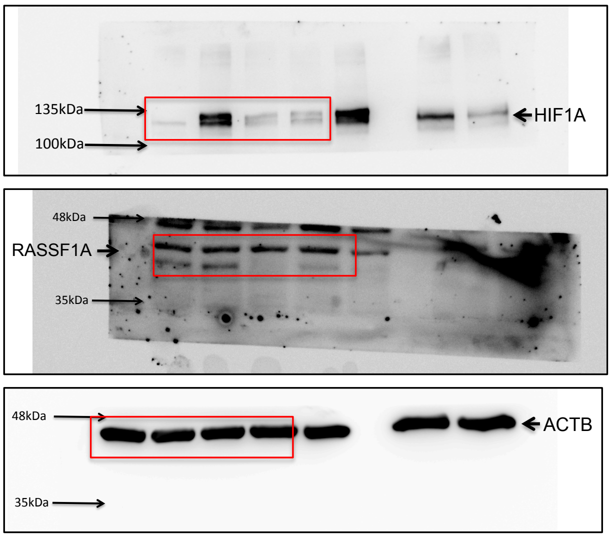

Fig 8e

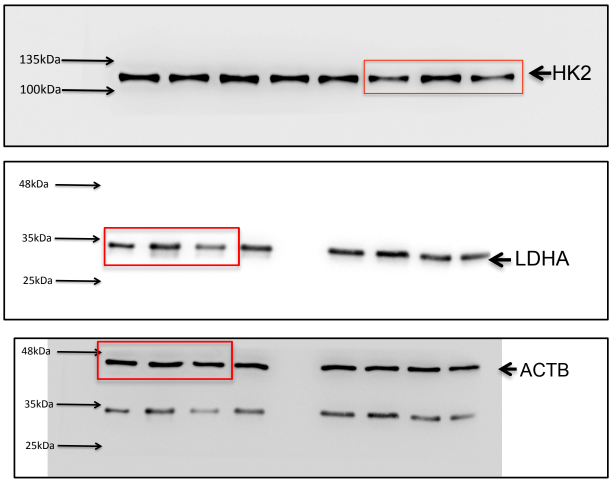

Fig 8g

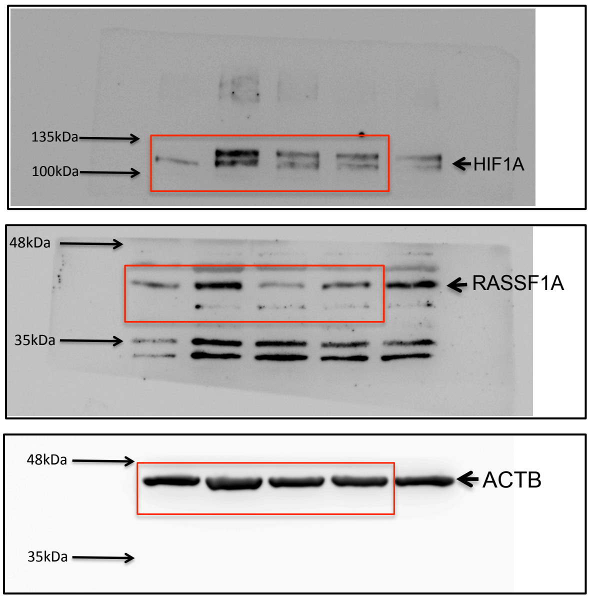

Fig 8i

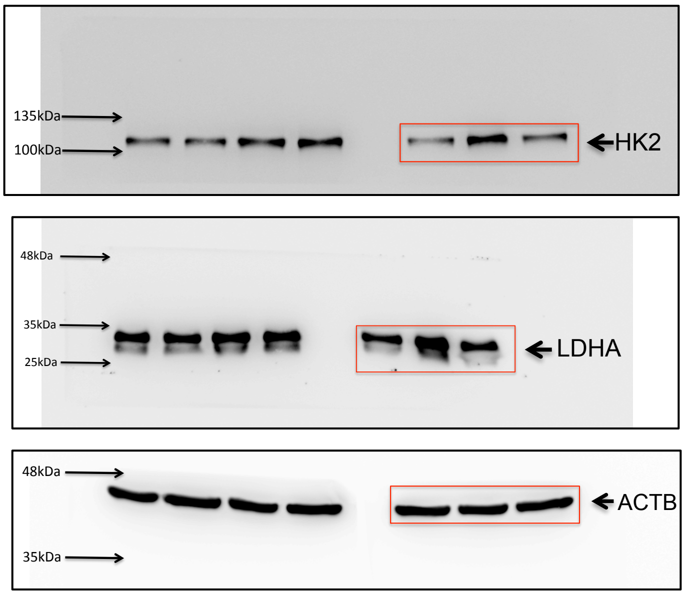

Supplementary Fig. 19: Uncropped scans of western blots for Fig. 8a, 8c, 8e, 8g, 8i.

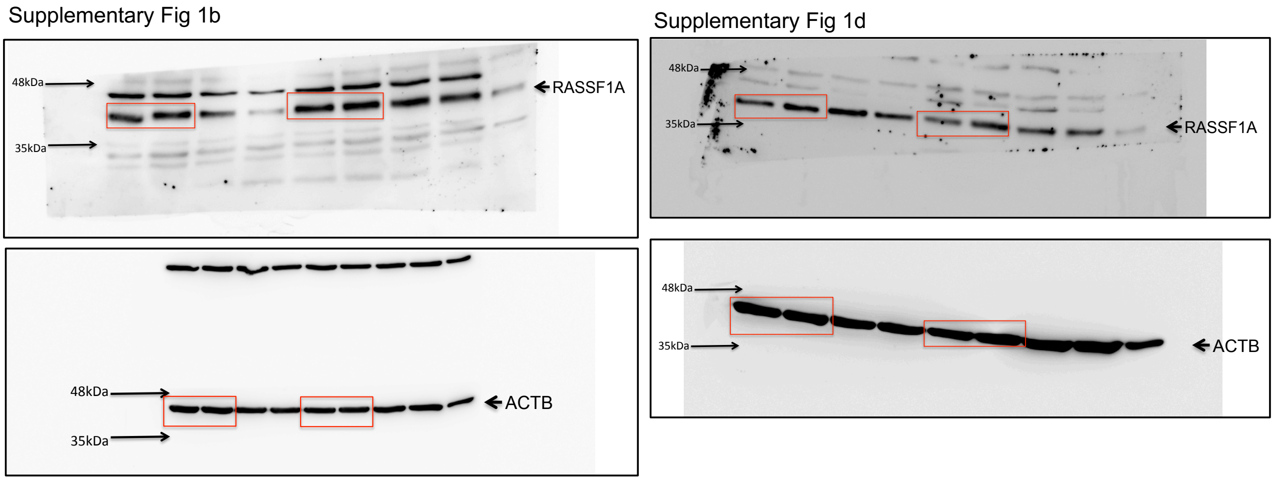

Supplementary Fig. 20: Uncropped scans of western blots for Supplementary Fig. 1b and 1d.

Supplementary Fig 2a

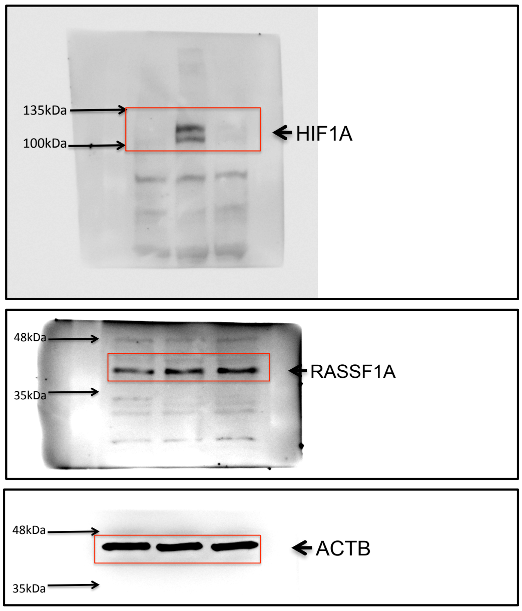

Supplementary Fig 2b

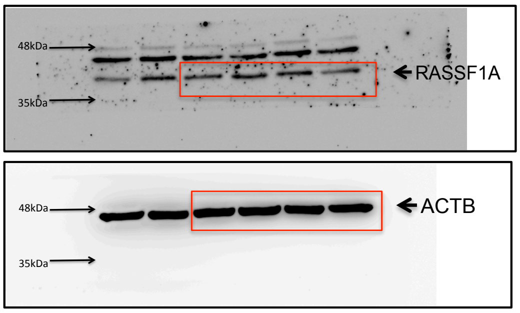

Supplementary Fig 2c

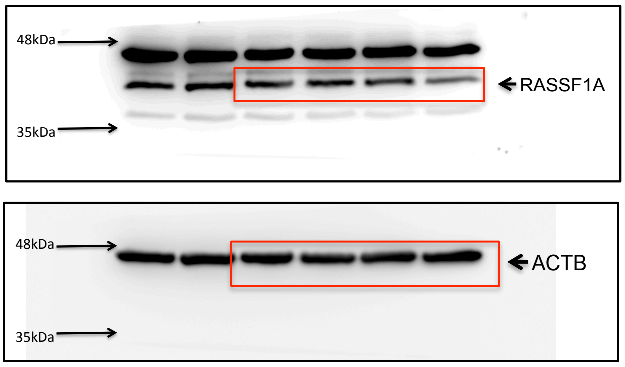

Supplementary Fig 2e

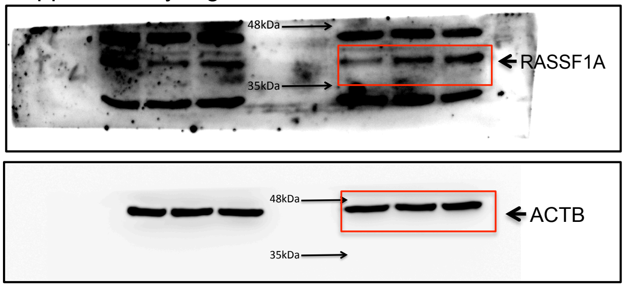

Supplementary Fig 2f

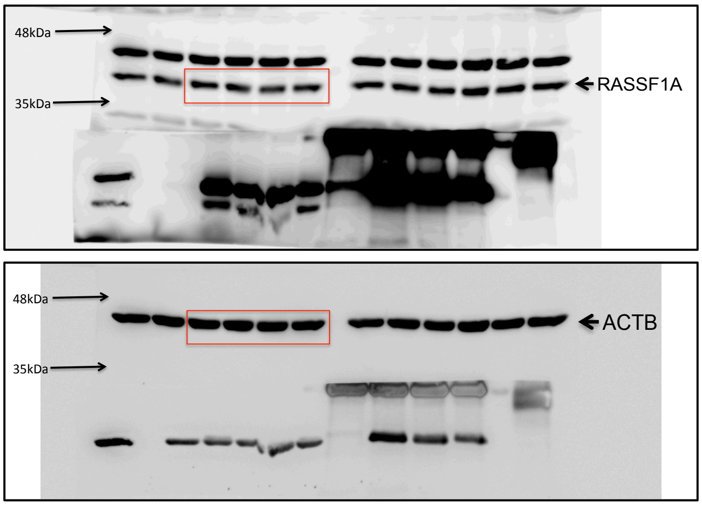

Supplementary Fig 2g

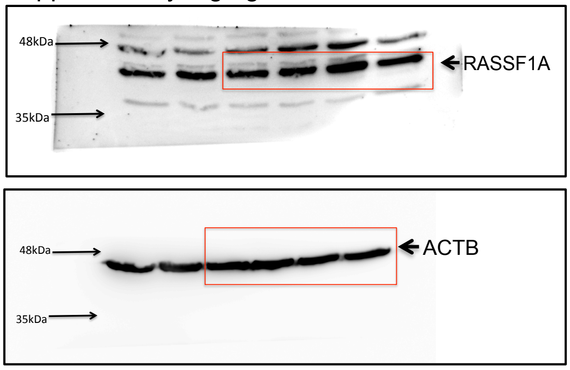

Supplementary Fig 2i

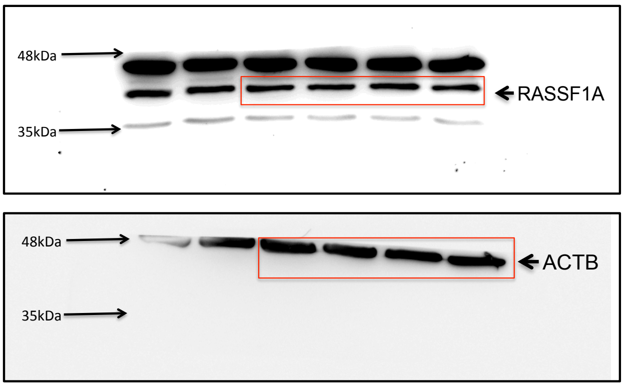

Supplementary Fig 2h

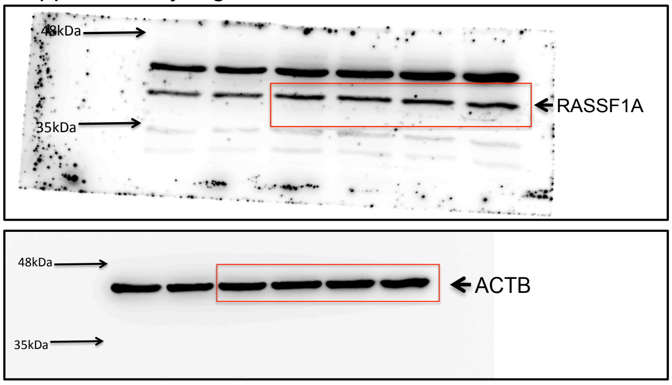

Supplementary Fig 2j

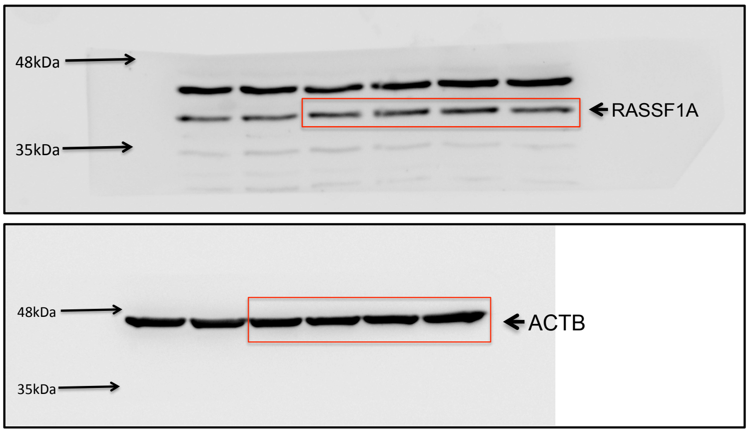

Supplementary Fig. 21: Uncropped scans of western blots for Supplementary Fig. 2a -2j.

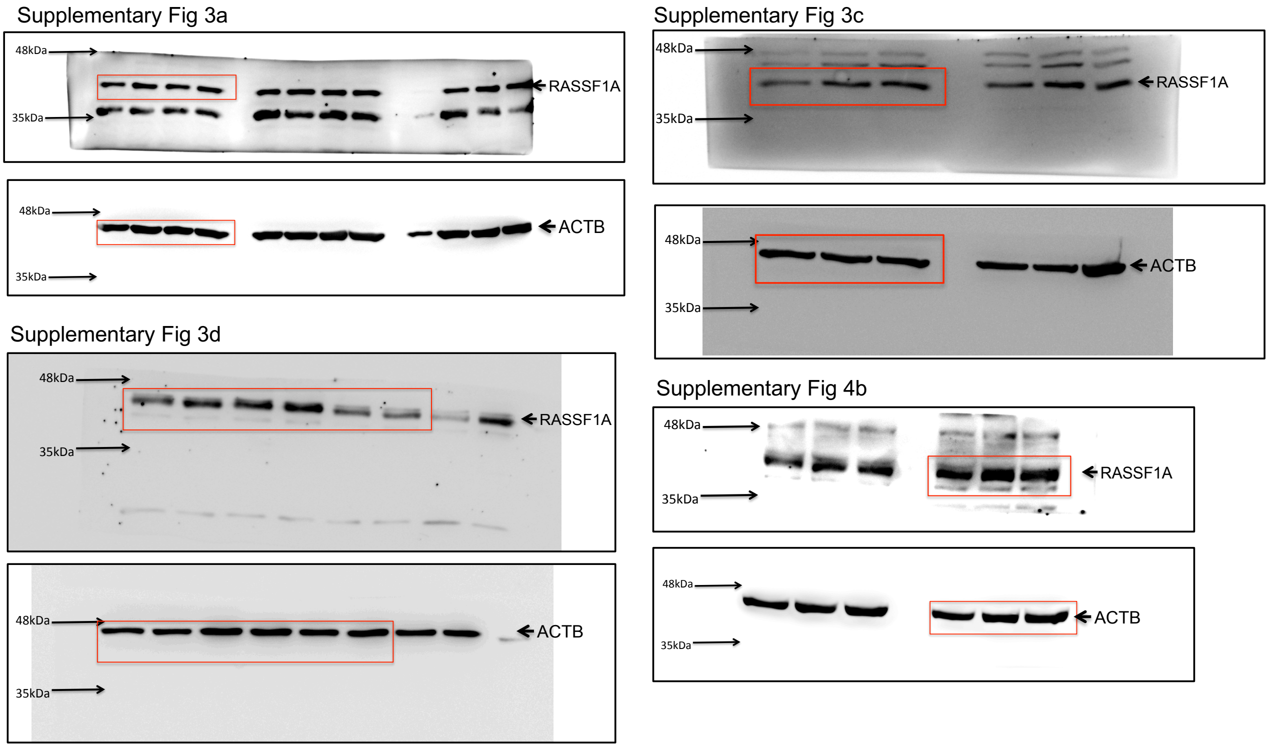

Supplementary Fig. 22: Uncropped scans of western blots for Supplementary Fig. 3a, 3c, 3d and 4b.

Supplementary Fig 6a

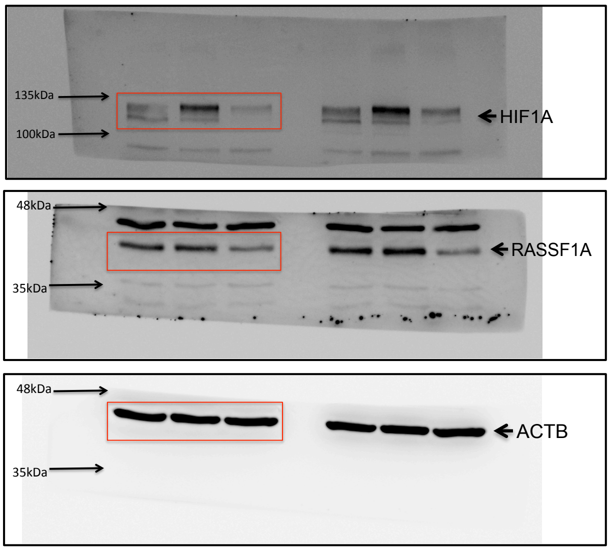

Supplementary Fig 6b

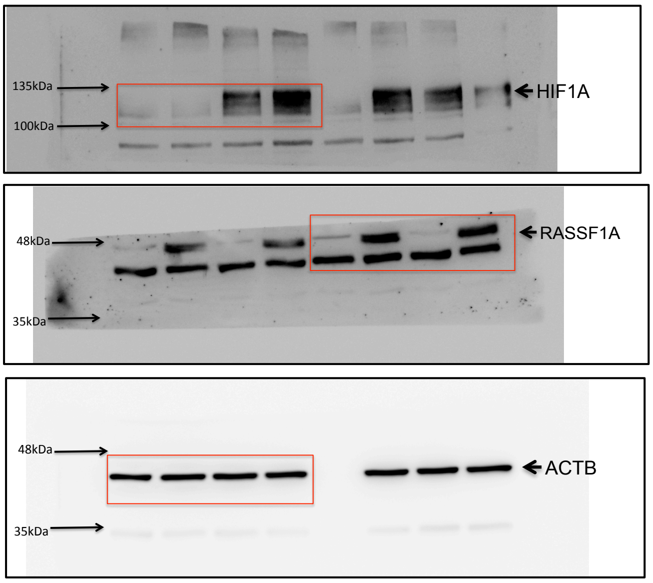

Supplementary Fig 6c

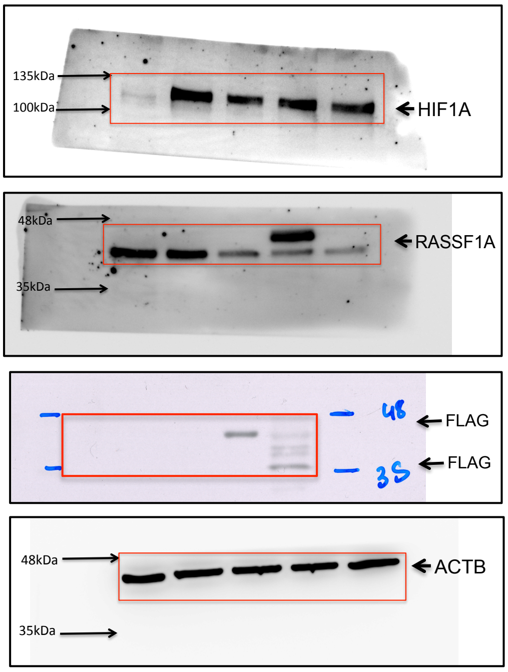

Supplementary Fig 6e

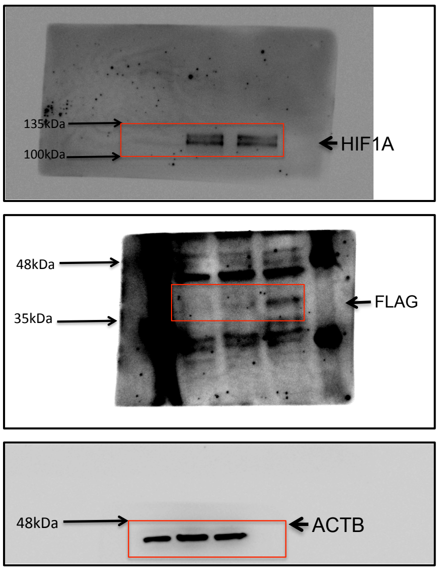

Supplementary Fig. 23: Uncropped scans of western blots for Supplementary Fig. 6a – 6c and 6e.

Supplementary Fig 7b

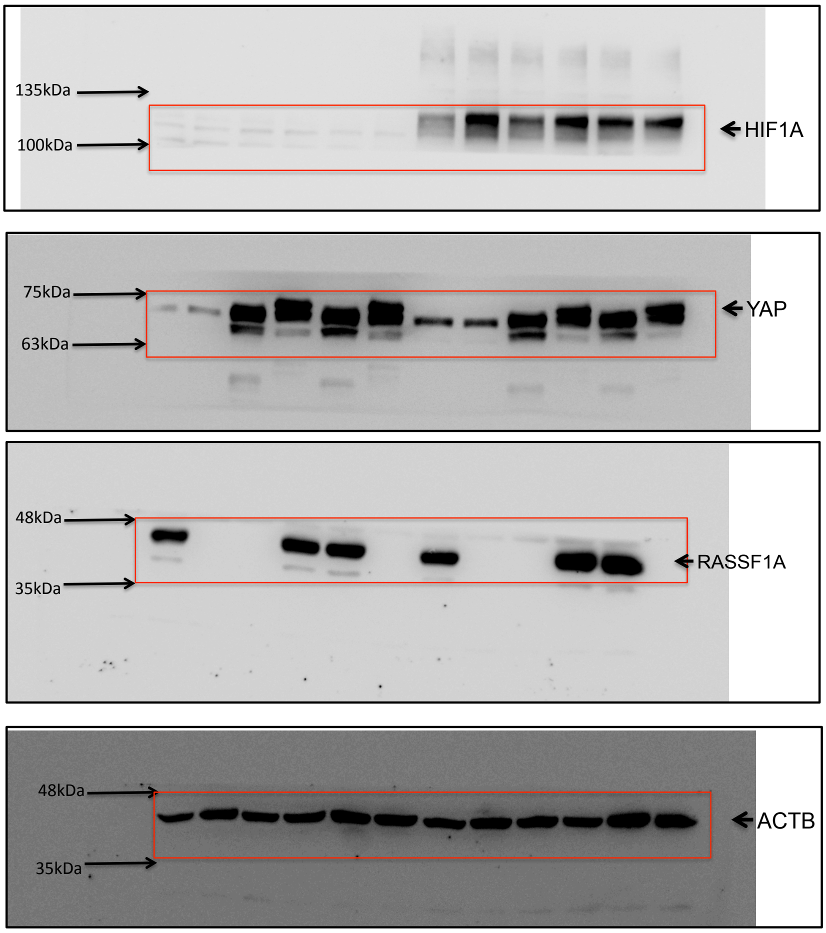

Supplementary Fig. 24: Uncropped scans of western blots for Supplementary Fig. 7b.

Supplementary Fig 8a

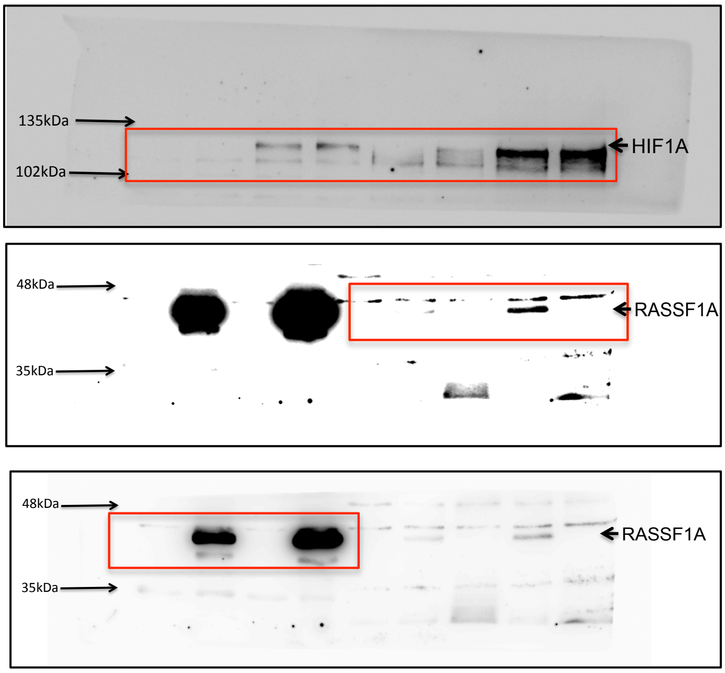

Supplementary Fig 8c

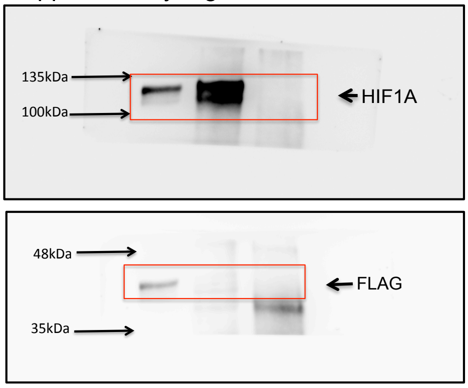

Supplementary Fig 8b

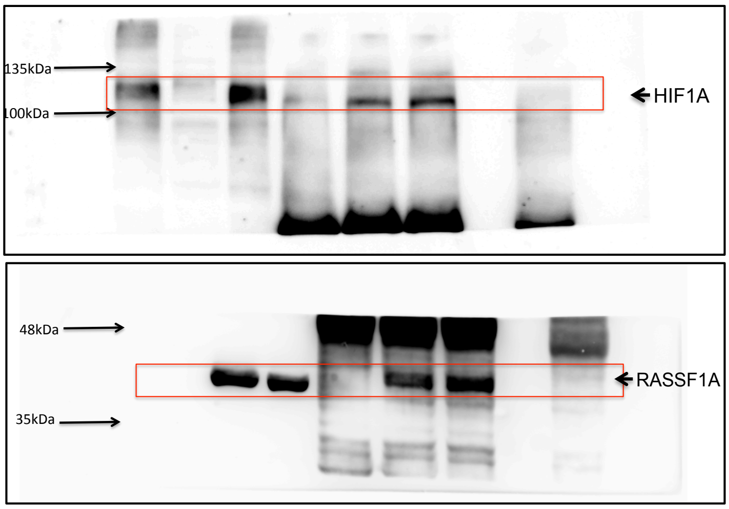

Supplementary Fig 8d

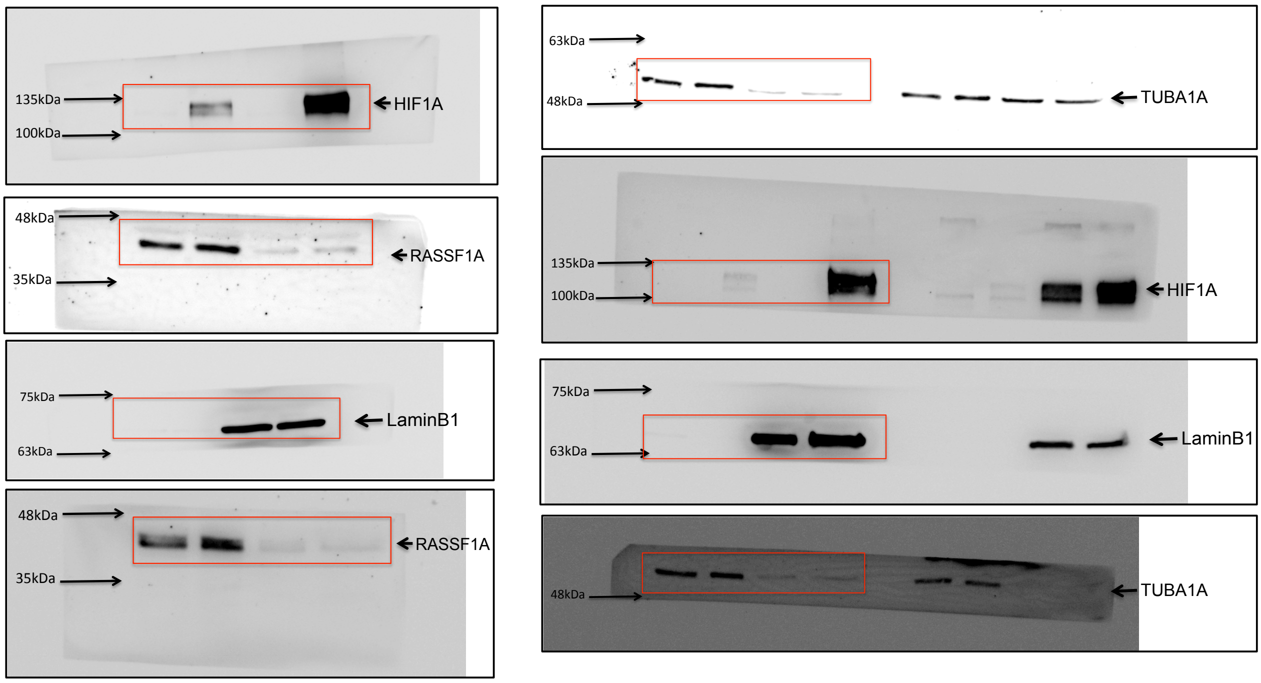

Supplementary Fig. 25: Uncropped scans of western blots for Supplementary Fig. 8a-8d.

Supplementary Fig 9a

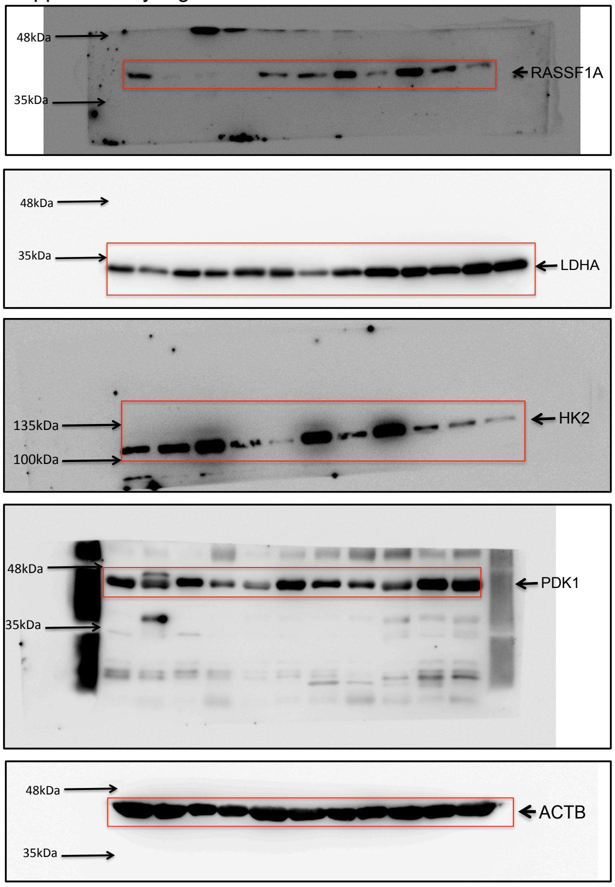

Supplementary Fig 9c

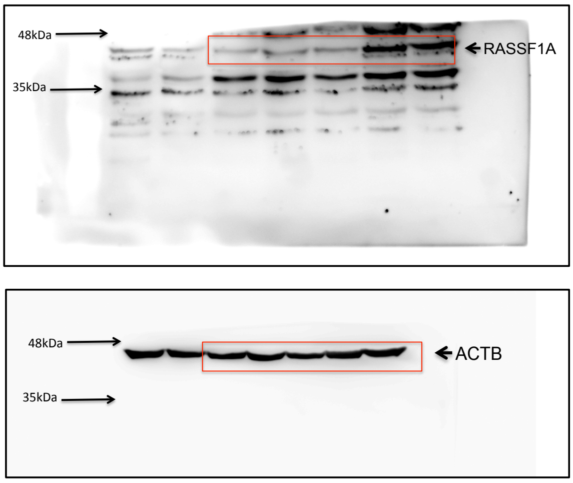

Supplementary Fig. 26: Uncropped scans of western blots for Supplementary Fig. 9a and 9c.

Supplementary Fig 11a

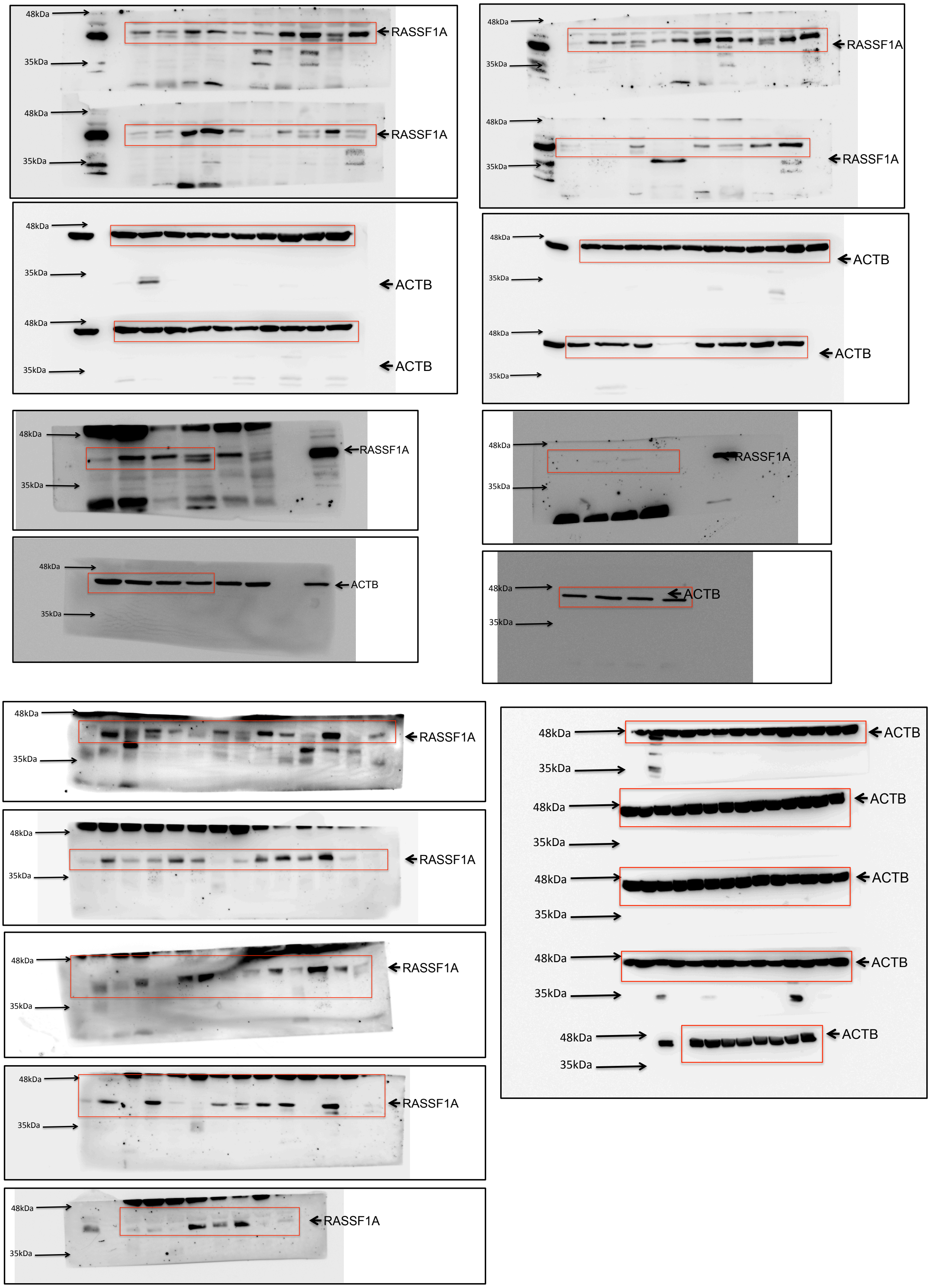

Supplementary Fig. 27: Uncropped scans of western blots for Supplementary Fig. 11a.

Supplementary Table 1: Real time PCR Primers

	Species	Forward Primer	Reverse Primer
**RASSF1A**	Human	GGCTGGGAACCCGCGGTG	TCCTTGCAAGGAGGGTGGCTTCT
**HIF1A**	Human	TAAAGGAATTTCAATATTTGATGGG	AAAGGGTAAAGAACAAAACACACAG
**HIF2A**	Human	GATCTTTCTGTCAGGAAACATCAGC	GTTGACAGTACGGCCTCTGTTG
**PDK1**	Human	CTCAGGACACCATCCGTTCA	ATCTTGCAGGCCATACAGCA
**LDHA**	Human	GAAGATAAGTGGTTTTCCCAAAAA	CTTTGAGTTTGATCACCTCATAAGC
**HK2**	Human	GCAGGATGATTGCCTCGC	ACTCTCCGTGTTCTGTCC
**CA9**	Human	TTTGCCAGAGTTGACGAGGC	CTGAGCCTTCCTCAGCGATT
**PRKCA**	Human	GCCTATGGCGTCCTGTTGTA	CTCCTTTGCCACACACTTTGG
**PRKCB**	Human	CACTCCAGACTACATCGCCC	TCTCTTGTCTCTAGCTTTTGGCT
**NOX1**	Human	TGTTTGTGGATGCCTTCCTG	AAGGACAGCAGATTGCGACA
**B2M**	Human	AGATGAGTATGCCTGCCG	TCATCCAATCCAAATGCG
**HPRT**	Human	TGACACTGGCAAAACAAT	GGTCCTTTTCACCAGCAA
**RASSF1A**	Mouse	GCGACCTCTGTGGAGACTTC	CTCTAGTGCAGAGTCCCAGC
**18s rRNA**	Mouse	GTAACCCGTTGAACCCCATT	CCATCCAATCGGTAGTAGCG
**RASSF1A**	Rat	CACAACACGCAATCCGTCAC	CGACATCGGTAATGGCAGGT
**HPRT**	Rat	GACTTTGCTTTCCTTGGTCA	AGTCAAGGGCATATCCAACA
**RASSF1A_HRE1_CHIP**	Human	CACAGTAAAGCTGGCCTCCA	CTCCTTCGTCCCCTCCTCA
**RASSF1A_HRE2_CHIP**	Human	TGCAGGTGGGTTGAGGTTAC	GAGGGCCTTCAGTGCAATCT
**LDHA_CHIP**	Human	ACTCAGGCTCATGGCTC	GGCTGGGGGTGGATG
**PDK1_CHIP**	Human	CGCGTTTGGATTCCGTG	CCAGTTATAATCTGCCTTCCCTATTATC
**HK2_CHIP**	Human	GCCCCGCAGGTAGTCAG	AGCCACGATTCTCTCCACG

Supplementary Table 2: siRNA target sequences

Gene	Target Sequence
**RASSF1**	AAGCACCGAAGCGAAACTTAA
**HIF1A**	AACCAAGTAGCCTGTTATCAA
**HIF2A**	CCCGGATAGACTTATTGCCAA
**PRKCA**	CGCAGTGGAATGAGTCCTTTA
**PRKCB**	CCGGATGAAACTGACCGATTT
**NOX1**	CTGAATCTTCCCTGTTGCCTA
**Control siRNA**	AATTCTCCGAACGTGTCACGT

Supplementary Table 3: Antibodies

Antibody	Company	Application
**RASSF1A**	Abcam (ab23950)	Western blotting
**RASSF1**	Santa Cruz (sc-18722)	Immunohistochemistry
**RASSF1A**	Biorbyt (orb11328)	Proximity ligation assay
**RASSF1A**	Abcam (ab91212)	Immunoprecipitation
**HIF1A**	BD biosciences (610-727)	Western blotting/IHC/PLA
**HIF1A**	Abcam (ab2185)	IP/ChIP
**HK2**	Abcam (ab104836)	Western blotting
**PDK1**	Abcam (ab110025)	Western blotting
**LDHA**	Abcam (ab101562)	Western blotting
**FLAG M2**	Sigma (F1804)	IP/Western blotting
**PHD2**	Novus bioloigicals (NB100-2219)	IP/Western blotting
**Hydroxy-proline**	Abcam (ab37067)	Western blotting
**K48 ubiquitin**	Abcam (ab140601)	Western blotting
**ACTB**	Sigma (A5441)	Western blotting
