# Supplementary material for: A RASSF1A-HIF1α loop drives Warburg effect in cancer and pulmonary hypertension
Source: Nat Commun. 2019 May 13;10:2130. doi: 10.1038/s41467-019-10044-z (PMC6513860; doi:10.1038/s41467-019-10044-z)
Supplement: Supplementary file 3 — Reporting Summary [file 41467_2019_10044_MOESM3_ESM.pdf]

nature research

Corresponding author(s): Soni Savai Pullamsetti

Last updated by author(s): Apr 2, 2019

Reporting Summary

Nature Research wishes to improve the reproducibility of the work that we publish. This form provides structure for consistency and transparency in reporting. For further information on Nature Research policies, see Authors & Referees and the Editorial Policy Checklist.

Statistics

For all statistical analyses, confirm that the following items are present in the figure legend, table legend, main text, or Methods section.

n/a	Confirmed	
☐	☒	The exact sample size (*n*) for each experimental group/condition, given as a discrete number and unit of measurement
☐	☒	A statement on whether measurements were taken from distinct samples or whether the same sample was measured repeatedly
☐	☒	The statistical test(s) used AND whether they are one- or two-sided<br>*Only common tests should be described solely by name; describe more complex techniques in the Methods section.*
☐	☒	A description of all covariates tested
☐	☒	A description of any assumptions or corrections, such as tests of normality and adjustment for multiple comparisons
☐	☒	A full description of the statistical parameters including central tendency (e.g. means) or other basic estimates (e.g. regression coefficient) AND variation (e.g. standard deviation) or associated estimates of uncertainty (e.g. confidence intervals)
☒	☐	For null hypothesis testing, the test statistic (e.g. *F*, *t*, *r*) with confidence intervals, effect sizes, degrees of freedom and *P* value noted<br>*Give P values as exact values whenever suitable.*
☒	☐	For Bayesian analysis, information on the choice of priors and Markov chain Monte Carlo settings
☒	☐	For hierarchical and complex designs, identification of the appropriate level for tests and full reporting of outcomes
☒	☐	Estimates of effect sizes (e.g. Cohen's *d*, Pearson's *r*), indicating how they were calculated

Our web collection on statistics for biologists contains articles on many of the points above.

Software and code

Policy information about availability of computer code

Data collection	Methods, softwares and equipment for data collection are provided in methods section.
Data analysis	Prism 5 and Prism 6 (GraphPad inc) were used for graph generation and data analyses.

For manuscripts utilizing custom algorithms or software that are central to the research but not yet described in published literature, software must be made available to editors/reviewers. We strongly encourage code deposition in a community repository (e.g. GitHub). See the Nature Research guidelines for submitting code & software for further information.

Data

Policy information about availability of data

All manuscripts must include a data availability statement. This statement should provide the following information, where applicable:

- Accession codes, unique identifiers, or web links for publicly available datasets
- A list of figures that have associated raw data
- A description of any restrictions on data availability

The data supporting the findings of this study are available from the corresponding author upon request.

Field-specific reporting

Please select the one below that is the best fit for your research. If you are not sure, read the appropriate sections before making your selection.

☒ Life sciences ☐ Behavioural & social sciences ☐ Ecological, evolutionary & environmental sciences

For a reference copy of the document with all sections, see nature.com/documents/nr-reporting-summary-flat.pdf

Life sciences study design

All studies must disclose on these points even when the disclosure is negative.

Sample size	Sample size was selected on the basis of relevant experience in our lab and previous studies by our and others.
Data exclusions	No data exclusion was performed.
Replication	All experiments were repeated at least 3 times in different biological replicates (in case of primary cells) or different passages (in case of cell lines), unless stated differently in figure legends.
Randomization	No randomization method was used.
Blinding	Investigators who were performing histology and MRI are blinded.

Reporting for specific materials, systems and methods

We require information from authors about some types of materials, experimental systems and methods used in many studies. Here, indicate whether each material, system or method listed is relevant to your study. If you are not sure if a list item applies to your research, read the appropriate section before selecting a response.

Materials & experimental systems

n/a	Involved in the study	
☐	☒	Antibodies
☐	☒	Eukaryotic cell lines
☒	☐	Palaeontology
☐	☒	Animals and other organisms
☐	☒	Human research participants
☒	☐	Clinical data

Methods

n/a	Involved in the study	
☒	☐	ChIP-seq
☒	☐	Flow cytometry
☒	☐	MRI-based neuroimaging

Antibodies

Antibodies used

RASSF1A Abcam (ab23950) Western blotting
RASSF1 Santa Cruz (sc-18722) Immunohistochemistry
RASSF1A Biorbyt (orb11328) Proximity ligation assay
RASSF1A Abcam (ab91212) Immunoprecipitation
HIF1A BD biosciences (610-727) Western blotting/IHC/PLA
HIF1A Abcam (ab2185) IP/ChIP
HK2 Abcam (ab104836) Western blotting
PDK1 Abcam (ab110025) Western blotting
LDHA Abcam (ab101562) Western blotting
FLAG M2 Sigma (F1804) IP/Western blotting
PHD2 Novus bioloigicals (NB100-2219) IP/Western blotting
Hydroxy-proline Abcam (ab37067) Western blotting
K48 ubiquitin Abcam (ab140601) Western blotting
ACTB Sigma (A5441)

Validation

Validations are based on the datasheets from the manufacturers.

Eukaryotic cell lines

Policy information about cell lines

Cell line source(s)	HEK293 nd HeLa cells were obtained from ATCC.
Authentication	None of the cell lines used were authenticated.
Mycoplasma contamination	All cell lines are tested mycoplasma regularly and the cell lines used in the study are mycoplasma free
Commonly misidentified lines (See ICLAC register)	No commonly misidentified cell lines were used.

Animals and other organisms

Policy information about studies involving animals; ARRIVE guidelines recommended for reporting animal research

Laboratory animals	The current study used laboratory mouse strain C57Bl/6 having Rassf1a gene knockout (males 12 to 14 weeks old).
Wild animals	The study did not involve wild animals.
Field-collected samples	The study did not involve samples collected from the field.
Ethics oversight	The experiments were performed in accordance with the US National Institutes of Health Guidelines on the Use of Laboratory Animals. Both the University Animal Care Committee and the federal authorities for animal research of the Regierungspräsidium Giessen and Darmstadt (Hessen, Germany) approved the study protocol (B2/325).

Note that full information on the approval of the study protocol must also be provided in the manuscript.

Human research participants

Policy information about studies involving human research participants

Population characteristics	Human research participants includes male and female, all of whom were characterised according to standard clinical procedure into the different groups. The patients characterisitcs are maintained in Giessen bio bank (for IPAH patients) and Lung Biobank Heidelberg (for NSCLC patients).
Recruitment	N/A
Ethics oversight	For IPAH or control donor tissue donation:<br>The study protocol for tissue donation was approved by the ethics committee (Ethik Kommission am Fachbereich Humanmedizin der Justus Liebig Universität Giessen) of the University Hospital Giessen (Giessen, Germany) in accordance with national law and with Good Clinical Practice/International Conference on Harmonisation guidelines. Written informed consent was obtained from each individual patient or the patient's next of kin (AZ 58/15).<br><br>For cancer tissue and cells:<br>Biomaterials and data from NSCLC patients were provided by the Lung Biobank Heidelberg, a member of the accredited Tissue Bank of the National Center for Tumor Diseases (NCT) Heidelberg, the BioMaterialBank Heidelberg and the Biobank platform of the German Center for Lung Research (DZL). All subjects gave their informed consent for inclusion before they participated in the study. The study was conducted in accordance with the Declaration of Helsinki. The use of biomaterial and data for this study was approved by the local ethics committee of the Medical Faculty Heidelberg (S-270/2001).

Note that full information on the approval of the study protocol must also be provided in the manuscript.
