# Supplementary material for: A RASSF1A-HIF1α loop drives Warburg effect in cancer and pulmonary hypertension
Source: Nat Commun. 2019 May 13;10:2130. doi: 10.1038/s41467-019-10044-z (PMC6513860; doi:10.1038/s41467-019-10044-z)

Fig 1e

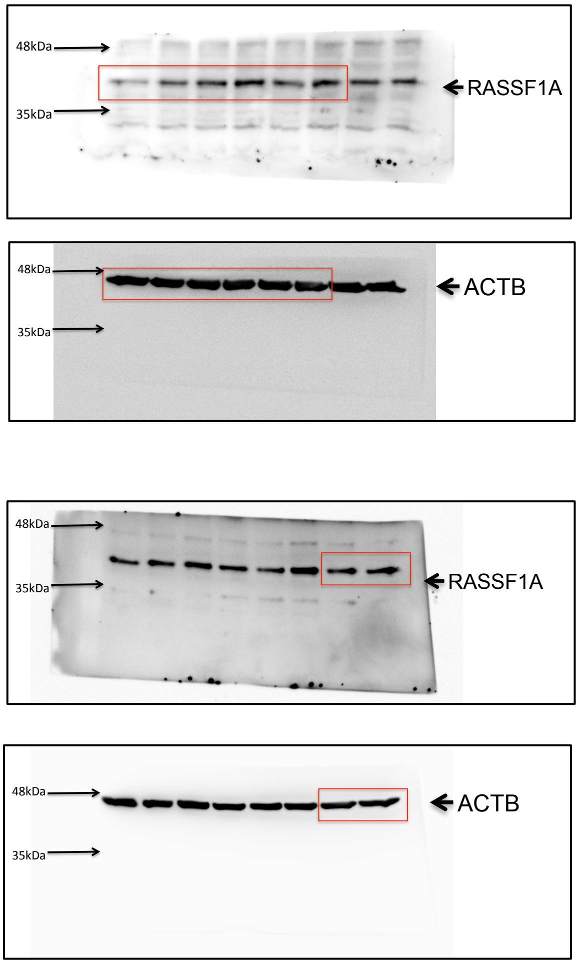

Fig 1e-g

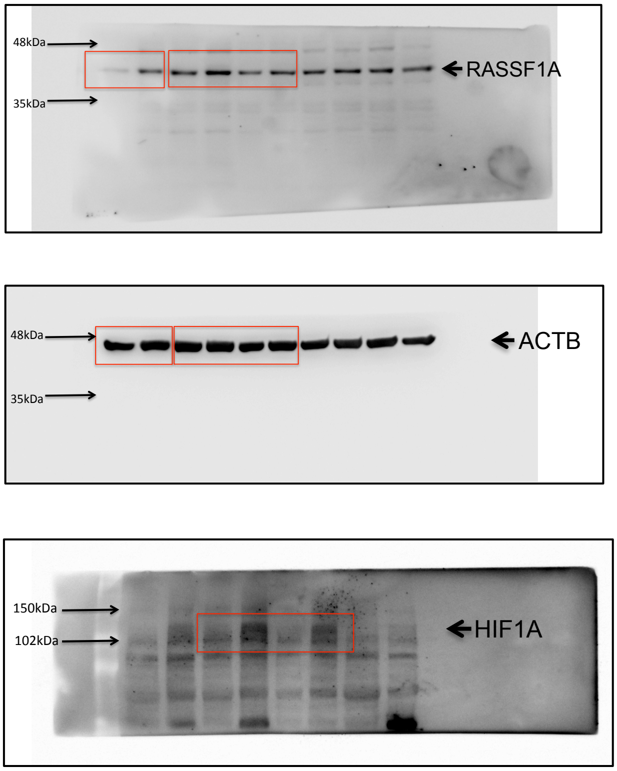

Fig 2a

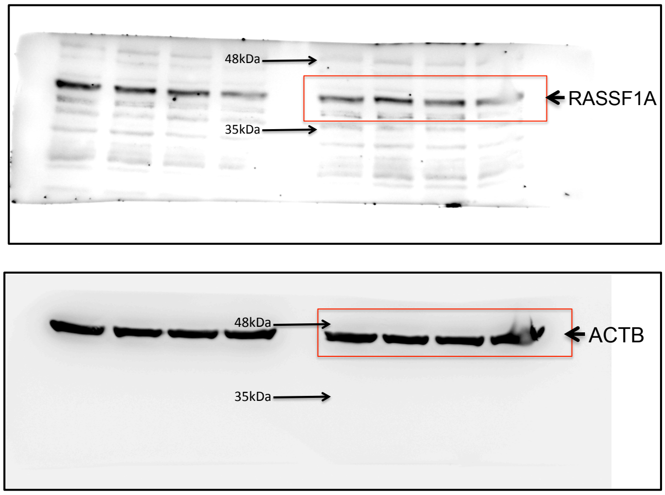

Fig 2b

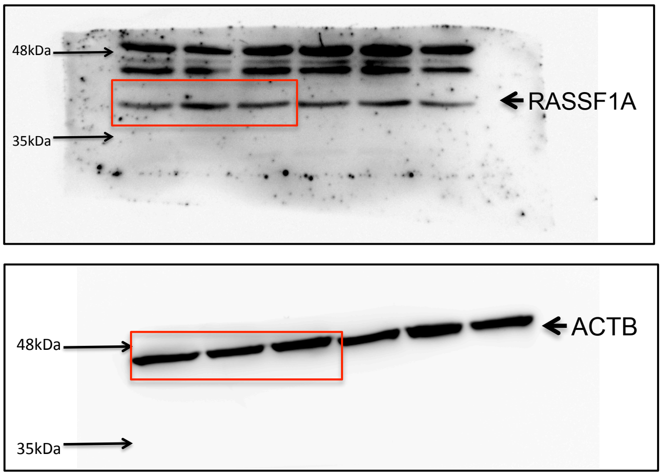

Fig 2c

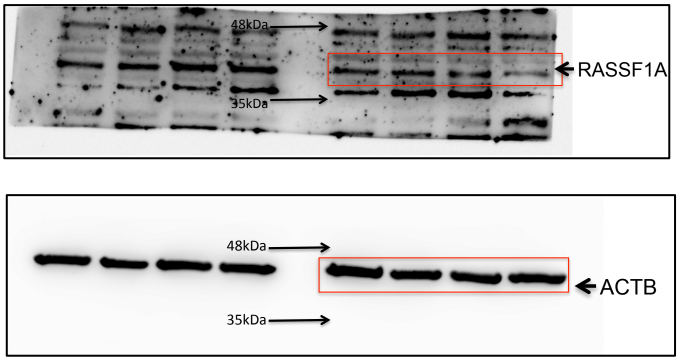

Fig 2d

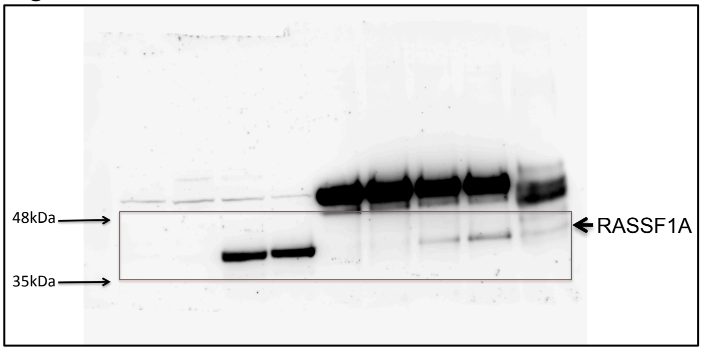

Fig 2e

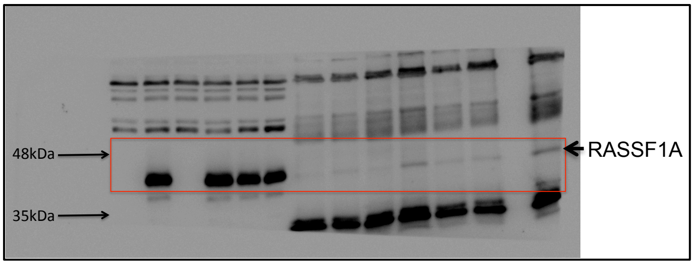

Fig 2f

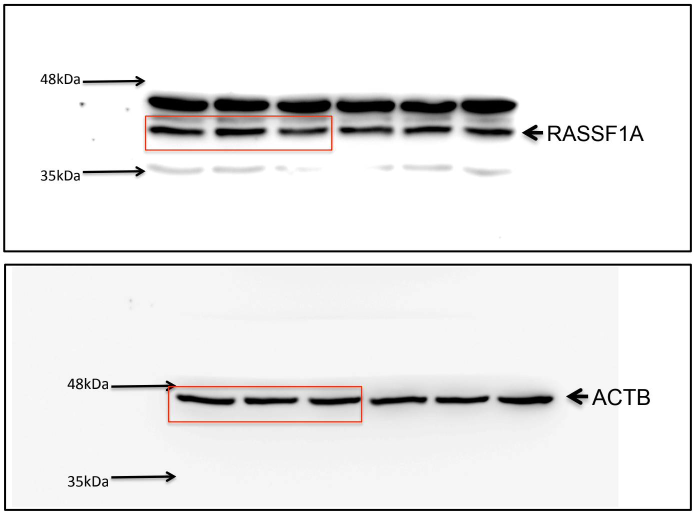

Fig 2g

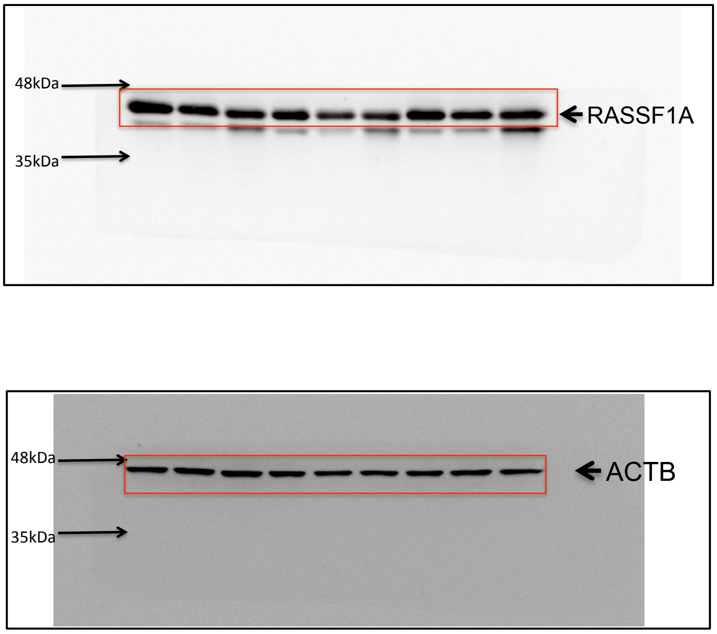

Fig 2h

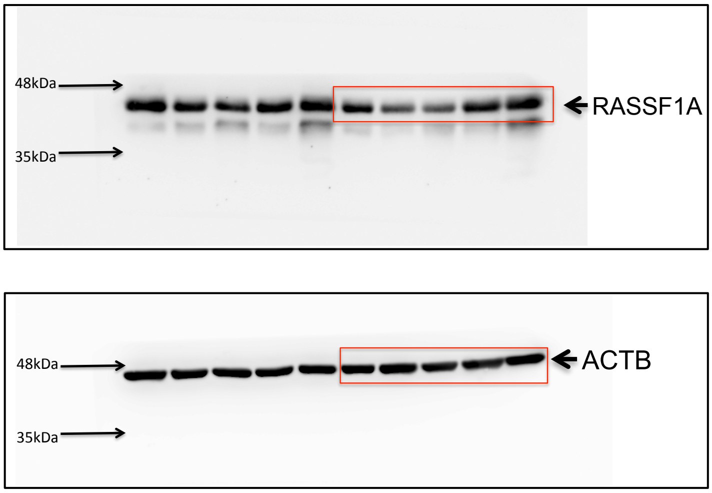

Fig 2i

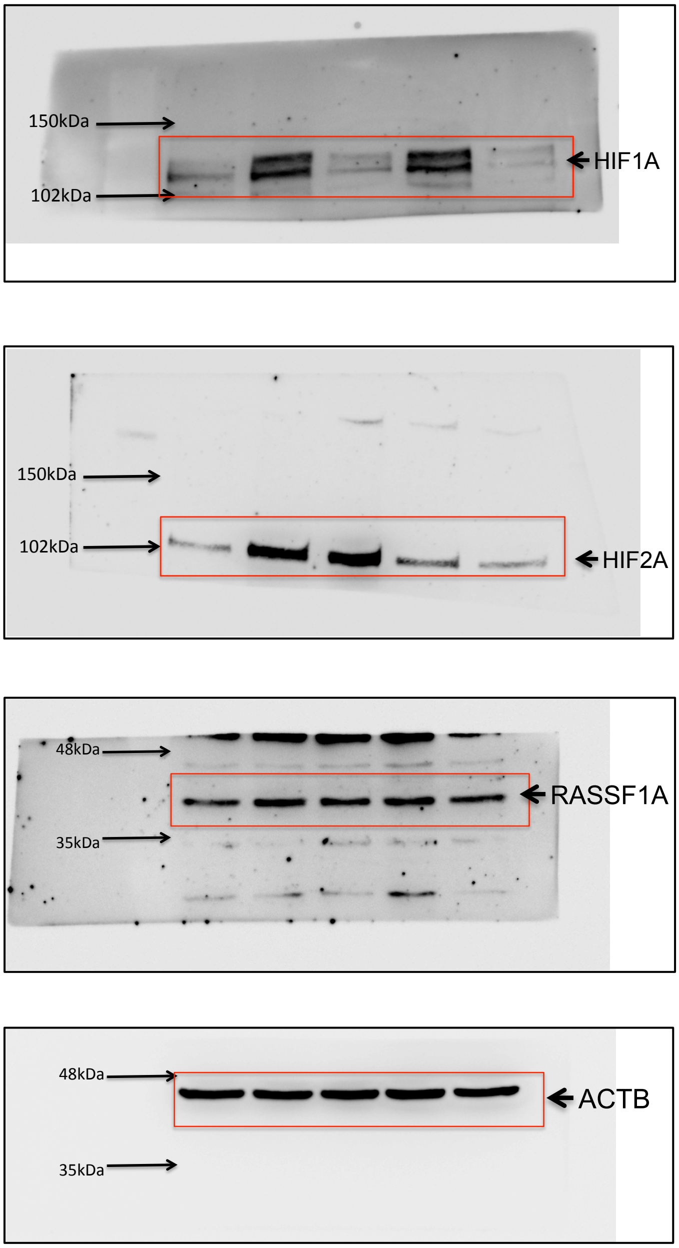

Fig 3e

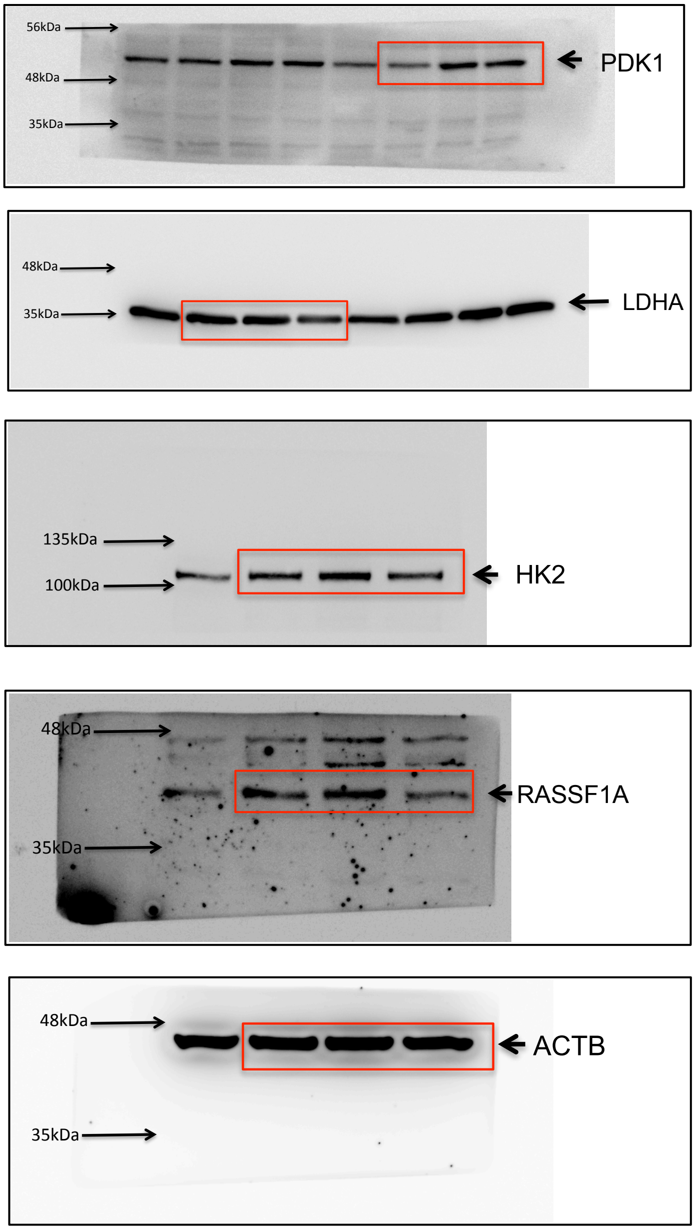
56kDa
48kDa
35kDa
PDK1
48kDa
35kDa
LDHA
135kDa
100kDa
HK2
48kDa
35kDa
RASSF1A
48kDa
35kDa
ACTB

Fig 3g

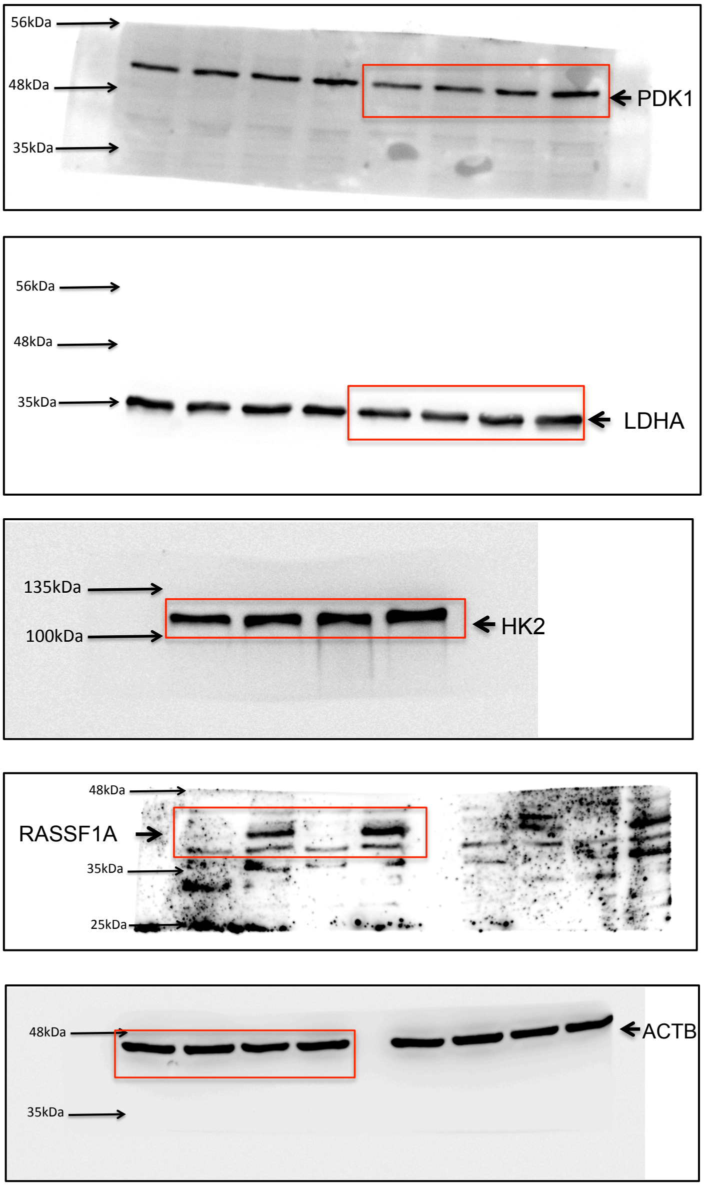
56kDa
48kDa
35kDa
PDK1
56kDa
48kDa
35kDa
LDHA
135kDa
100kDa
HK2
48kDa
RASSF1A
35kDa
25kDa
48kDa
35kDa
ACTB

Fig 4a

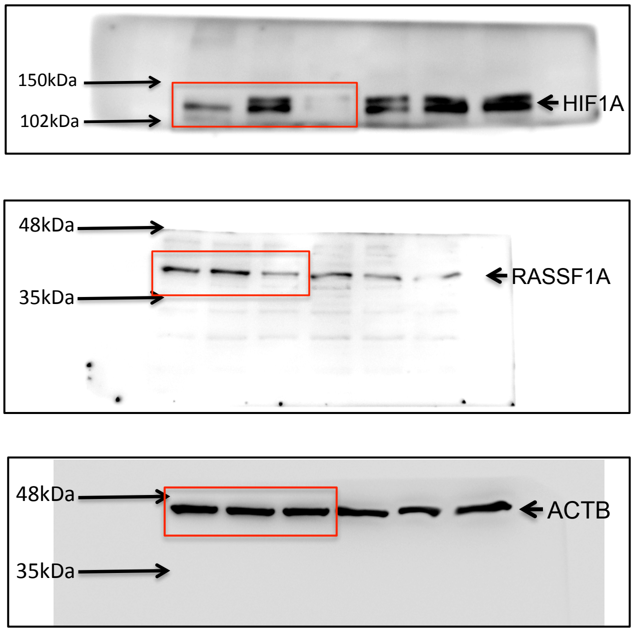

Fig 4e

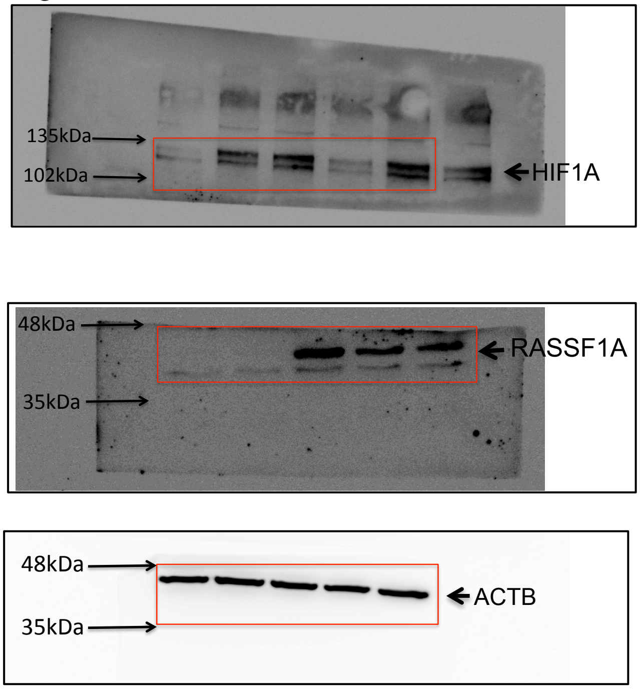

Fig 4b

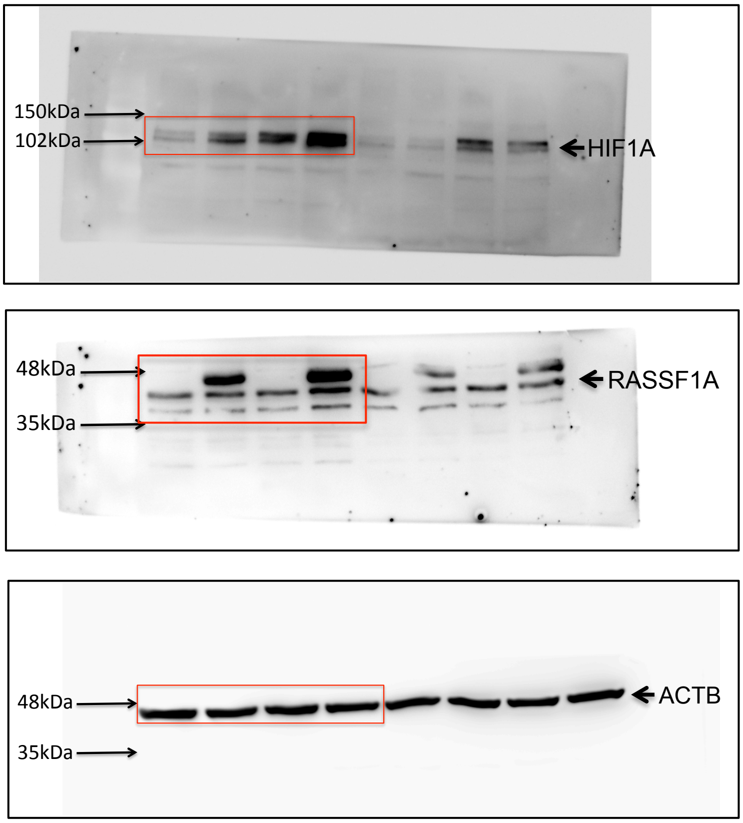

Fig 5a

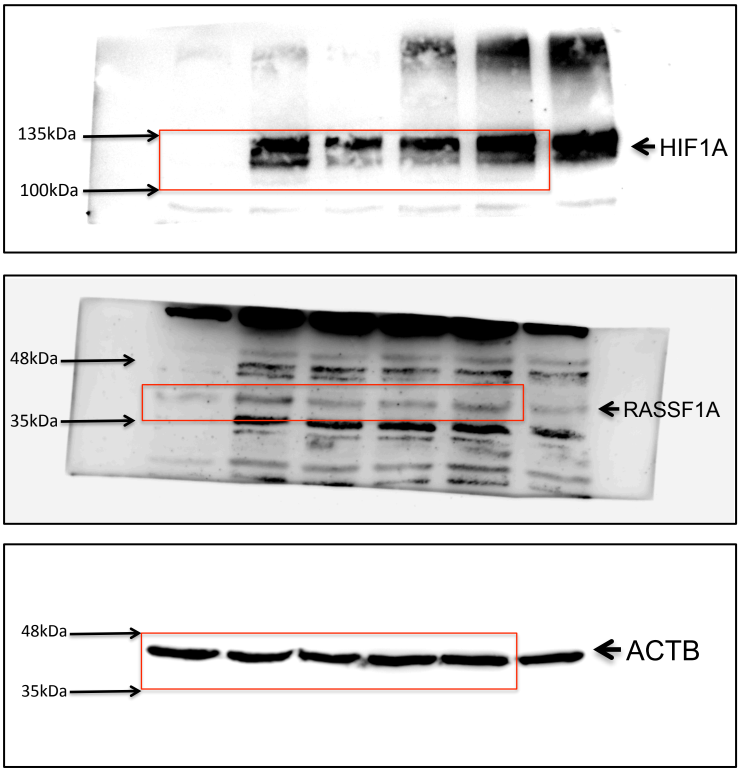
135kDa
100kDa
HIF1A
48kDa
35kDa
RASSF1A
48kDa
35kDa
ACTB

Fig 5b

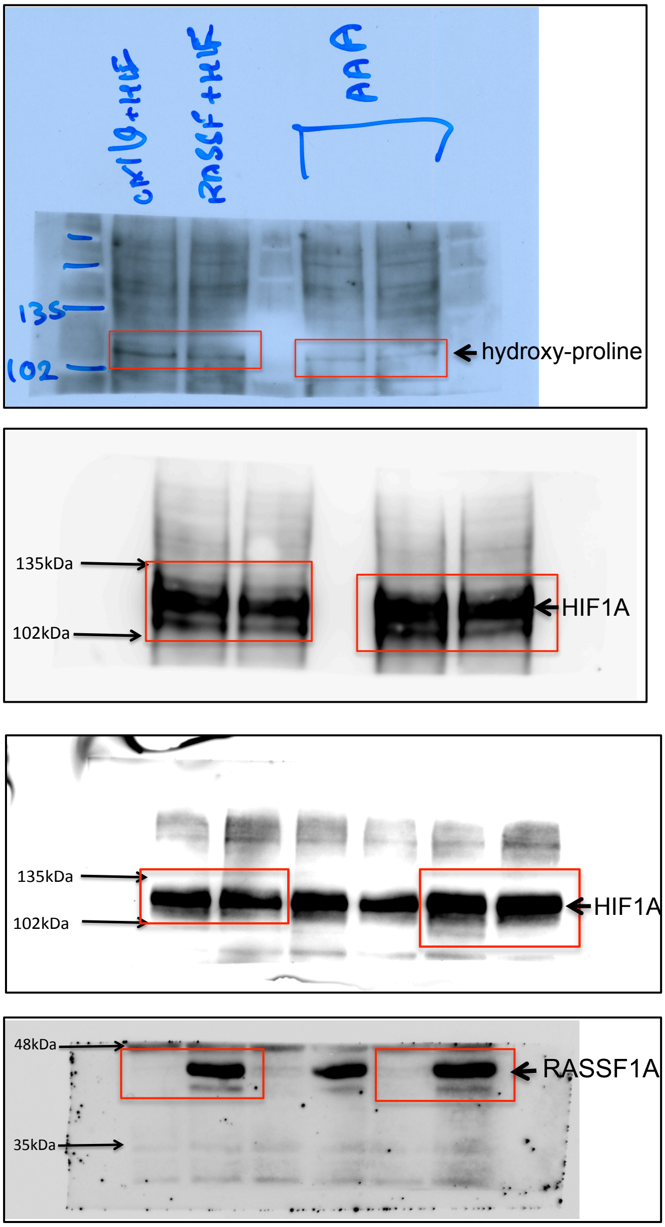
RASSF+HIF
AAA
135
102
hydroxy-proline
135kDa
102kDa
HIF1A
135kDa
102kDa
HIF1A
48kDa
35kDa
RASSF1A

Fig 5c

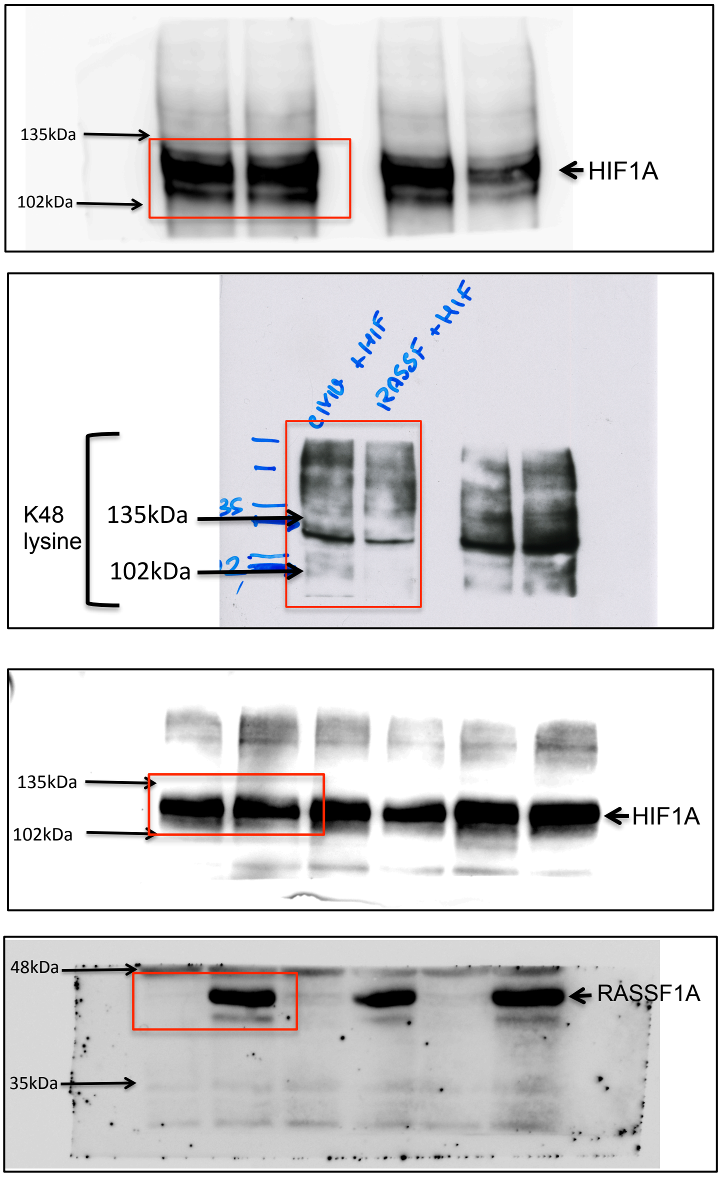
135kDa
102kDa
HIF1A
K48 lysine
135kDa
102kDa
135kDa
102kDa
HIF1A
48kDa
35kDa
RASSF1A

Fig 5d

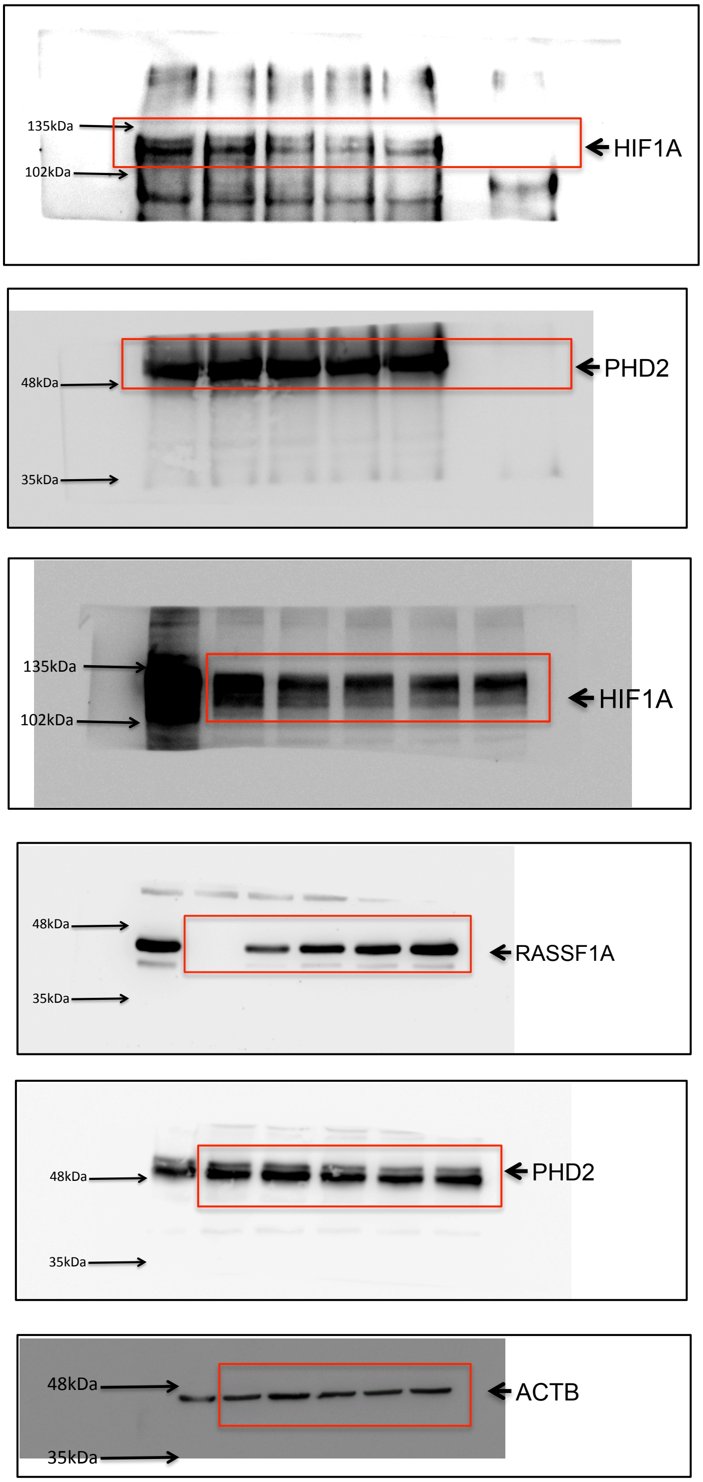
135kDa
102kDa
HIF1A
48kDa
35kDa
PHD2
135kDa
102kDa
HIF1A
48kDa
35kDa
RASSF1A
48kDa
35kDa
PHD2
48kDa
35kDa
ACTB

Fig 5e

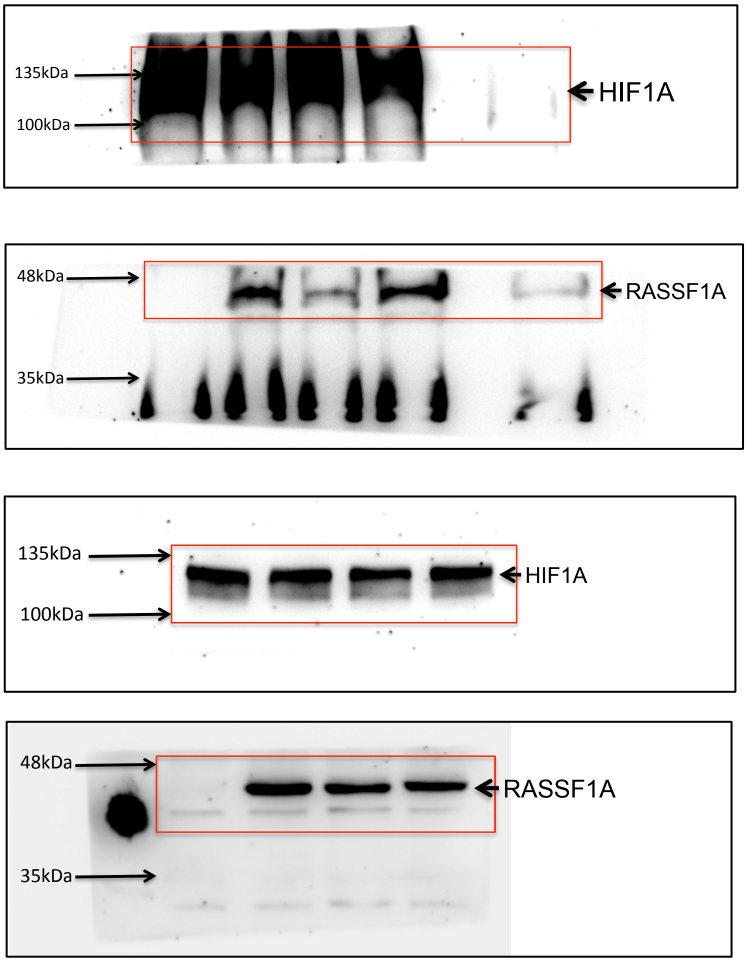

135kDa
100kDa
HIF1A
48kDa
35kDa
RASSF1A
135kDa
100kDa
HIF1A
48kDa
35kDa
RASSF1A

Fig 5f

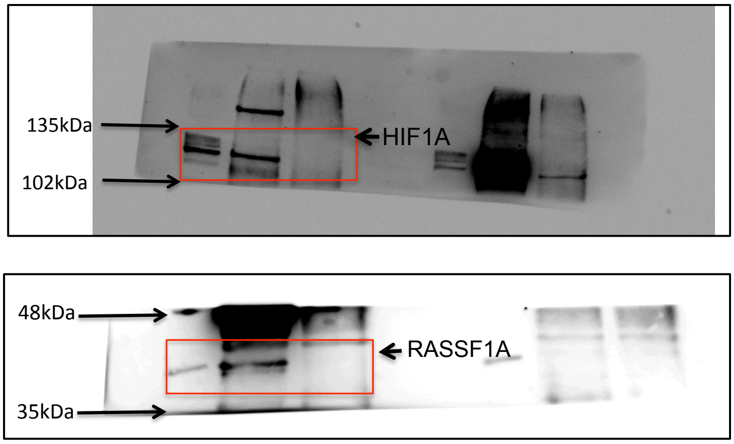

135kDa
102kDa
HIF1A
48kDa
35kDa
RASSF1A

Fig 6e

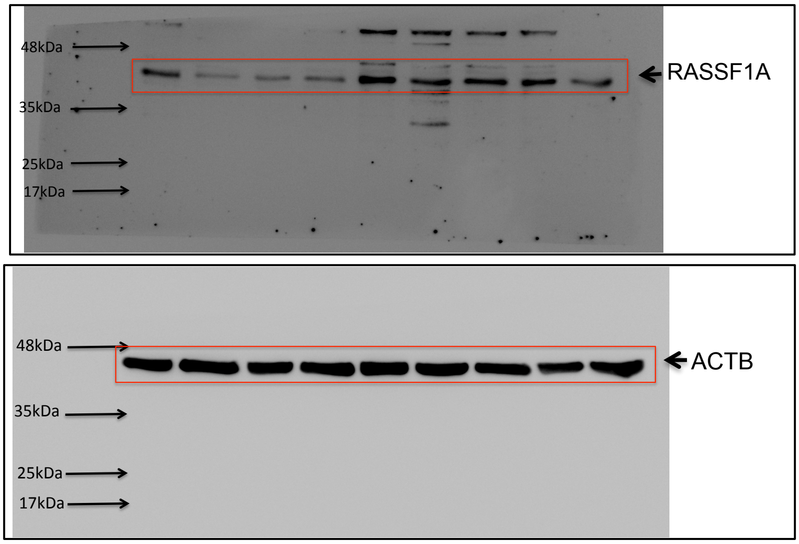
48kDa
35kDa
25kDa
17kDa
RASSF1A
48kDa
35kDa
25kDa
17kDa
ACTB

Fig 6h

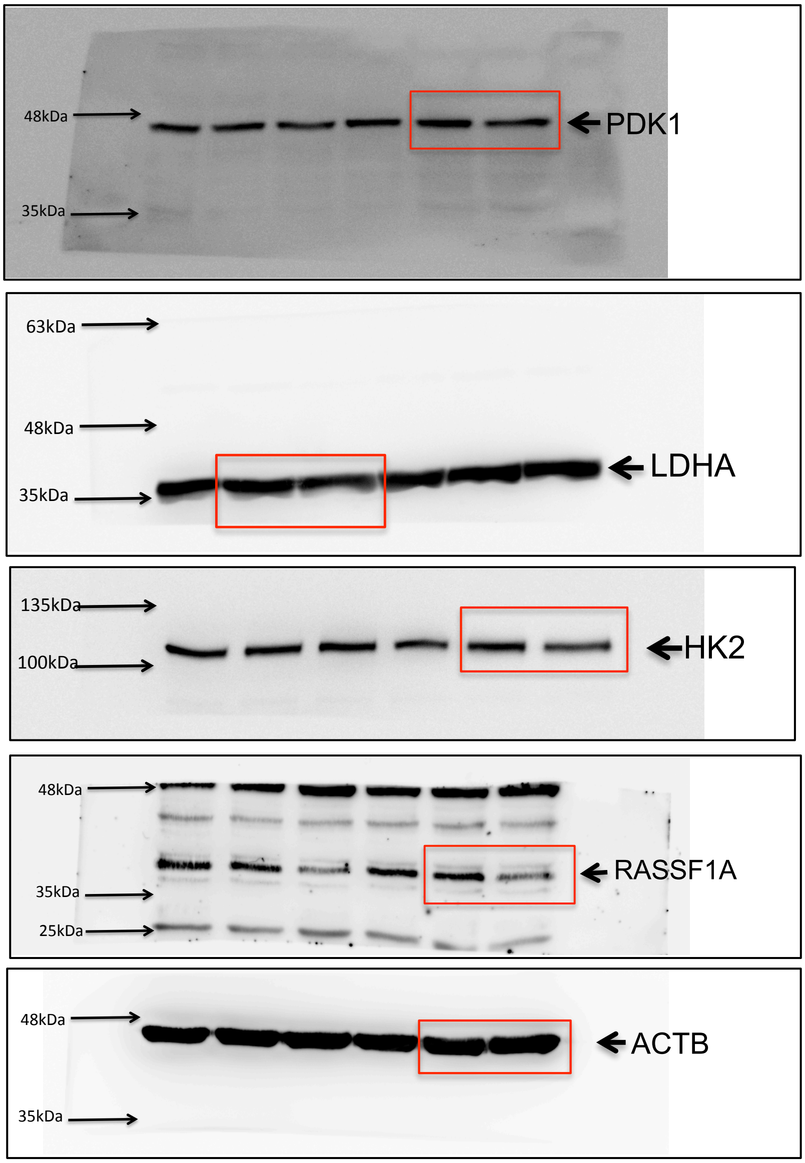
48kDa
35kDa
PDK1
63kDa
48kDa
35kDa
LDHA
135kDa
100kDa
HK2
48kDa
35kDa
25kDa
RASSF1A
48kDa
35kDa
ACTB

Fig 8a

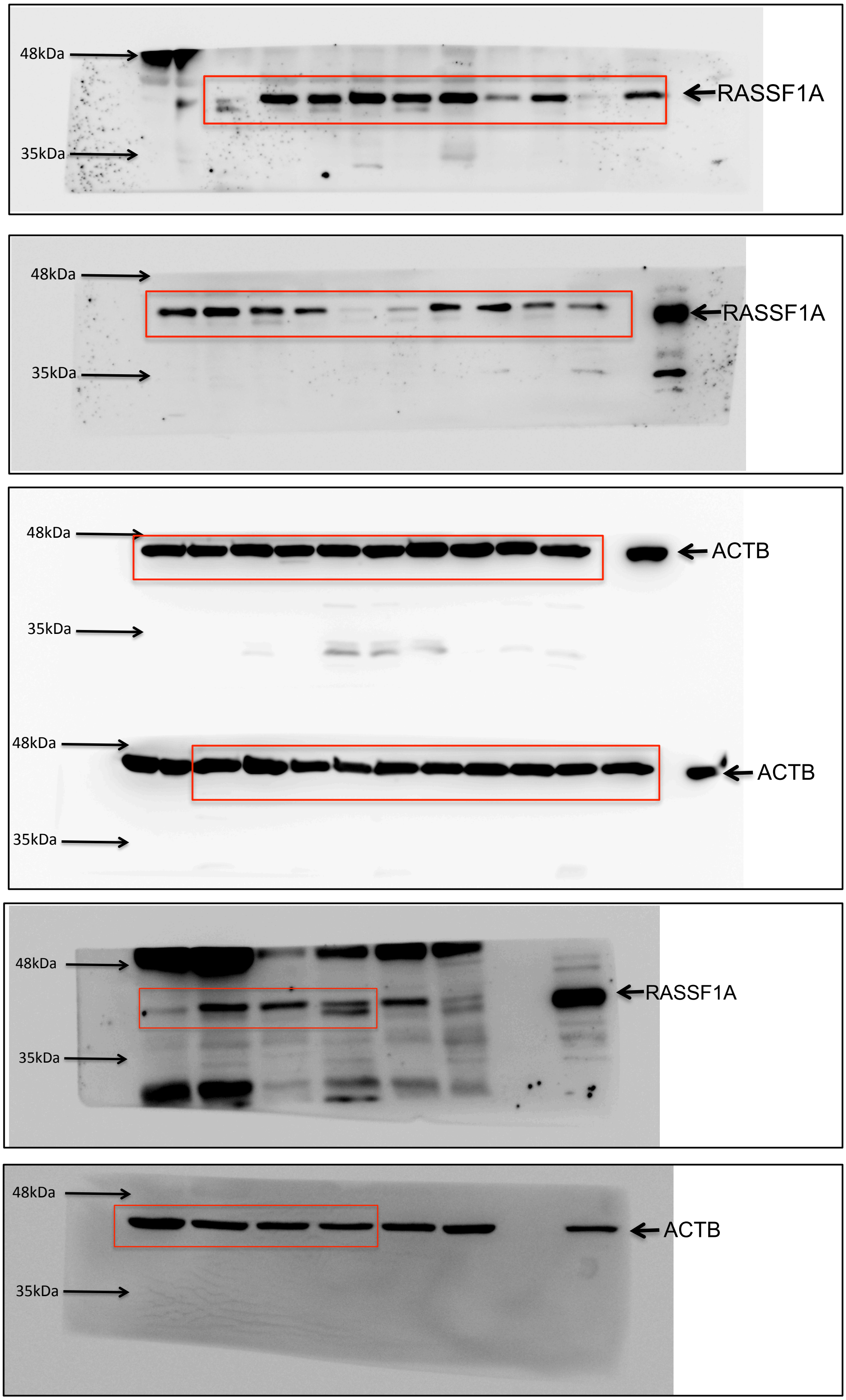
48kDa
35kDa
RASSF1A
48kDa
35kDa
RASSF1A
48kDa
35kDa
ACTB
48kDa
35kDa
ACTB
48kDa
35kDa
RASSF1A
48kDa
35kDa
ACTB

Fig 8

Fig 8c

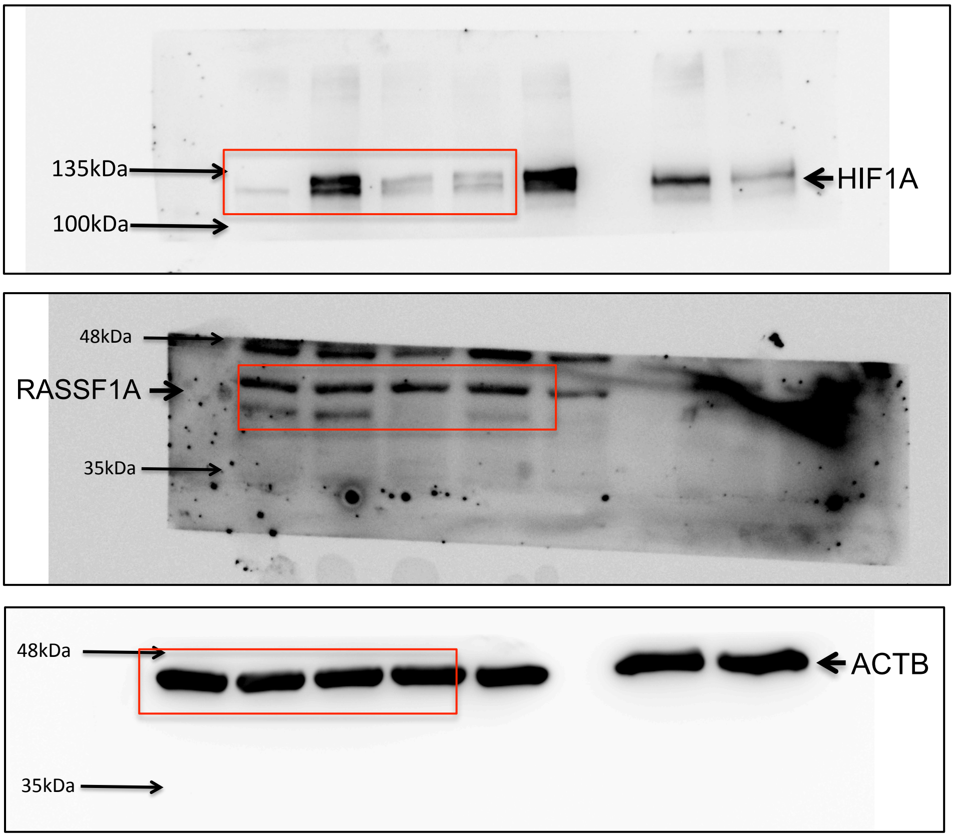

Fig 8e

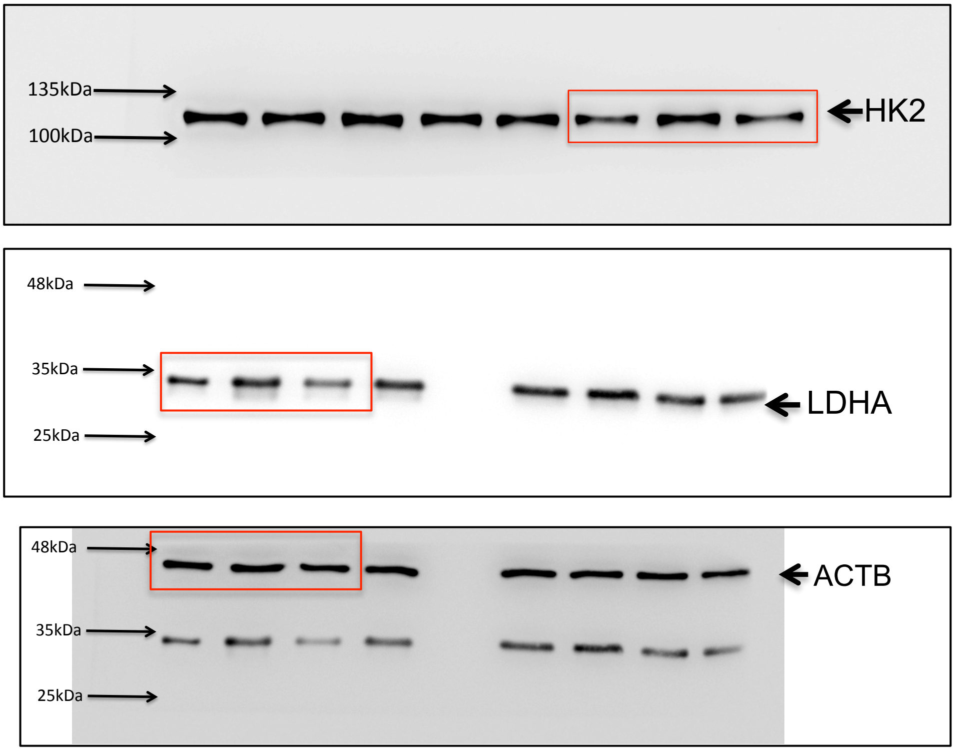

Fig 8g

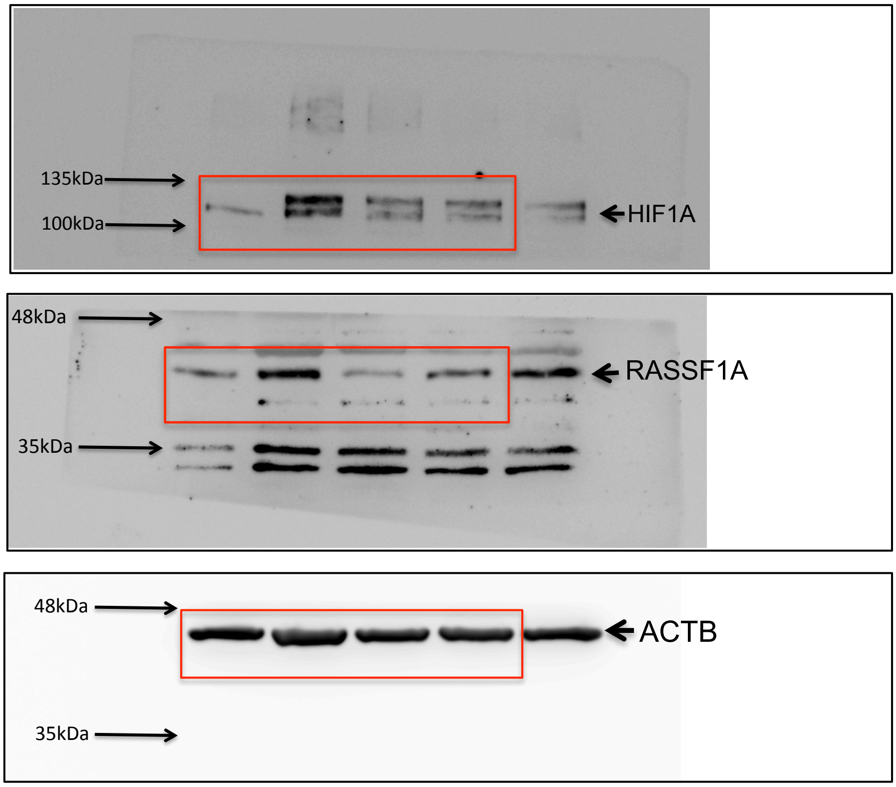
135kDa
100kDa
HIF1A
48kDa
RASSF1A
35kDa
48kDa
ACTB
35kDa

Fig 8i

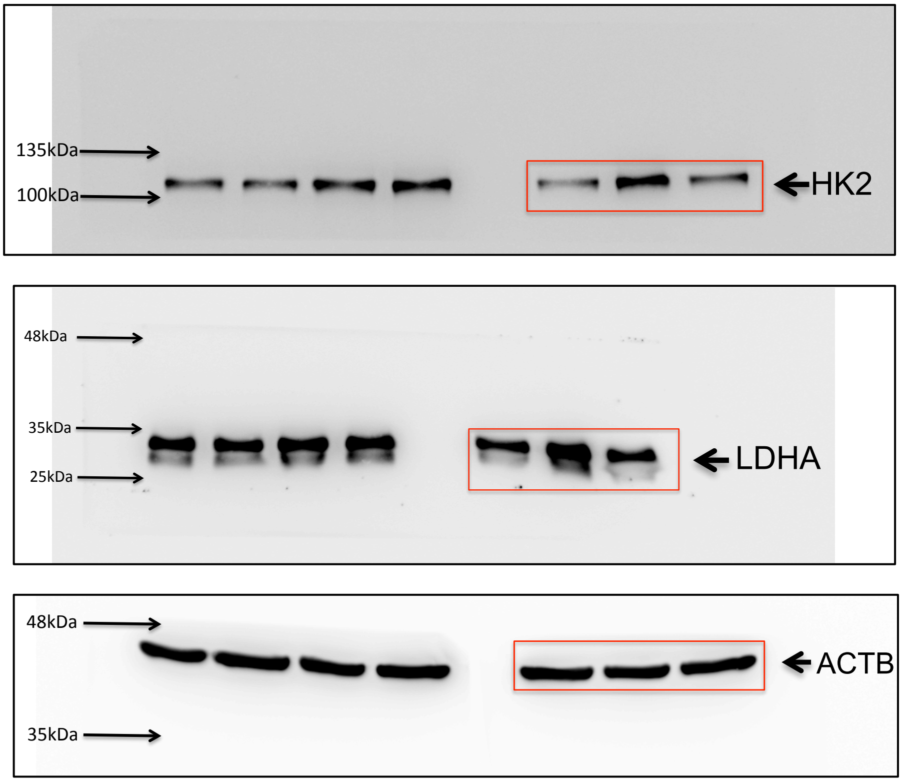
135kDa
100kDa
HK2
48kDa
35kDa
LDHA
25kDa
48kDa
ACTB
35kDa

Supplementary Fig 1b

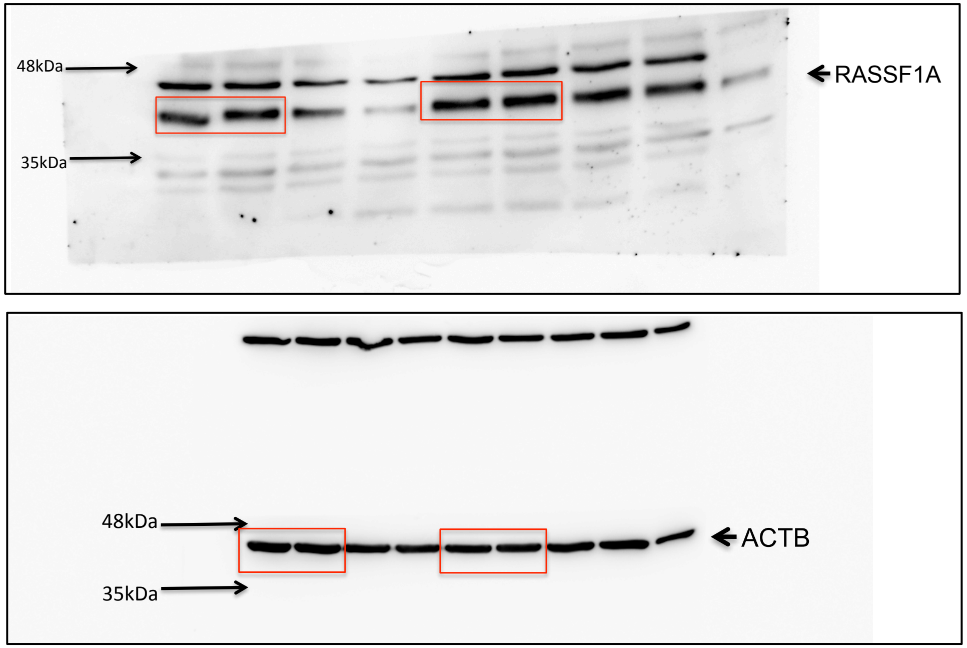

Supplementary Fig 1d

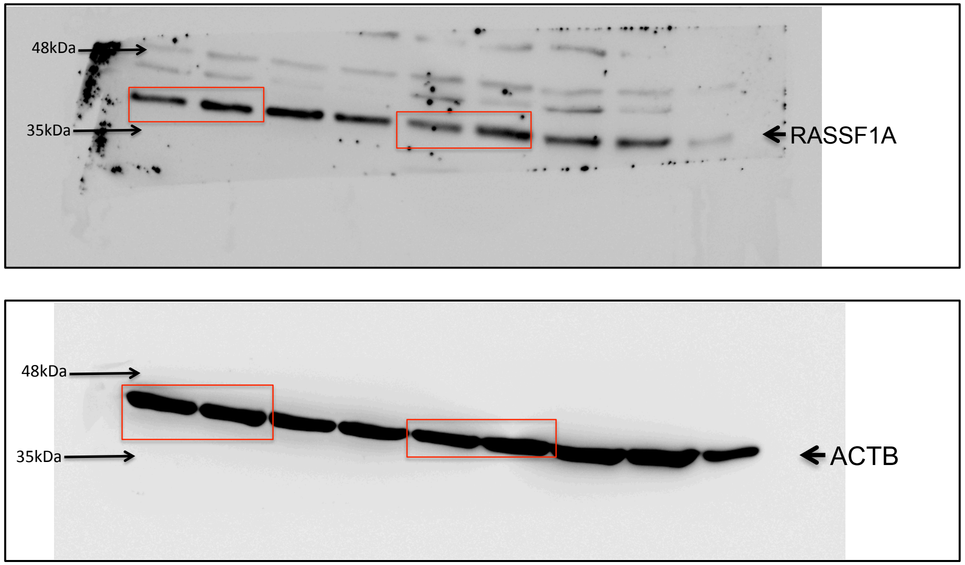

Supplementary Fig 2a

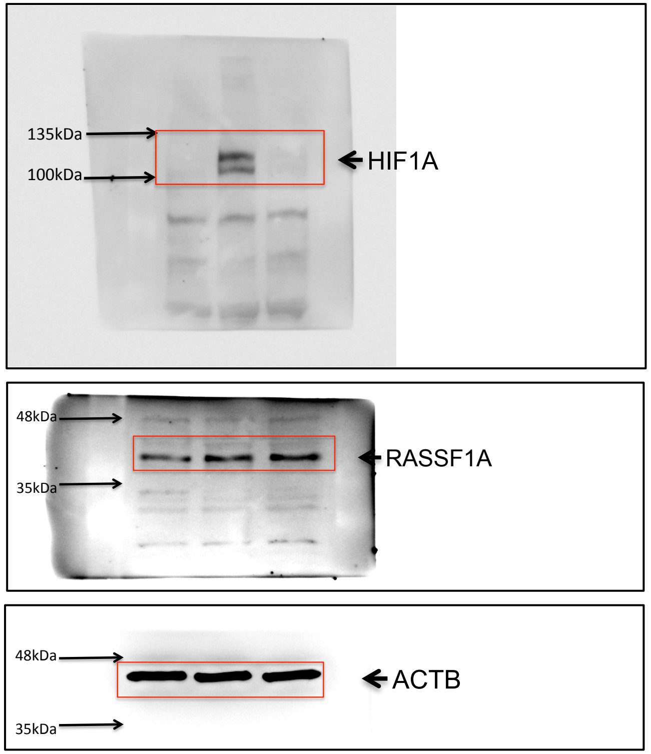

Supplementary Fig 2b

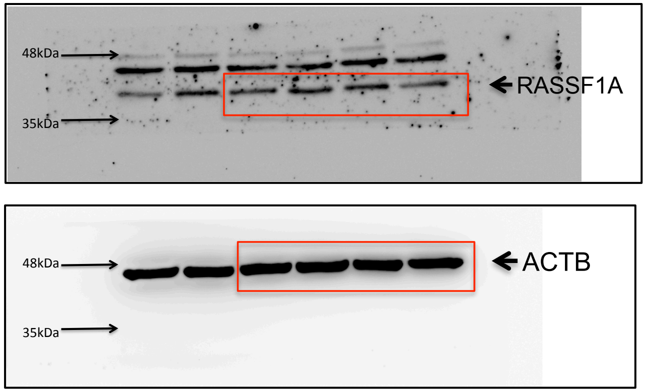

Supplementary Fig 2c

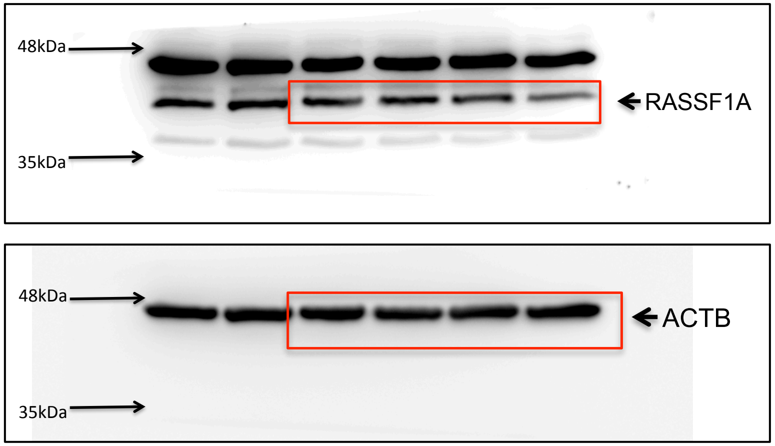

Supplementary Fig 2e

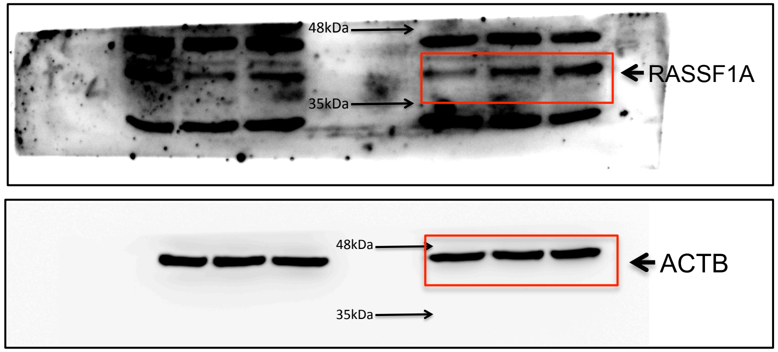

Supplementary Fig 2f

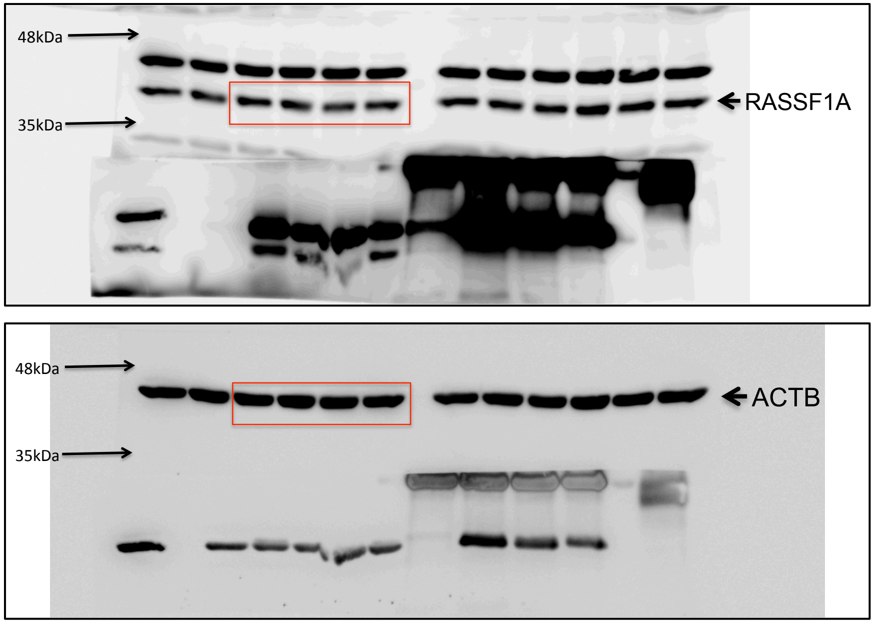

Supplementary Fig 2g

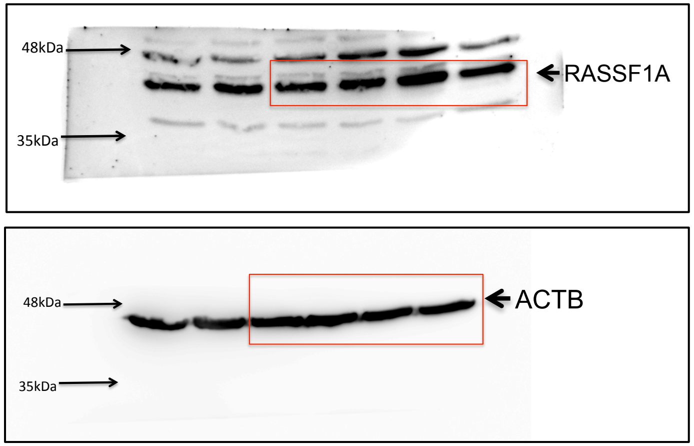

Supplementary Fig 2h

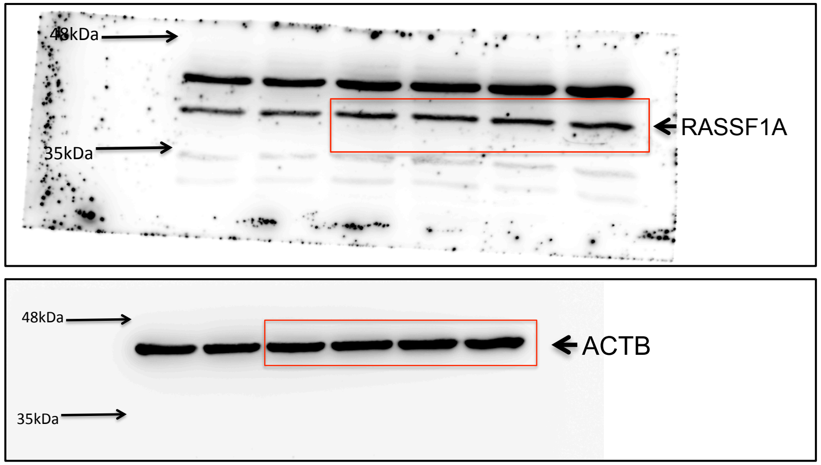

Supplementary Fig 2i

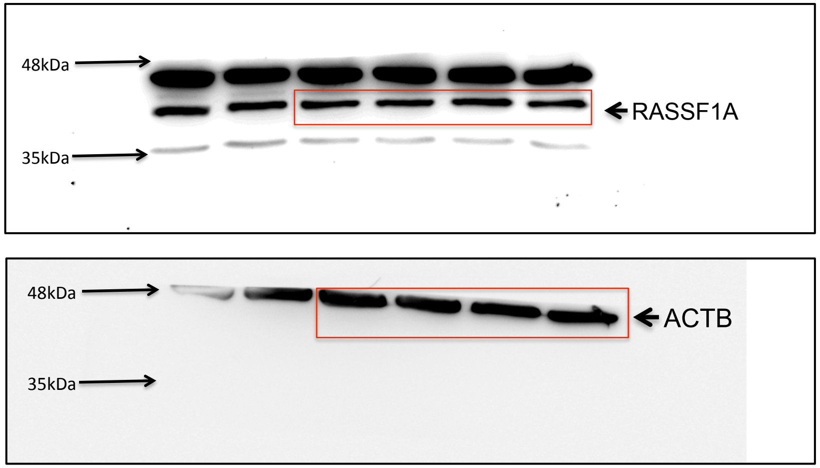

Supplementary Fig 2j

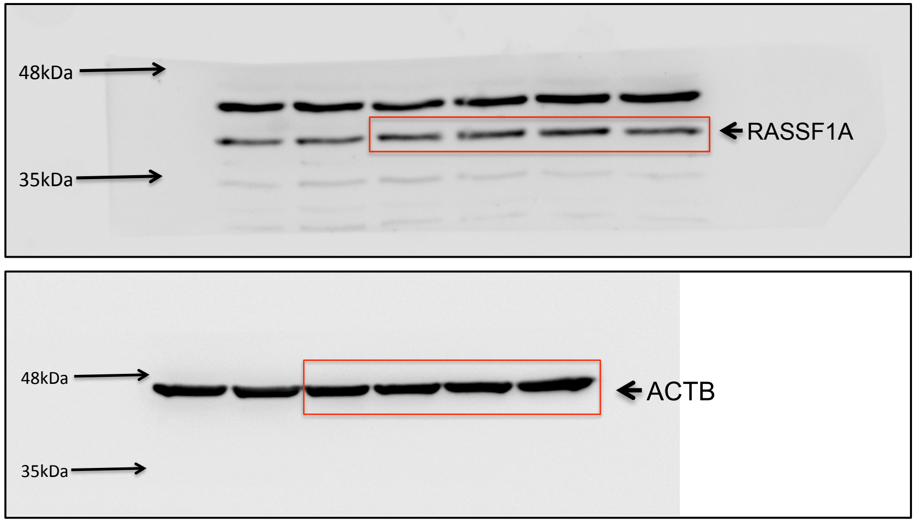

Supplementary Fig 3c

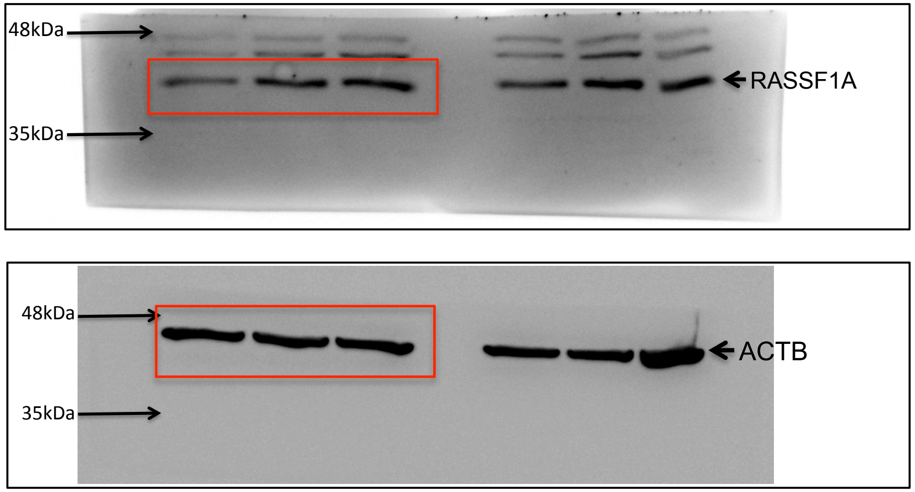

Supplementary Fig 3a

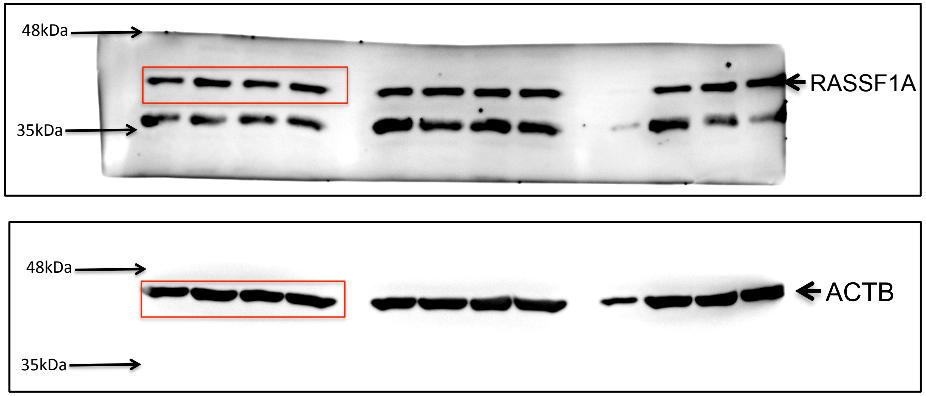

Supplementary Fig 3d

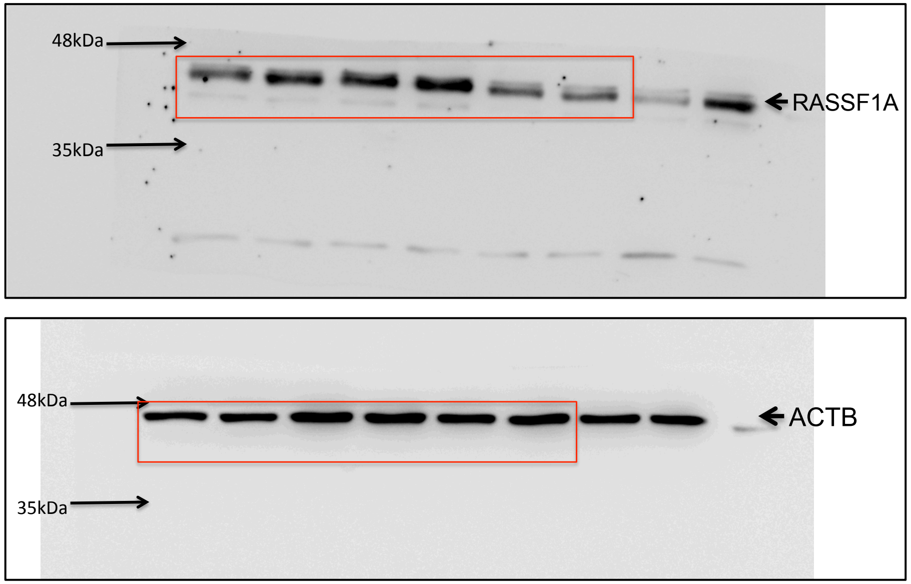

Supplementary Fig 4b

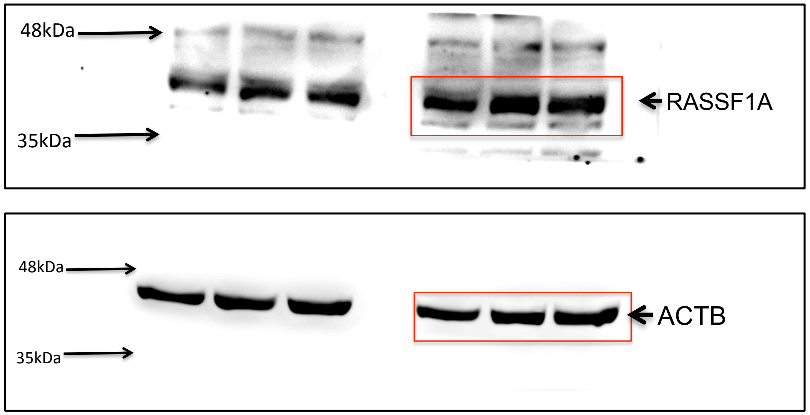

Supplementary Fig 6a

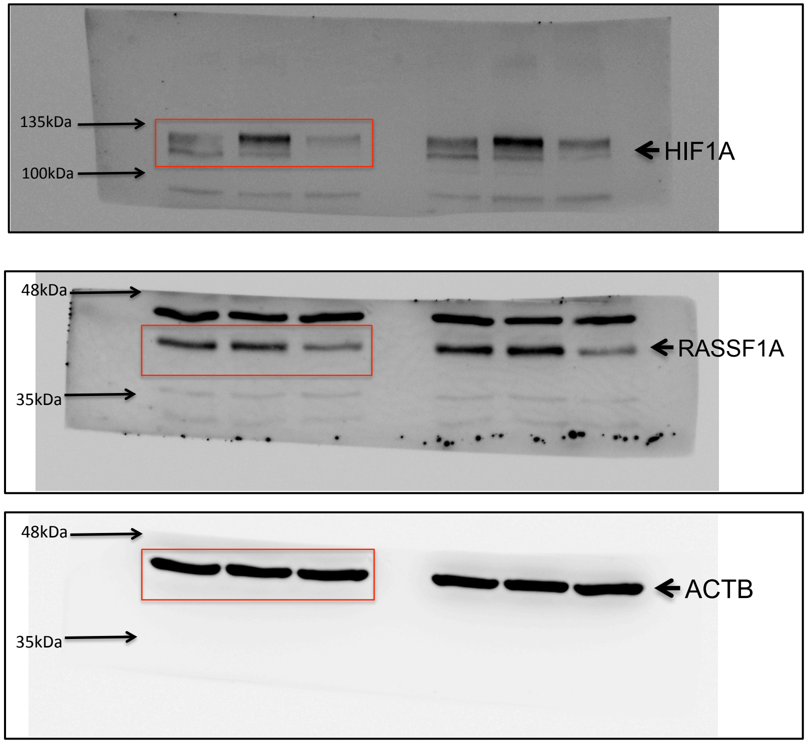

Supplementary Fig 6b

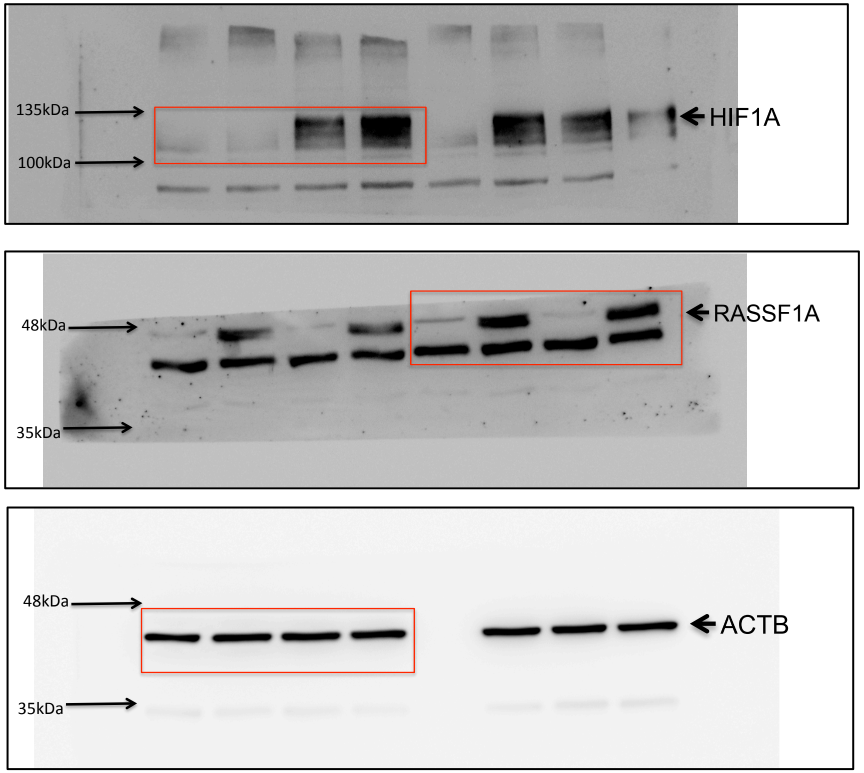

Supplementary Fig 6c

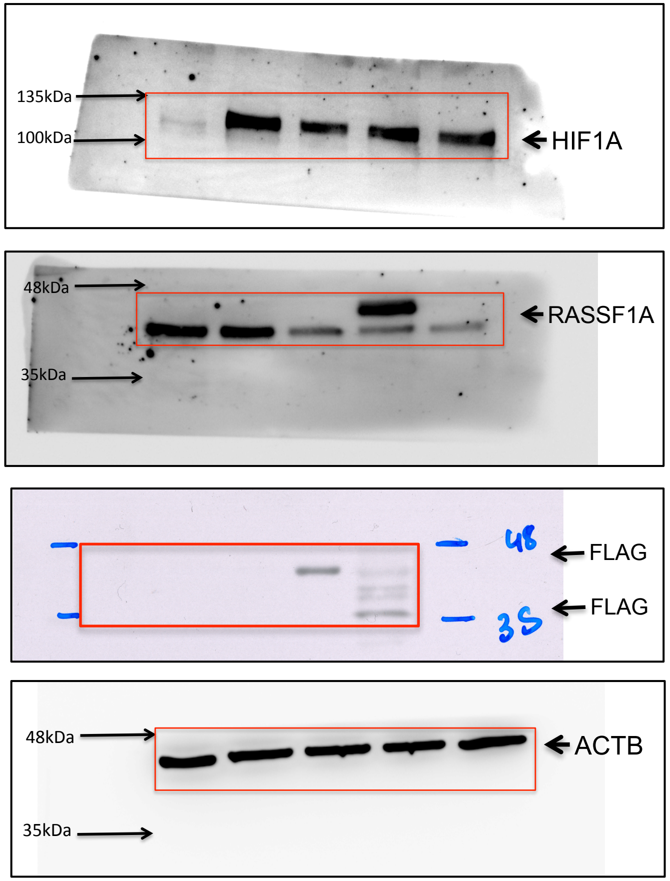

Supplementary Fig 6e

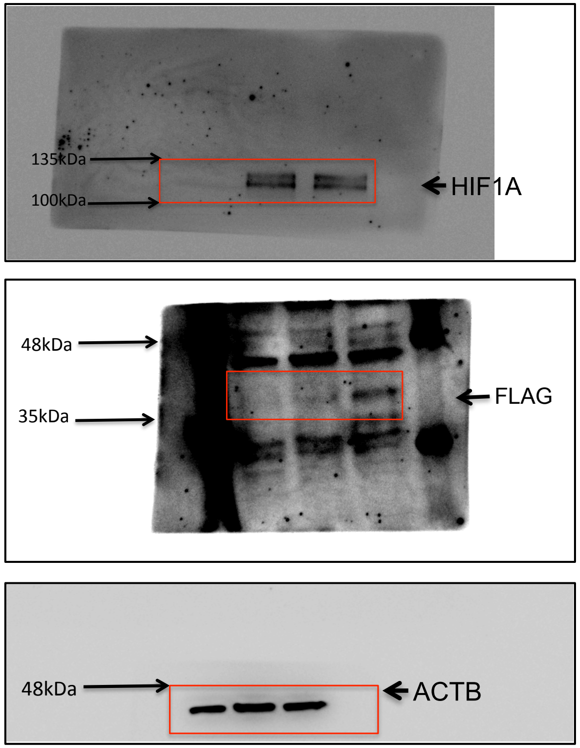

Supplementary Fig 7b

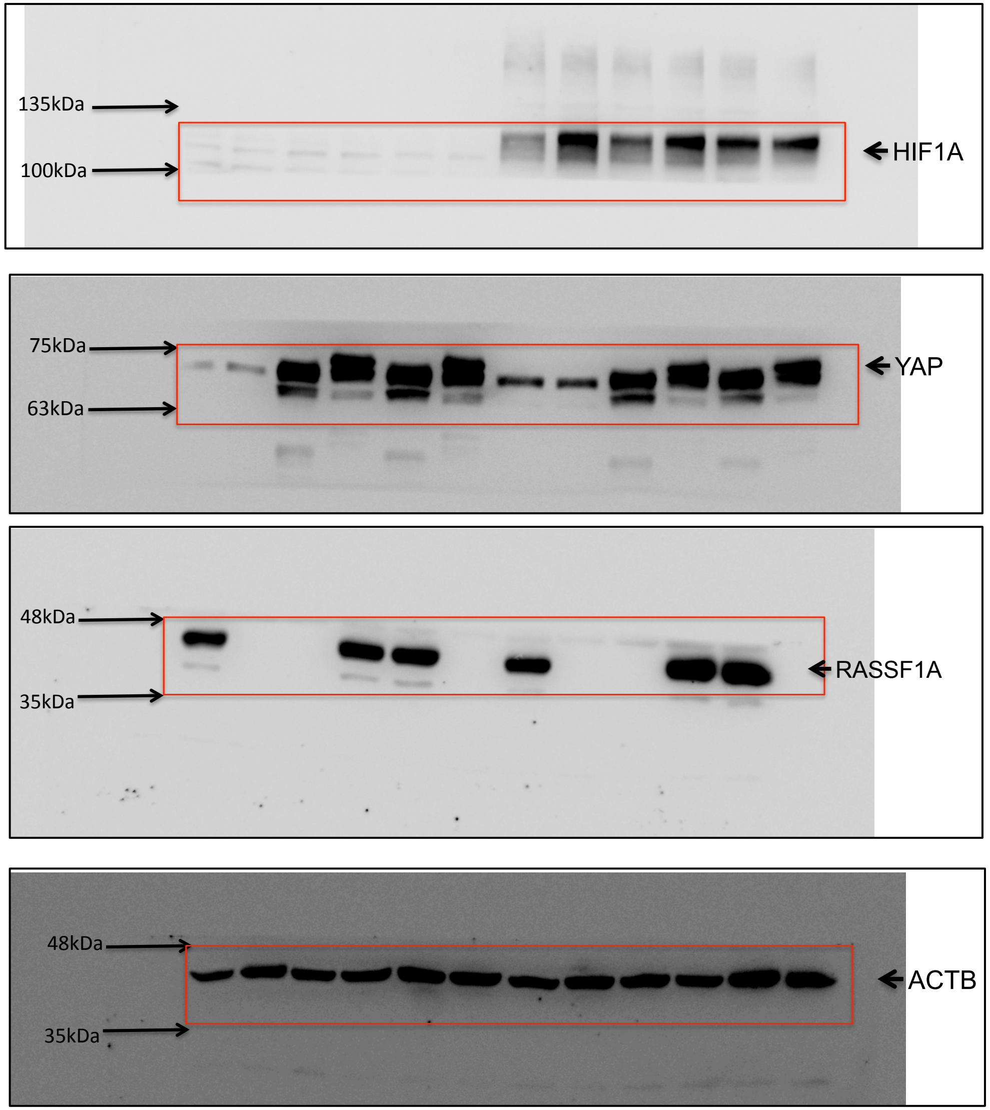

Supplementary Fig 8a

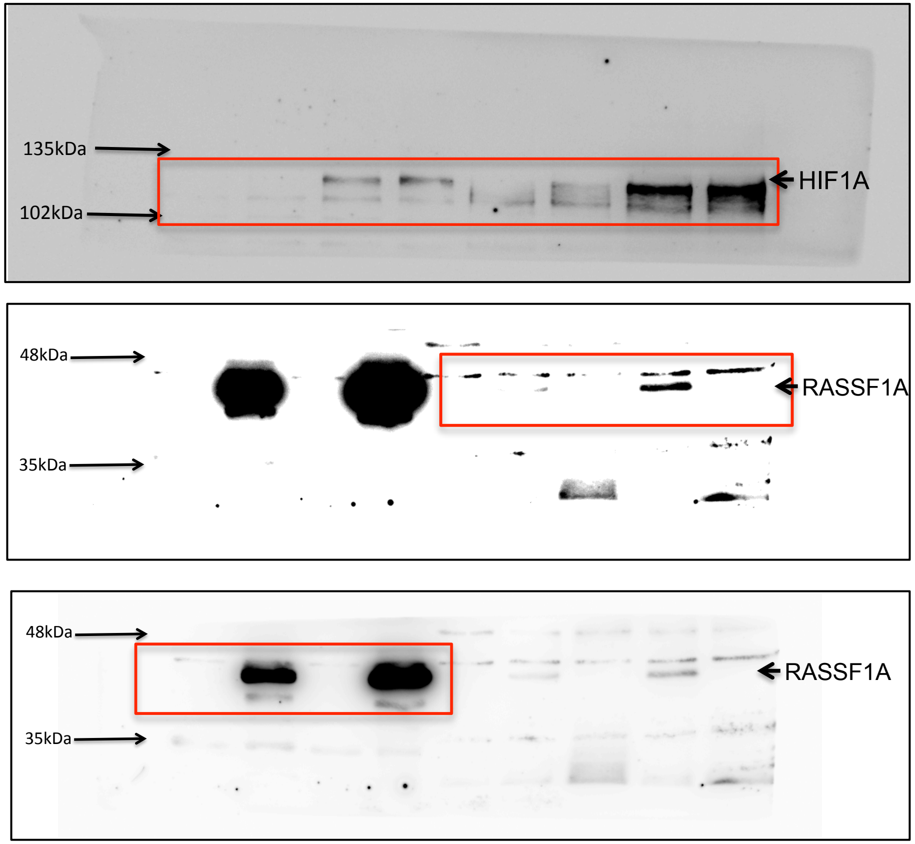

Supplementary Fig 8b

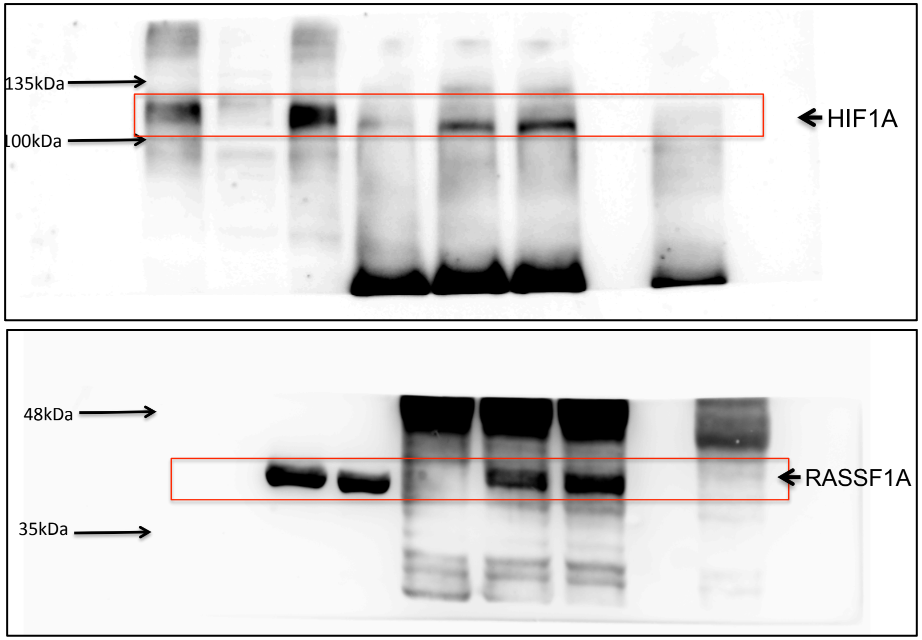

Supplementary Fig 8c

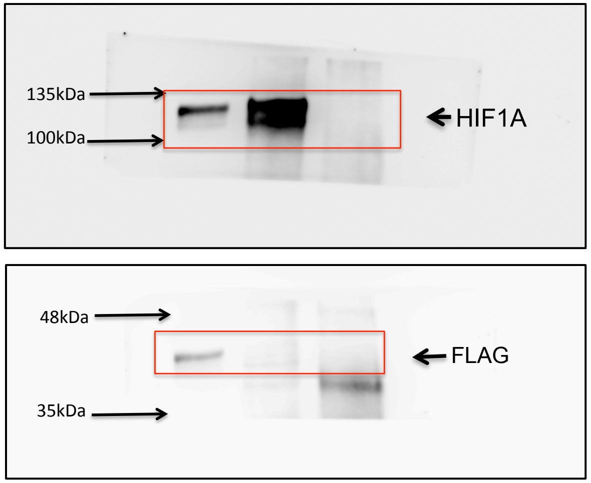

Supplementary Fig 8d

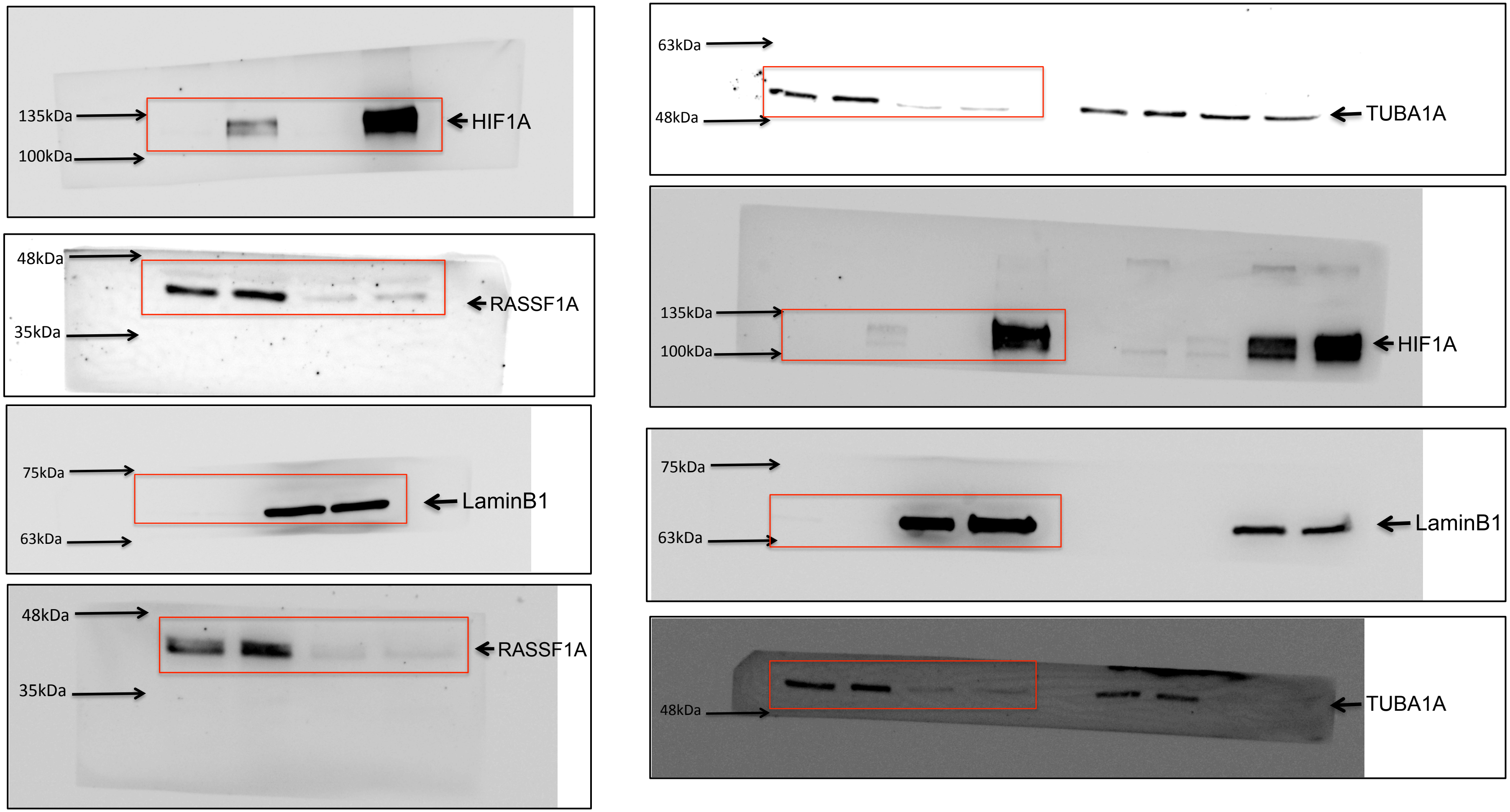

Supplementary Fig 9a

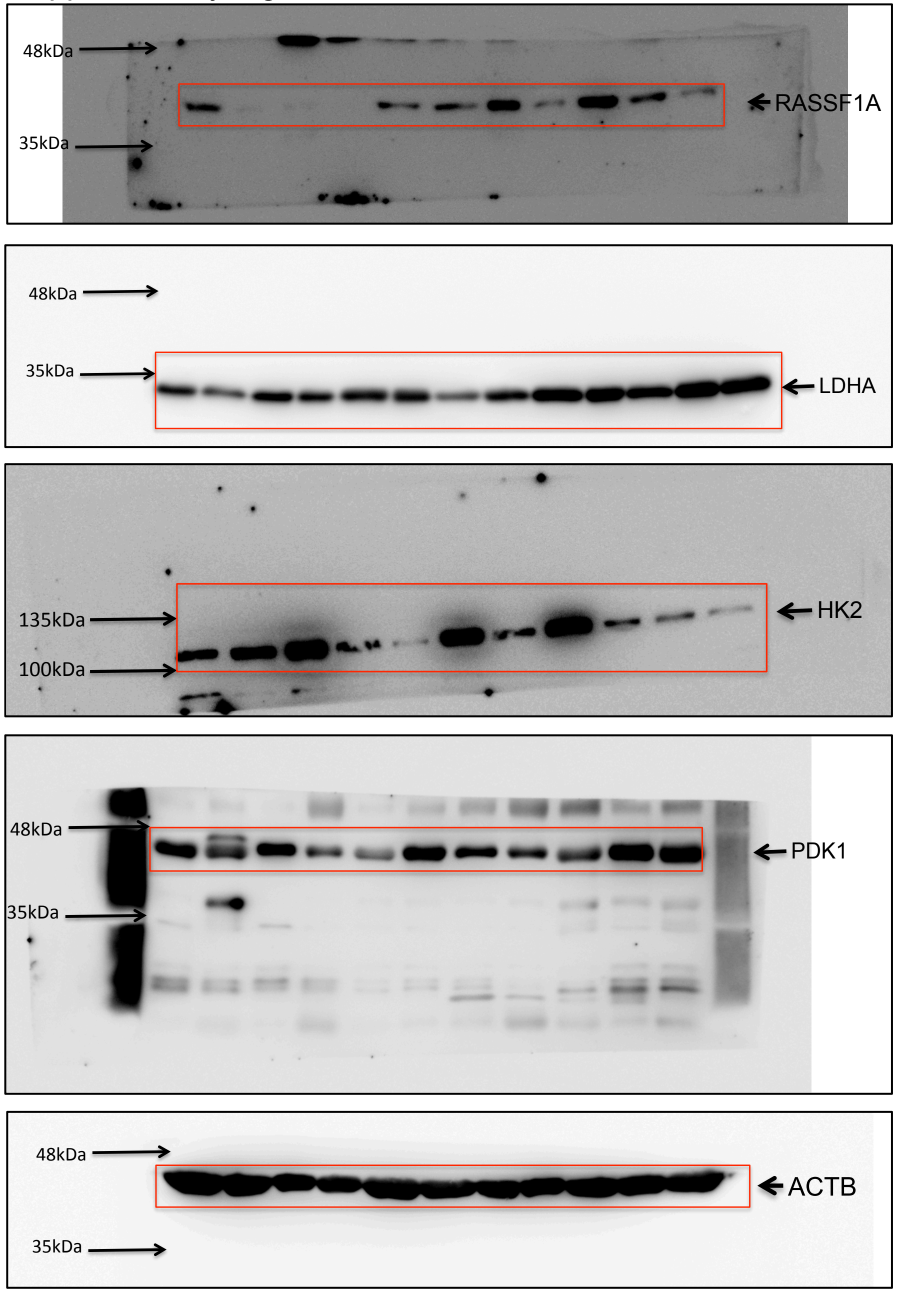

Supplementary Fig 9c

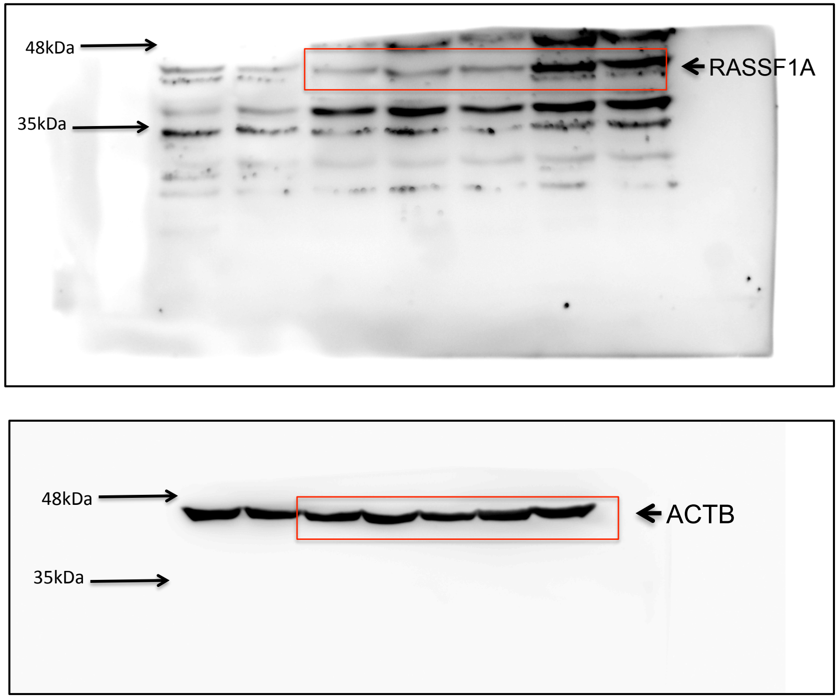

Supplementary Fig 11a

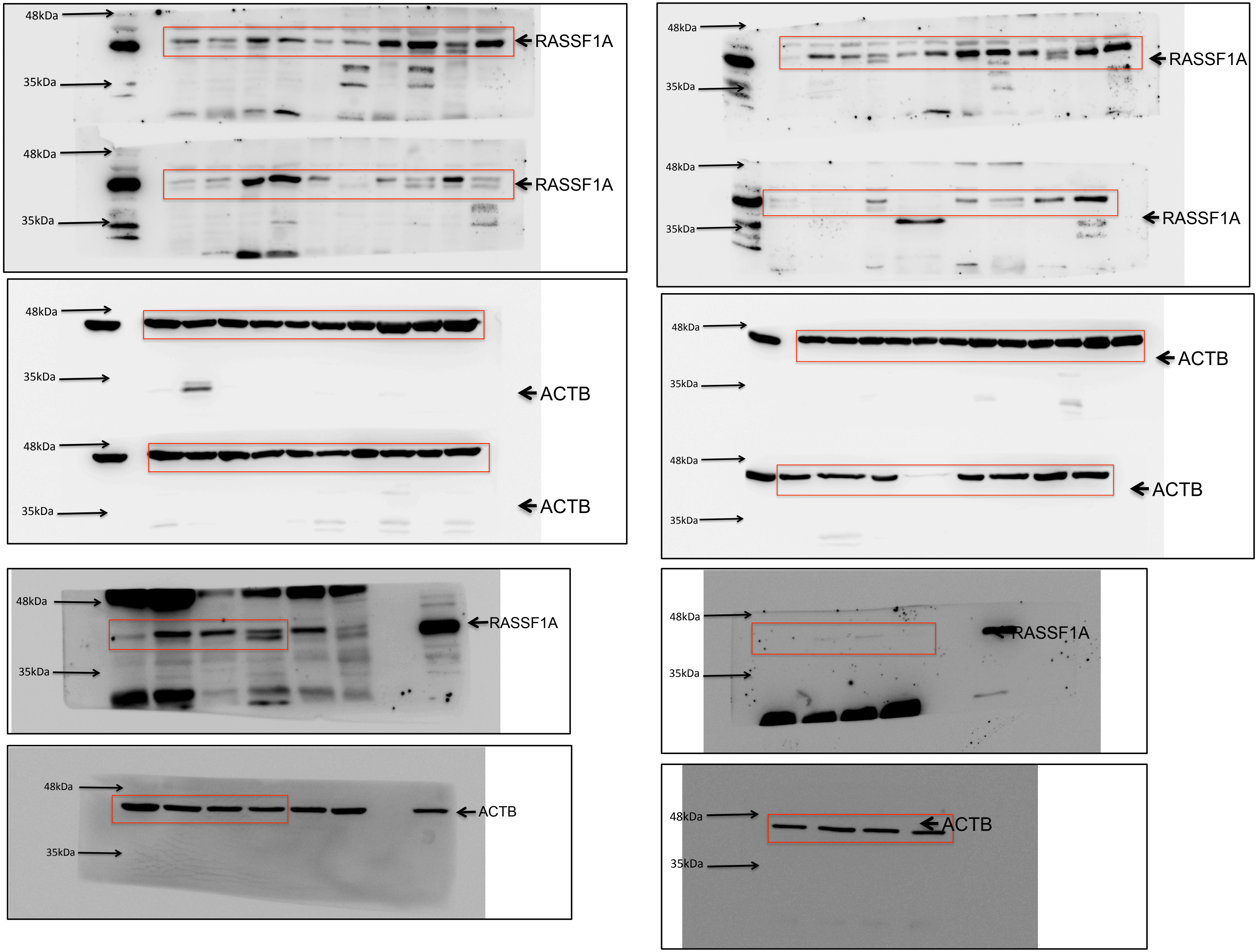

Supplementary Fig 11a

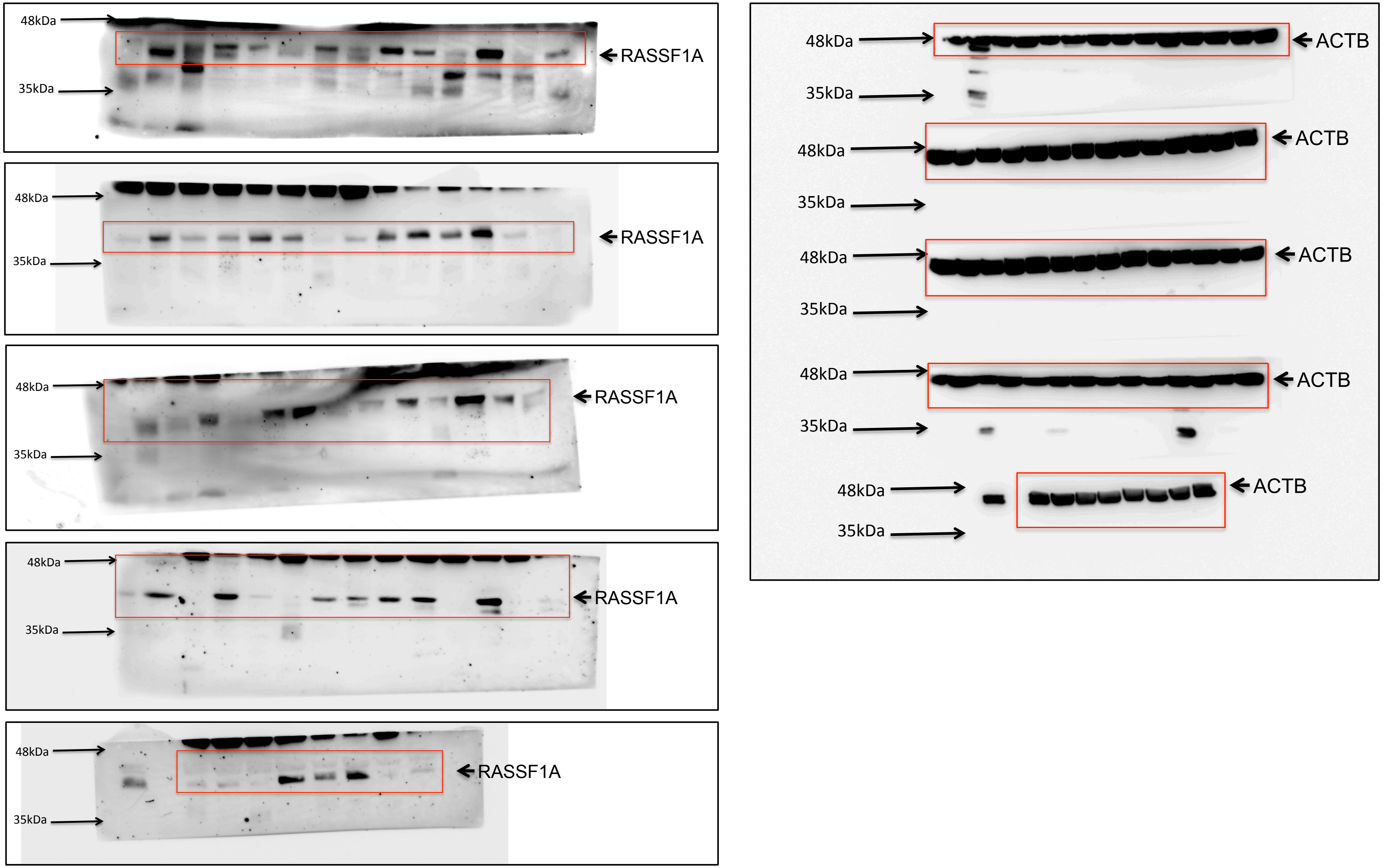

Supplement: Supplementary file 4 — Source Data [file 41467_2019_10044_MOESM4_ESM.pdf]
